# Supplementary material for: Mental health and cultural and linguistic diversity as challenges in school? An interview study on the implications for students and teachers
Source: PLoS One. 2020 Jul 20;15(7):e0236160. doi: 10.1371/journal.pone.0236160 (PMC7371207; doi:10.1371/journal.pone.0236160)
Supplement: S1 Transcripts — (PDF) [file pone.0236160.s001.pdf]

S 1 Transcripts

Excerpts of the transcribed interviews with the externals

Transkription des Interviews mit External 1, Teacher 2

Legende:

Interviewer=„I“; Proband=„P“

Pausen=(...)

unverständliches Wort=((unv.Wort))

Wort- und Satzabbrüche= (-)

- Anonymisierungen=[…]

External 1, Teacher 2

I: Jetzt geht's. Genau, die Einführung, du hattest ja auch die Fragen schon bekommen, also ich steig' dann einfach mal direkt ein. Welche Erfahrung hast du im Umgang mit psychisch belasteten Kindern?

P2: […] über die Schularbeitenhilfe, […] einmal mach ich die Koordination aber natürlich auch die, ich nenn das mal Supervision, coachen. Wie auch immer. Wenn's Schwierigkeiten gibt, darüber. Und natürlich im privaten Bereich über meine eigenen Pflegekinder, […]

I: Hast du Kenntnisse darüber wie sich psychische Belastungen in der Klasse äußern.

P2: Ja, also ich hab ja gerade gestern Name 1 gefragt, weil das nämlich einmal da ganz deutlich ist. Und dieses Mädchen, also einmal gibt es in der, eine unheimlich Unruhe, psychische Belastung und die Kinder klinken sich aus, also bei uns zu Hause war's so, dass Name 2 sich total ausklinkt, aus dem ganzen Geschehen. Also die ist dann einfach gar nicht mehr da, die ist quasi unsichtbar. Und Name 3 macht das genauso. Also wenn es zu schwierig wird, wenn sie zu sehr belastet ist, kickt sie sich selber raus. Das ist die eine Sache. Bei psychisch belasteten, also bei Name 1s Kind war es so, dass sie sich selber versucht zu spüren. Also das macht Name 2 zum Beispiel auch, dass sie ganz viel sich selber, also knibbeln, kauen, verletztten, also auch, Name 3 zum Beispiel hat dann blaue Flecken an den Händen, dass sie sich selber verletzen, damit sie sich selbst spüren können. Das finde ich auch, dadurch dass ich da gestern verstärkt drüber nachgedacht habe nochmal. Da sind ganz ganz viele Parallelen. Oder auch knibbeln, nicht nur normal knibbeln, sondern richtig auf. Dass sie, wahrscheinlich ist das spüren. Blutig knibbeln, sonst geht das nicht. Also, du bist ja Psychologin, du kannst das alles auswerten (lacht).

I: Genau, ich guck mal (lacht).

P2: Ich guck dann mal (lacht).

I: Und welchen Einfluss könnten diese Ausdrucksformen der Belastung auf andere Schüler in der Klasse haben.

P2: Ja, also eigentlich ganz ganz deutlich. Die müssen das alles ertragen. Das ist einmal die Sache. Und was ich noch vergessen habe, was extrem auffällt oben, ist auch sexuelles Verhalten. Also überschrittig, über- grenzüberschreitend. Bei unseren nun nicht gerade aber in anderen psychischen Belastungen, dass das so ist, dass sie (Unterbrechung: 03:00) überschrittig werden aber sich geg-, bei sich selber. Also das ist auch nochmal so'n Thema. Das grenzt natürlich total aus, deswegen komm ich gerade drauf. Weil andere Schüler sich darüber lustig machen, "was ist das denn?". Auch alleine, wenn die knibbeln und die ganze Zeit kauen, das sind natürlich auch so Sachen, wo die Schüler, das fällt denen schon auf. Also das ist nicht nur einfach so, da wird drüber weggegangen. Das fällt auf, das ist besonders. Und die müssen das echt ertragen, Besonderheiten. Also das finde ich ist auch eine Belastung für die Schüler und Schülerinnen.

I: Und welche Herausforderungen kann der Umgang mit belasteten Kindern an die Lehrenden stellen.

P2: Ja, die sind echt gefragt, weil es natürlich so ist, dass sie erstmal zu wenig wissen darüber, was da ist. Die kennen die Kinder ja nur aus der Schule. Und dadurch, dass sie so besonders sind, versuchen sie die natürlich in so ein Raster rein zu schieben. Ich sag das mal jetzt so. Und versuchen sie, durch Besonderheiten (Unterbrechung: 04:15) da auch nochmal, einmal ne Diagnose zu stellen. Das finde ich immer ganz lustig, ne. Also, dass sie so ne Diagnose ganz schnell stellen wollen und dann das Konzept umsetzen, was sie im Kopf haben. Und das funktioniert eben gar nicht. Also das, da sind Lehrer echt gefragt. Also ich finde wirklich, dass sie da

ein ganz ganz großes Potenzial haben an Geduld. Und auch einfach zu gucken, wie, was können wir machen und müssen da eigentlich ganz nah an dem Sorgeberechtigten sein, ich nenn das jetzt mal so. Bei uns ist das ja anders. An den Eltern eigentlich, wenn die Eltern aber nicht in der Lage sind, was oft gerade bei interkulturellen, ist ja diese Problematik, aber auch da müssen sie im Endeffekt ins Gespräch kommen. Also ich glaube, dass das Ganze, wenn es transparenter wäre und wenn mehr Geduld da ist und Motivation, dadurch dass wir ganz ganz viel, wir hatten nun oft, wir waren ganz viel im Gespräch, wir mussten ständig dahin kommen, weil die Lehrerin das nicht mehr ausgehalten hat, dieses Verhalten. Also Name 2 hat das so gezeigt und das ist ja ganz ganz häufig so, dass sie sich so wegbeamen, also nicht mehr da ist. Du kannst sie ansprechen, ist nicht mehr da. Sagst: "Pack mal deine Sachen aus", packt nichts aus. Guckt dich aber an. Und sach mal, und denn reagiert sie gar nicht, also sagt dir auch nicht, warum nicht. Und das musst du ja aushalten. Das ist ja so schwer. Und da mussten wir oft hin. Und dann wurde wieder überlegt, "was können wir tun, was können wir tun". Und nach 1,5 Jahren ist sie dann überprüft worden, sollte überprüft werden und da hat sie sich glaub ich überlegt: "ne, da habe ich keine Lust zu" und dann ist das ganz gekippt. Verrückt, ne? Aber da muss ich der Lehrerin wirklich ein Hoch aussprechen, dass ist wirklich, wirklich ein riesen Lob, weil das ist wirklich so, dass sie die Geduld bewiesen hat, hat sich da ganz lange, also hat da auch nicht reagiert, sagen wir's mal so, hat versucht sie immer weiter anzusprechen. Aber musste natürlich auch, die Klassenlehrerin, mit den ganzen anderen Lehren zusammen kommen, also das ist ja nicht nur eine, gerade in den Weiterführenden Schulen sind's mehrere, ich mein das stimmt auch nicht, in Grundschulen ja auch, es müssen alle an einem Strang ziehen, weil alles andere hätte nicht, wär nicht von, da hät's keinen Erfolg gegeben. Also das ist so klar. Und was so ist, ist dass diese Kinder, wenn du denkst, "oh ja super", also drei Jahre, kann ich dir jetzt so sagen, "ja super, klappt toll" wie's auch zum Beispiel mit, also

Name 2 ist total intelligent, aber ist jetzt auf einem Hauptschulzweig, weil wir alle gesagt haben, "ist besser so". Also sie hätte es auch, mündlich kriegt sie das ja nicht hin, ist ja logisch, "wenn du nichts sagst, kriegst ne 6", das ist nun mal so. Und du kannst ja auch nicht sagen "och ja", weil Mitleid hin Mitleid her, bringt dir auch nichts, bringt sie ja nicht weiter. Und jetzt ist sie auf einem Hauptschulzweig und da gibt es aber auch immer wieder Tiefen. Also es, immer die alten Muster, verfallen die Kinder ja immer wieder. Und das kannst du natürlich als Lehrer überhaupt nicht mehr aushalten. Weil du denkst ja "boah, ich bin derjenige", ich sag das jetzt ganz fies, du erzählst das ja nicht (unv. Wort: 07:31). Also es ist ganz oft so, dass natürlich Lehrer denken "Oh, sie hat reagiert, ich bin diejenige oder derjenige, der 'ne besondere Beziehung hat". Und das klar zu machen, dass das nicht das Thema ist. Es geht hier nicht um 'ne besondere Beziehung. Sondern die Kinder, also wie Name 2, die so psychisch belastet ist, die hat keine Empathie. Die findet das, auch wenn du 'ne besondere Beziehung hat, die hat sie aber nicht. Also auch heute noch, wenn ich sage, ich mein, sie wohnt jetzt, sie lebt jetzt drei Jahre bei uns. Die Mutter sollte, also das ist ein gutes Beispiel. Also Name 2 kannst du wirklich als, kannst du mitnehmen. Die Mutter, sie sollte eigentlich wieder zurück. (Unterbrechung: 08:14) Und sie hat sich für uns entschieden und trotzdem, nur ganz kurz, um das abzuschließen, ist das so, dass sie überall bleiben kann und bleiben kann.

I: Die erste Frage ist: Welche Erfahrungen hast Du im Umgang mit psychisch belasteten Kindern?

P1: Also, aus der Schule meinst Du jetzt?

I: Genau, oder auch allgemein, ja.

P1: Ja, also ich wüsste jetzt, ich könnte das jetzt gar nicht mal psychisch belastet nennen. Ich weiß nur, dass Kinder

eben immer sehr belastet in die Schule kamen, ausländische Kinder jetzt. Aber, auch deutsche, also, und man eigentlich, oder ich dann eigentlich immer gar nicht genau wusste, was da eigentlich passierte. Bis ich dann mit den Eltern gesprochen habe und dann mal so ein bisschen den Familienhintergrund kennengelernt habe. Ja, und dann konnte ich so verschiedene Sachen einordnen. Auch, wieso sie in der Klasse immer am Rande standen. Ja, und so Dinge, die gemobbt wurden von anderen Kindern, was auch häufig vorkam dann. Aber eben auch manchmal von ausländischen Kindern. Es war nicht nur, dass die deutschen die ausländischen Kinder mobbten, sondern es waren eben auch ausländische Kinder dabei, die auch schon wirklich unsere deutschen Kinder gemobbt (-). Was heißt unsere deutschen, deutsche Kinder gemobbt haben. Das wird ja gelöscht.

P2: Unsere! Du ich hab da auch ein paar Sachen gesagt, die gelöscht werden müssen.

I: Wird ja auch nicht jedes Wort jetzt gespeichert, also.

P1: Ja, also, ich weiß jetzt nicht genau, was Du meinst mit psychischer Belastung. Du meinst, dass sie also im Unterricht nicht mitarbeiten können?

I: Genau, es geht jetzt einfach allgemein um belastete Kinder. Es geht nicht darum, dass sie schon unbedingt ne Störung haben, also eine Diagnose, sondern einfach Kinder, die, bei denen man merkt, o.k., da ist irgendwas, die zeigen Symptome von was auch immer. Aber man merkt, o.k., da liegt irgendwas vor.

P1: Ja, also, mehr, häufiger merkt man das ja noch auf dem Schulhof, wenn man Aufsicht macht. Da sind also häufig sind das dann in der Regel Jungen, die sehr aggressiv sind. Und, ja, es ging bis so weit, dass dann

auch der Vater eines Tages, als ich Aufsicht hatte, kam der Vater mit. Da hatte der Junge am Tag vorher Ärger mit einem anderen Jungen gehabt. Und, er war aber nicht unschuldig an der Sache. Er hatte also auch schon ordentlich zugelangt. Und der Vater kam dann nächsten Morgen, und es war gar nicht mein Schüler, ich hatte nur Aufsicht. Und dann hat er also wirklich, richtig Stress gemacht auf dem Schulhof. Und dann hab ich gesagt: „Moment mal, ihr Sohn der hat aber auch, ist nicht ganz unschuldig. Ich hab gesehen, dass er gestern.“ Bloms, vorbei war's, Ende. „Sie sind ja ausländerfeindlich.“ Fertig. Ist er weggegangen. So, und das ist also auch 'ne psychische Belastung, wenn Jungen immer nach Hause gehen und sagen: „Der hat mich geärgert.“ Oder „Der hat mich gehauen“, oder so und es gar nicht immer stimmt. Finde ich. Dass sie nicht die Wahrheit sagen dann. Und bei den Mädchen, die sind ja eigentlich immer sehr, die ich kenne, die ich kennengelernt habe, sie sind ja immer sehr fein und lieb erstmal. Und, aber es zeigt sich dann doch, im Laufe der Zeit, dass sie dann so, eher so im Hintergrund so ein bisschen mobben, andere Kinder mobben. Ja, und was die schulischen Leistungen betrifft, da habe ich zu der Zeit, als ich noch unterrichtet habe, da waren das eben Kinder aus Land 3. Da waren diese Leute aus Land 1 und die, die jetzt in den letzten Jahren gekommen sind, die waren ja noch nicht da. Die habe ich nur dann im, in einem besonderen Kurs gehabt, wo alle auf dem gleichen Stand waren, sozusagen. In der Klasse hatte ich die damals nicht. Aber damals waren es eben Land 3 und aus Land 8. Als der Krieg war, da kamen viele aus dem Land 2 oder aus Land 7. Ja, das war so sehr schwierig, teilweise sehr schwierig. Vor allen Dingen auch mit den Jungen. Die einfach so, ja, die Jungen haben da eine andere Rolle in diesen Ländern, und die Mädchen sind eher angepasst. Und das haben sie dann auch mit hergebracht, ist ja klar. Und werden in der Familie ja auch

so erzogen von ihren Eltern, dass sie Chef sind, sozusagen.

I: Du hast es schon so ein bisschen gesagt, die nächste Frage ist, wie sich psychische Belastungen in der Klasse äußern oder im Unterricht. Ein bisschen hast du es ja schon angedeutet, aber vielleicht kannst du noch mal zusammenfassen.

P1: Nun, oft ist es so, dass sie sich angegriffen fühlten von deutschen Kindern auch, und auch von anderen ausländischen, also von anderen Kindern einfach. Und eben, wenn man dann das aufarbeitete, dann merkte man, das war eigentlich, es war, so im Spielen passiert. Dass die anderen irgendein Wort gesagt haben, ohne sie jetzt beleidigen zu wollen. Kam, die erste Reaktion war ja oft „der hat meine Mutter beleidigt“, diese Geschichte. Ja, was hat er denn gemacht? Was hat er gesagt? "Der Hurensohn, der hat Hurensohn zu mir gesagt". So, das ist also (-). Selber sagten sie das natürlich auch. Da waren sie schon sehr sensibel, oder, da sind sie sehr sensibel auch. Und bei Mädchen, die, da ist es eher so, ich hab da immer ein Mädchen in meinem Kopf, Name 1 heißt die, die wohnt da auch irgendwo, die ist jetzt schon groß, die konnte so leise eben auch andere Mädchen so, ja, mobben, sagt man ja, mobben. Die hat also immer: „Was hast du denn an?“ War oft so subtil, dass man das gar nicht merkte, eigentlich, erst einmal, nachher natürlich.

P2: Weil, was ich so finde ist wirklich, was du auch sagst, mit der Aggressionsschwelle, die ist so gering. Also, wenn ich hier auch auf dem Spielplatz, wenn hier Jungs zusammen sind und auch mit deutschen Kindern, das ist wirklich schwierig. Also einmal diese Ausländerfeindlichkeit. Das habe ich ja auch schon gehört. Nur weil Kritik geäußert worden ist, sagen wir mal so, einfach nur die Grenzen aufgezeigt. Da ist mal gleich in so

einer Schiene drin. Was ich wirklich auch schwierig finde. Also, da muss ich auch professionell bleiben, um nicht verletzt zu werden. Also das ist ja auch so ein Ding. Also, das als Lehrender, das musst du ja auch aushalten. Also und diese Aggressionsschwelle, da hat mir jetzt (-). Wir fahren immer einmal mit den Schülern nach Ort 1, und wenn da was schief läuft, also (-). Ich habe da so ein paar Knirpse da vor Augen. Also, grade mit den Jungs, boh, da geht die Post ab. Da wird es richtig körperlich. Also, das geht ganz, ganz schnell, dass es wirklich zu einer körperlichen Auseinandersetzung kommt. Und das kenn ich eigentlich so nicht. Also, das ist wirklich in dem Kreis extrem. Und das glaube ich auch wirklich, dass das mit der psychischen Belastung halt (-). Das heißt im Umkehrschluss, ich brauch ja keine Diagnose, aber das heißt, ich bin auch nicht so belastbar. Ich weiß auch gar nicht, wie soll ich das zuordnen, sondern das spricht mich sofort an und dann geh ich auch sofort hoch. Da kommen diese ganzen Sachen wie: Hurensohn, der hat meine Mutter beleidigt. Und ich hab das auch versucht, mal aufzudröseln. Also, das hat ne Stunde gedauert und dann hab ich irgendwann gesagt, is gut. Es ist Quatsch und fertig, aufhören.

P1: Aber es ist ja nicht so, wenn man sie hier sieht, an Sonntagen, die haben ja auch untereinander so einen Stress. Es ist ja nicht so, dass sie nur mit deutschen Kindern Stress hätten. Weiß nicht, ob das in ihren Ländern auch so ist. Ich kann das nicht (-). Hab schon oft darüber nachgedacht, ob das in ihren eigenen Ländern, wo sie dann ja wirklich nur unter Inländern sind und keine Ausländer dabei haben, ob sie da auch so reagieren.

P2: Was ich noch mal spannend finde, ich muss das noch mal (-). Das ist für dich umso schwieriger. Aber, das finde ich, ich sag ja, ich hab gestern (-), ich hab ja einen Vorteil, ich habe die Fragen schon gehabt. Und Name 2 ist ja zu

Hause, ist ja Heilerziehungspflegerin in Ort 2. Ich glaube, 80% Ausländeranteile ist in dieser Klasse, und sie selbst macht ja eine Schulbegleitung für ein behindertes Mädchen. Also, und die macht sie aber schon mehrere Jahre.

P1: Behindertes deutsches Mädchen?

P2: Deutsches Mädchen, genau. Und sie sagte, deswegen, das finde ich auch noch mal spannend, es gibt noch so Abstufungen. Also, es gibt wirklich die, also, einmal die ausländischen Kinder. Also, das ist eine riesen Balance, das hinzukriegen und dann kommen aber noch die deutschen, behinderten Kinder, die gar (-).

P1: Die Behinderten, oder die nicht so ganz fit sind (-)

P2: Ja, genau, das wird's ja auch geben, egal. Und dieses mobben, das sagt sie, das ist absolut extrem und wird auch wirklich abgestuft gemacht. Also, die deutschen Kinder, die einigermaßen fit sind in dieser Klasse, mobben dann die Ausländer, die ausländischen noch weiter runter. Also, du suchst Dir immer den Schwächeren noch. Und das rauszufinden als Lehrer ist, glaube ich, ist echt ne Herausforderung.

P1: Ja, es ist ja auch nicht immer so viel Zeit, dass du diese zwischenmenschlichen Dinge, die da ablaufen, kannst ja gar nicht immer einordnen, sofort.

P2: Keine Chance.

P1: Man hat ja auch noch was anderes zu tun. Eigentlich sollte man ja auch noch unterrichten, ja. Ja, und da denke ich an meine letzte Klasse, die ich in der Grundstufe hatte, da war eben auch, da war ein deutsches Mädchen, bisschen schwach, ganz liebe, aber bisschen schwach

auch, aber die war dann wirklich am untersten Ende. Die deutschen Mädchen, die ein bisschen fitter waren, naja, die haben sich gut mit der Name 1, ich sag jetzt den Namen einfach, verstanden. Aber die haben sich dann eben dieses Mädchen rausgesucht, dieses deutsche Mädchen, weil sie so ein bisschen schwach war. Und die war so gutmütig, und die merkte das ja gar nicht.

P2: Ne, das kommt ja auch noch dazu. Ist ja echt schwer. Das ist echt ein Balanceakt, den du da (-). Da fallen dann auch einfach Kinder hinten über, das ist so.

P1: Ja, klar.

P2: Wahnsinn

I: Dadurch, dass ihr jetzt beide miteinander sprecht, so ein bisschen habt ihr die nächsten Fragen auch schon beantwortet. Ich stelle sie trotzdem einfach noch einmal. Genau, und zwar: Welchen Einfluss so psychische Belastungen auf andere Schüler in der Klasse haben können.

P1: Die von ausländischen Kindern kommen?

I: Allgemein psychische Belastungen von Schülern auf andere Mitschüler.

P1: Was die dann auf andere Kinder ausstrahlen, meinst du?

I: Einfach, wie sich das, ob das einen Einfluss auf die anderen Mitschüler hat. Wenn da jetzt in der Klasse jemand ist, der eben psychisch belastet ist.

P1: Also, es gibt jetzt ja genügend deutsche Kinder auch, die familiäre Belastungen haben, und die bringen sie

schon mit in die Schule. Und man merkt ihnen das auch an. Wenn sie dann, ja, (-). Manchmal ist es dann ja auch so, dass sie einem was erzählen. Nicht so ohne weiteres aber. Ein Mädchen hatte ich mal, die hat dann auch ganz offen darüber gesprochen, aber nur mir gegenüber. Und, ja also doch, viele Belastungen gibt es eben auch, wenn Trennungen erfolgen. Der Vater, ist ja meistens, Alkoholiker ist (-), haben sie auch (-). Das merkt man den Kindern auch sofort an. Sie sind dann häufig in sich gekehrt, oder manche dann auch eben aggressiv. Den einen, denke ich jetzt grade an den einen Jungen, der aus Land 1 kam, wo der Vater sich nicht kümmerte, die Mutter alleine mit zwei Jungen war und der Vater sich nicht kümmerte. Und der war richtig aggressiv dann, auch den anderen Kindern gegenüber. Obwohl das ein ganz (-). Ich mochte den so gerne, aber er konnte sich einfach manchmal nicht steuern. Ich denke, das lag an der Situation.

P2: Aber, wie war das denn für die anderen Kinder? Das finde ich ja auch nochmal (-). Also, weil ich in Name 3 ja ein gutes Beispiel habe. Also, die mussten, also, die Klasse muss ganz viel auffangen, um so ein Mädchen mitzunehmen. Also, entweder, die fällt hinten über, das kann natürlich auch passieren. Aber, da, dann ist sie eben Außenseiter, also von ganz raus. Ich glaube, es gibt auch nicht so halb drinnen. Also, das kann ich mir nicht vorstellen. Aber, wenn es so ist, dass sie mit (-), also, dass die Klasse recht sozial ist, oder homogen aufgestellt ist, sagen wir es mal so, dass sie so ein Mädchen mitnehmen können. Das heißt aber auch, dass sie wirklich (-)

P1: Tun sie das auch?

P2: Das ist die Frage, das würde mich interessieren. Also, bei Name 3 hat das ziemlich gut funktioniert. Aber ich

glaube, weil sie nicht, weil sie schon auffällig war auf eine Art und Weise, aber nicht so aggressiv.

P1: Das wollte ich sagen, das ist wahrscheinlich der Unterschied.

P2: Wahrscheinlich liegt es auch daran, was da passiert. Ob die immer streiten müssen, oder (-)

P1: Aber, wenn die dann so, eigentlich ein ganz liebes Mädchen ist, dann nehmen die anderen sie auch an, glaube ich. Aber wenn die immer Aggressivität ausströmt, sozusagen, dann (-)

P2: Ne, wer will sie haben, also, das will ja keiner.

P1: Aber ich denke jetzt auch noch mal an verschiedene Kinder, die ich auch in der Klasse hatte, das waren deutsche, die in schwierige Verhältnisse hineingeboren wurden und dann als Pflegekinder in die Klasse kamen. Da gab es eben auch ganz ganz schwierige Jungs, hauptsächlich Jungs dabei. Und der litt unendlich. Ich mochte ihn ganz gerne, aber der war so aggressiv, auch den anderen, nicht nur mir auch manchmal gegenüber, aber auch den anderen Kindern. Dann lief er immer weg und, das war ganz schlimm. Der ist, also (-). Da hat die Klasse auch sehr drunter gelitten. Also, sie hatten dann teilweise auch Angst, weil er durchaus auch dann körperlich tätig wurde. Oh Gott.

P2: Oder die werden in so eine Schiene reingesetzt. Also, Der Clown, das ist ja klar, der ist so.

P1: Naja, er war ja kein Clown.

P2: Ja, aber das gibt's ja auch.

P1: Einerseits aggressiv, andererseits lag er da und nuckelte, dann lachen sie ihn aus.

P2: Ja, genau, aber das ist ja auch so was, das sind ja auch so körperlich (-). Also, ich hab zur Bespiel grade auch schon gesagt, dass also dieses knibbeln, richtig blutig knibbeln, dass das bei Kindern ganz oft ist und das grade bei den belasteten Kindern, die sich wirklich, ja, dann selber spüren müssen, irgendwie. Vielleicht hinterher auch. Das ist ja in späteren Jahren erst mit dem Ritzen. Obwohl das in der Schule 1 auch schon aufgetreten ist, das (-)

P1: Schule 1 und Schule 2 sowieso. Da hatte ich auch Schüler.

P2: Die sich dann eben selber spüren und (-). Einerseits ist das natürlich eine Aufmerksamkeit von den anderen Kindern, das ist auch super, wie auch immer. Und, aber auch natürlich, "das ist mir unheimlich". Das denke ich auch.

P1: Also, das ist mir auch unheimlich, wenn Mädchen, sich ritzen. Ja hatte ich auch, zwei Kinder, zwei Mädchen, aber das sind immer Mädchen, die ritzen. Da fallen mir jetzt erst so Sachen wieder ein. Es ist alles schon so lange her, aber dann wird's eben auch (-), ja. Aber andererseits hatte ich ein Mädchen, das war das vierte von vier Kindern, und die kam eines morgens in die Klasse (-), habe ich dir, glaube ich, schon erzählt, das vergesse ich nie. Die war immer so ausgeglichen und Name 4, die war immer so nett. Alle wollten mir ihr spielen. Und eines morgens kam die zu mir in die Klasse. Ich wusste von deren schwierigen Verhältnissen. Und dann sagte sie: “Frau P1, Mama hat neuen Freund.“ Und da „Ja, schön, ist er denn nett?“ „Ja, nett ist er.“ Und dann sagte sie aber, "er säuft auch, aber er haut nicht.“ Ja, aber dieses Kind, das ist in solchen

Verhältnissen groß geworden, die Mutter trank bestimmt auch. Und die war ausgeglichen, ich kann es mir nicht erklären. Und die hat das auch durchgehalten. Die meisten Mädchen, aus unserer Schule zumindest, die sehen ja ganz schnell zu, dass sie schwanger werden. Das ist auch so eine Sache, die sie dann, damit sie was Eigenes haben für sich (-). So erkläre ich das. Die sind ja manchmal schon in der achten, neunten Klasse schwanger und haben dann was Eigenes. Und da war auch eine dabei, ach so, und dieses Mädchen, die mit dem Freund der Mutter, die hat das, "nein", hat sie gesagt, "ich will doch nicht gleich ein Kind haben". Ganz bewusst. Und ich traf sie, letztes Jahr traf ich sie. Die ist ja jetzt mindestens 25, 26, traf ich sie in Straße 1. Ich sag: „Oh, Name 4, wie geht es dir?“ „Ja, gut, ich bin jetzt auch schwanger.“

P2: Das ist natürlich schön.

P1: „Aber ich hab auch nen Mann“, hat sie dann gesagt.

P2: Ach wie schön.

P1: Ja, und bei den anderen Geschwistern ist es alles daneben. Bei den drei anderen ist alles danebengegangen. Gleich Alkoholiker, (-)

P2: Ja, das ist eben auch die Schwierigkeit.

P1: Ja und dann habe ich noch die, die mir auch grade einfiel, die ist schon in der fünften, sechsten Klasse, die war in der Grundstufe bei mir. Die war auch sofort schwanger. Aber das waren auch so schwierige Bedingungen zu Hause. Also, der merkte man das in der Schule immer an, wenn da was los war. Und die ließ das dann auch so aus sich raus, die war nicht in sich gekehrt,

sondern die war dann richtig frech und aufmüpfig, beleidigend, den anderen gegenüber.

P2: Ja und das macht echt Angst. Also, das glaube ich, als Mitschüler macht das Angst, das ist unheimlich.

P1: Ja, die können das ja auch nicht einordnen.

P2: Ne genau, Dann ist die Frage, wenn die das auch nicht kennen, da kommen ja nun alle da zusammen. Das ist ja auch gut so, Und die das nicht kennen von zu Hause, sehr, sehr unheimlich. Das finde ich auch, würde mir auch Angst machen. Wüsste ich auch nicht, wie ich damit umgehen soll. Und insofern wende ich mich ab, das ist das Nächste.

P1: Ja, sind dann Außenseiter.

I: Welche Herausforderungen entstehen denn für Lehrende, wenn Kinder in der Klasse psychisch belastet sind?

P1: Ja, man muss sich einfach mehr kümmern um die Kinder auch, wenn es denn möglich ist. Manchmal ist es im Unterricht (-). Man kann ja auch nicht auf alles eingehen. Aber hauptsächlich ist es immer nach den Pausen gewesen, so. Wenn was anlag, dann wurde das besprochen. Und, ja, soweit es ging, dann geregelt, beziehungsweise, sich entschuldigen müssen und was man dann so alles sagt. Hat man das angehört, was die beiden Kampfhähne da miteinander hatten. Oder, wenn das auch jemand aus der anderen Klasse war, dann sind wir da auch hin gestiefelt und haben dann dies und jenes (-). Einmal hat ein Junge einen anderen bespuckt auch auf dem Schulhof. Und der kam dann nach der Pause zu mir und sagte: „Der hat mich bespuckt." Name 5, das war auch so einer. Dann bin ich mit Name 5 zu der Klasse.

Lehrerin 1 war das, die alte Lehrerin 1. Dann musste Name 5 sich entschuldigen. Und dann sagte sie einfach: „Das macht man aber nicht, du bist doch kein Lama.“

P2: Ja, cool

P1: Und da war es wieder gut. Ja, das hängt auch immer ein bisschen vom Charakter der Kinder ab, wie dann (-). „Ne, ich entschuldige mich nicht.“ Er ist ganz bereitwillig dann mitgegangen. Das weiß ich jetzt auch nicht, warum die Kinder dann manche (-). Obwohl er ja nun gespuckt hat, er hat wahrscheinlich seine Schuld erkannt. Aber manche weigern sich ja auch, auch wenn sie schon zum Teil Schuld auch hatten. Wollen das nicht klären. Zuerst mal ne.

P2: Also, was ich finde eben halt auch nochmal deutlich zu haben, das nicht persönlich zu nehmen. Also so was geht ja noch, wenn das untereinander ist. Aber wenn du selber als Lehrende da angegriffen, dich angegriffen fühlst, egal, ob das jetzt mit spucken, das ist ja schon körperlicher Angriff sozusagen. Aber was ich auch vorhin gesagt habe mit Name 3, dass du nicht anfängst, es persönlich zu nehmen, dass es an dich gerichtet ist. Weil, wenn das passiert, und da brauchst du eigentlich als (-), wenn du so einen Zündstoff hast in der Klasse muss eine Supervision her, um das klar zu haben für dich. Was ist das eigentlich? Was spricht mich da jetzt so an? Das geht an meine Ehre vielleicht. "Ich geb' doch alles". Und "wie undankbar". Und "das funktioniert nicht, das was ich alles aus meiner Tasche hole, nichts, oder aus meinen ganzen letzten Erfahrungen. Das zieht nicht". Und das, finde ich, ist echt eine Herausforderung. Nicht zu kippen und in dem Moment, wo es kippt, hatten wir ein paarmal, da ist es wieder wichtig, zurückzukommen in die Professionalität. Nicht zu sagen, "das ist gegen mich gerichtet". Und das ist wirklich, ich glaube, da ist die Krux auch ganz oft bei

Lehrenden. Die dann auch kaputt darangehen, an diesen, an solchen Klassen.

P1: Oh ja, an den Klassen und auch an den Eltern. Da erinnere ich mich nämlich auch an ein Elternpaar. Die hatten einen Sohn, und angelblich wurde er immer geärgert und immer geärgert. Das war so furchtbar. Und dann habe ich dann eben auch gesagt, zu denen (-). Die kamen dann und beschwerten sich natürlich. Ich sach, "ihr Sohn ist natürlich auch nicht ganz unschuldig". Aber sie hielten ihm einfach immer die Hand vor den Hintern. Die ganze Zeit, waren immer die Anderen. Aber da haben wir auch (-). Ich hab da keine Basis gefunden zu denen. Zu dem Jungen wohl. Wenn der ohne Eltern auftrat, das war immer in Ordnung. Aber die Eltern (-). Also, ich weiß nicht, was es war. Dann habe ich ihn später mal, da war er schon erwachsen, getroffen. Das war auch, wir haben uns immer gut verstanden. Nur, wenn er eben dann ärgerte, dann (-). Oder ärgerte und geärgert wurde auch, dann erzählte er das zu Hause. Und die Eltern üben ja dann nicht immer (-). Also, die hinterfragen das ja nicht immer. Die meinten da einfach immer, die anderen. Also, manche.

P2: Naja, und wenn dann die Angriffe von denen kommen, auf mich als (-)

P1: Ja, das macht einen fertig.

P2: Das macht, ja genau, da muss man eben halt wirklich so weit sein, oder drüber nachdenken, oder immer wieder reflektieren, was ist das jetzt eigentlich? Was meinen die Eltern? Meinen die wirklich mich? Meinen die das System, wie auch immer. Was ist da (-)? Wie kann ich davor auch entfliehen? Geht, funktioniert nicht immer. Das geht nicht, das ist ja logisch.

P1: Wenn ich an dieses Elternpaar denke, das funktionierte überhaupt nicht mit denen.

P2: Aber, das ist ja eine Entscheidung. Ich finde, da fehlt ja bei Lehrern, also eine Reflektion. Habt ihr das in der Schule? Gibt es das?

P1: Ob ich Reflektion, als Supervision? Nein. Also, ich weiß ja, ich bin ja jetzt lange raus, aber wir haben schon über solche schwierigen Probleme auch gesprochen und (-). Also eine Supervision war zu der Zeit noch nicht. Aber es wurde dann angeleiert. Ich weiß gar nicht, wie das jetzt in Schule 1 ist.

P2: Ich glaub das nicht.

I: Das wäre auch meine nächste Frage tatsächlich, was es für Unterstützungsmöglichkeiten gibt. Ja, oder gibt es Unterstützungsmöglichkeiten? Und wenn ja, welche?

P1: Also, mich hat damals mein Schulleiter unterstützt. Bei diesem Elternpaar, das war also schon, war wirklich, da war ich auch am Rande. Weil die immer wieder so, (-) Ja, das war unmöglich, dass sie immer nur ihren Sohn sahen und nie mal auch hinterfragten, ob der Sohn denn wohl richtig gehandelt hat, oder nicht auch einen Anteil trägt. Und mich beschuldigt haben, weil ich ja immer ihn nicht wirklich unterstützt hatte, sagten sie.

P2: Naja, und in dem Moment wenn man Berufsanfänger ist. Es kippt ja auch wieder viel, es sind ja viele Berufsanfängerinnen und -anfänger. Dass die sich da auch persönlich überfordert sehen, das ist ja keine Frage.

P1: Natürlich. Unglaublich

P2: Ich habe das noch nie gehört, dass eine Schule Supervision macht. Das kenne ich nicht, aber, oder gecoacht werden, oder wie auch immer.

P1: Das ist ja bald länger im Gespräch gewesen, schon als ich noch in der Schule war, wurde das immer wieder mal thematisiert. Ich weiß nicht, ob sie es irgendwann dann gemacht haben.

P2: Ja aber, das müsste Name 6 ja auch wissen. Also, so (-)

I: Also, hab ich auch nie von gehört, dass es da sowas gab.

P1: Vielleicht eher mit Förderschulen dann. Weiß ich nicht, ob die das machen.

P2: Das könnte eher sein. Aber das ist ja grade in den Schulen, da wird es ja immer schwieriger, auch in den normalen allgemeinbildenden Schulen. Also, deswegen finde ich das schon (-).

P1: Ja

P2: So eine Reflexionsrunde, oder, wie auch immer man es nennt.

P1: Wir hatten mal eine Zeit lang die Psychologin, die Schulpsychologin bei uns an der Schule. Die machte manchmal so Nachmittage. Also, als ich auch noch da war. Und dann wurde immer ein Kind, beziehungsweise ein Problem auf den Tisch gelegt, von irgendjemandem dann. Und das wurde dann, (-). Aber nicht in der Klasse jetzt, das man in der Klasse beobachtet wurde dabei. Das ist es doch, Supervision, was du meinst.

P2: Naja, Supervision mein ich sogar, dass du, wenn ich jetzt, gutes Beispiel, du mit deinem Kind da, mit dem mit den Eltern und ich geh nach Hause, also wenn ich jetzt kein gutes Umfeld habe, dann trage ich das mit mir mit. Also, das geht ja gar nicht anders. Ich kann das eben halt nicht für mich sagen, "oh ja, Mensch, das muss ich verändern, damit mich das nicht so angreift". Weil, das ist ja immer das Problem. Du nimmst es irgendwann persönlich, dann greift dich das an und das nagt an dir.

P1: Das wirkt sich dann aufs Kind aus.

P2: Aufs Kind, auf die ganze Schule, auf dein ganzes Arbeitsverhältnis. Genau, dass du einen Horror hast, irgendwo hinzugehen und in eine Klasse zu gehen, weil eben dieser eine Schüler dasitzt, oder so was. Und so was, als Lehrer habe ich das Gefühl, das ist natürlich jetzt wirklich sehr (-), dürfen sich diese Blöße gar nicht geben. Also, das darf gar nicht. Das darfst du im sozialen Bereich. Also, Sozialpädagogen und so weiter, die dürfen das tun. Die können sich melden und sagen, ich kann nicht mehr. Ich habe eine Gruppe da, das ist schwierig. Ich brauche mal eine Supervision. Da funktioniert es. Also, in Kindergärten, in Kitas, wird das ja auch gemacht, Lehrer dürfen es nicht. Also, die müssen da irgendwie mit klarkommen. Da wird so war, die haben vielleicht den Schulleiter, wenn du Glück hast. Das ist ja auch nicht immer. Der sagt vielleicht mal: „Heute habe ich keine Zeit, nu mach mal, du machst das schon." Vielleicht hast du ein Umfeld, deinen Mann, oder was auch immer.

P1: Mein Schulleiter, der hat mich schon unterstützt in der Beziehung. Später auch, als es mal total explodierte, sozusagen, als ich dann beschuldigt wurde sogar eines Tages. Was war denn das, ein Sportfest oder so? In meiner Klasse, da hinten in der Schule 2, da hatten wir ja so Terrassen, und mein Schlüssel war weg, als alles zu

Ende war. Und dann, ich sach "oh wat". Ich hab ja gar nichts gedacht. Ich dachte, ich hätte, ich hätte ihn verbaselt. Dann sagte ein Junge mir: „Den hat Name 7“. Name 7? Ich hinter Name 7 her, der musste zum Bus. Habe Name 7 fast aus dem Bus gerissen. „Name 7, wo ist mein Schlüssel?“ „Ich hab den nicht.“ Und dann ist er in den Bus. Und dann fand ein anderer Junge den Schlüssel, den hatte er da grade hingeschmissen, beim Bus. So, Name 7 hat dann irgendeinen Anschlussbus nicht mehr gekriegt. Dann rief Mutter an bei meinem Schulleiter. „Frau Lehrerin P1 hat dies und jedes und Name 7 konnte, musste da eine Stunde warten", oder was weiß ich, was der musste. Jedenfalls, da gab es dann auch ein Gespräch mit den Eltern. Und da wurde das aufgearbeitet. Und ich hätte ihn gehauen auch noch.

P2: Ja, oh Gott, ja. Und dann bist du ja dran. Das kannst du ja vergessen. Ich mein dafür ging's vielleicht noch. Heute kannst du gleich deine Tasche nehmen.

P1: Das war das Schlimmste ja noch, ich hätte ihn gehauen. Naja, und dann wurden sie einberufen. Und in der Zwischenzeit hatte sich das aber nach ein paar Tagen hatte sich das rausgestellt, dass Name 7 (-). Ich weiß gar nicht mehr genau. Es war wirklich (-), das hat mich sehr belastet. Weil, ich habe nie ein Kind gehauen. Weder meine eigenen (-)

P2: Aber das ist, das sind die Punkte. Es ist im Endeffekt, das müssen Lehrer mit sich selber ausmachen. Und ich habe, wir haben ja nun auch genügend in der eigenen Familie (-), das ist ja immer so ein Spruch, das sage ich ja auch, ein leichter Vorwurf: Ihr wisst es ja sowieso, wie es geht. Lehrer wissen immer alles. Ja, es ist ja auch ein Selbstschutz. Das ist ja, weil du ständig in Diskussionen kommen musst und dich ständig hinterfragen musst, ist das richtig, ist das falsch, geht’s ja gar nicht. Also,

irgendwie musst du dir ja auch so einen Wall aufbauen. Aber das finde ich wirklich schwierig. Und das hat natürlich im Umkehrschluss auch wieder mit Haltung zu tun. Auch den Beruf, dass ich, dass mir auch Sachen passieren können, die nicht richtig sind, wie auch immer. Aber da auch ins Gespräch zu kommen und auch das sagen zu dürfen, das ist schief gelaufen, da gehört ja auch was zu.

I: Was könnte es denn noch für Unterstützungsmöglichkeiten geben für Lehrerende oder auch Schüler, oder im Unterricht, um einfach den Umgang mit psychischen Belastungen im Unterricht zu erleichtern?

P1: Also es gibt ja, mittlerweile gibt es ja jede Menge Unterstützung in den Klassen. Ich weiß gar nicht, wie viele Personen in den Klassen sitzen, manchmal mehr Personen, also in Förderschulklassen, als Kinder. Förderschulklassen gibt es ja nicht mehr. Das finde ich dann ja auch schon (-). Also, ein oder zwei Leute und direkt neben dem Kind, was dann da grade so problematisch (-). Auch nicht immer, weil, das wird ja dann wieder stigmatisiert. Aber wenn ich zu der Zeit, als ich noch gearbeitet habe, dann gab es ab und zu Praktikantinnen oder dann mal zwei Wochen oder so von der BBS und wo sie herkamen, das war in der Regel immer ganz gut, weil, die konnten sich dann einfach mal neben ein Kind setzen und das deswegen beruhigen, nur weil sie da saßen, während die Kinder arbeiteten. Aber jetzt weiß ich es nicht. Also, ich kann es ja nicht mehr sagen. Ich weiß nur von meinen Kolleginnen, dass da manchmal fünf oder sechs Leute in der Klasse sitzen.

P2: Also, Name 2 hatte ich ja auch gefragt. Ich fand das wirklich spannend, weil, das ist ja eine ganz andere Sache. Das ist ja keine Lehrende in dem Sinne, sie hat ja nur dieses Kind was sie begleitet und muss das aber auch beschulen. Also, das ist so eine Mischung. Und das ist ja

noch eine Krux, weil nämlich die Förderschullehrerin, mit der hat sie ganz große Schwierigkeiten, weil die sagt, du kannst das nicht. Du bist ja nur Heilerziehungspflegerin, du kannst das gar nicht. Obwohl sie die in- und auswendig kennt, dieses Mädchen. Ist da auch zu Hause. Das musst du aushalten. Aber sie sagt eben, was ganz klar fehlt, ist eine Transparenz. Also, die Lehrer wissen also ganz viel zu wenig. Und das dann auch akzeptieren, zu akzeptieren, dass es eben halt auch Leute gibt dabei, die das unterstützen können. Und ein Miteinander, das ist natürlich auch unterschiedlich, aber das erfährt sie. Also, in der einen Schule zum Beispiel, war das auch eine ganz klare Abstufung. Die Schulbegleiter mussten, hatten einen kleinen Extraraum, durften nicht mit ins Lehrerzimmer. Das ist da jetzt anders. Jaja, das ist da jetzt anders. Aber nichts desto trotz ist auch mit der Klassenlehrerin nicht dieses Problem, aber Förderschullehrerin. Also, das sind auch so Altlasten, glaube ich einfach. Position zu erkämpfen, was gar nicht notwendig wäre. Wenn das klar ist, so eine Transparenz, was sind eigentlich die einzelnen Aufgaben, und eine Transparenz, was ist eigentlich mit diesem Kind? Also eigentlich, wenn es jetzt grade auch ein Kind mit Handycap ist, klar zu haben, das und das und das ist das. Also, dass es nicht nur so ein Makel ist. Oder auch eben auch diese interkulturelle Sache, einfach klar zu haben, was kann eigentlich sein. Aber das auch als normal zu sehen. Also nicht immer dieses, diese riesen Blase da drum herum.

I: Wenn wir jetzt schon beim Interkulturellen sind, was gibt es denn für spezielle Herausforderungen in interkulturellen Klassen, also in Klassen in denen Schüler verschiedener Kulturen zusammen unterrichtet werden?

P2: Also, da ist einmal überhaupt der kulturelle Hintergrund. Das ist einmal mit den Jungs (-)

P1: Die Jungs, das hatten wir ja schon gesagt, dass die einfach so vorneweg preschen. Wobei es da auch Unterschiede gibt. Aber ich habe im Moment ja einen Schüler, den ich jetzt betreue, der ist ja sowas von vorbildlich, die ganz Familie. Ja also, in der Regel, die Jungs, die (-)

P2: Naja, die verschiedenen Kulturen, die ja gerade da aufeinander knallen.

P1: Untereinander sind sie sich ja auch nicht so ganz grün. Ich weiß es ja hauptsächlich nur noch von den Kindern, die aus Land 2 kamen, oder Land 3. Also, in der Regel verstehen die sich schon, die Jungs auf alle Fälle. Wenn sie dann nicht allzu aggressiv sind. Ich meine, da ist mir nicht so aufgefallen, dass sie so die deutschen mit den ausländischen Jungen Probleme hatten, wenn die sich normal verhielten. Wohl, wenn sie dann wirklich sehr aggressiv waren. Das ist klar. Das führte dann immer weiter und sagte „du bist ja ausländerfeindlich“ und solche Sachen. Ich kann das gar nicht so, in der Klasse fand ich das nicht.

P2: Ich finde, was hier doch ganz deutlich ist, ich war ja jetzt am Samstag auf dem Spielplatz, deswegen weiß ich das, mit unseren Kindern. Und da ist eben noch ganz klar eine ganz klare Cliquenwirtschaft. Das ist bei den Mädchen ein bisschen aufgelöster. Also hier ist das noch ganz deutlich. Hier aus dem Viertel, sage ich jetzt, ganz viele aus Land 3,

P1: Die wohnen aber auch zusammen hier.

P2: Genau, und die tun sich ja auch zusammen. Das sind die aus Land 4, die sich zusammentun, die aus Land 5, Land 1 und so weiter. Und das aufzu (-). Und auch nicht die Vermischung mit den Deutschen. Das war bei den

Mädchen anders. Das war ein gutes Beispiel da. Da waren ein paar deutsche Mädchen dazwischen, aber ganz wenig. Und das denke ich, ist noch einmal ein Riesenthema. Integration hin, Integration her, wie funktioniert's.

P1: Wie die untereinander auch zurechtkommen.

P2: Ja, genau, wie kann das denn auch untereinander klappen? Und das ist hier ja auch so, dass hier die Deutschen sich zusammentun. Auch aus dem Viertel, die deutschen Mädchen oder die deutschen Jungs und die ausländischen Kinder. Also, da ist noch ein ganz großer Weg. Bei den Land 6, aber das ist ja nun auch schon 50 Jahre her.

P1: Ist das ja nun auch schon die zweite Generation, oder die dritte sogar.

P2: Da weicht sich das auf. Aber, oder und, ist trotzdem so.

I: Gibt es denn spezielle Herausforderungen für die SchülerInnen in den Klassen, wenn verschiedene Kulturen da zusammenkommen? Irgendwelche Einflüsse?

P1: Dass der eine immer das Butterbrot von den anderen aß, weil der (-) Also, innerhalb des Unterrichtes? Also für Lehrende jetzt, was den Unterricht betrifft? Oder das miteinander.

I: Oder auch die Schüler. Einfach ob das irgendwelche Einflüsse hat. Ob der Unterricht für die Schüler in einer Klasse, in einer interkulturellen Klasse vielleicht anders ist als in einer Klasse, wo jetzt hauptsächlich deutsche Schüler/innen sind.

P1: Dafür ist das für mich jetzt zu lange her. Ich hatte nicht diese ganzen Kinder, die jetzt da kommen. Aber damals waren das Kinder aus Land 3, die sprachen schon gut deutsch. Also und eben aus Land 3. Naja, das war ja nun auch Förderschule, die hatten schon auch große Schwierigkeiten. Aber das meinst du eigentlich nicht, ne?

P2: Aber doch, weißt du, wenn du das, im Unterricht glaube ich, ist das nicht so das Thema. Weil du im Unterricht natürlich Hilfen hast.

P1: Man hat ja eine Struktur da.

P2: Genau, da ist die Struktur klar. Also, das ist dann eher auf dem Sportplatz, auf (-)

P1: Auf dem Spielplatz oder auf dem Pausenhof.

P2: Ja, Pausenhof und so weiter, was du vorhin beschrieben hast. Und Sportplatz, da hatte ich dann auch mal so eine Szene, da habe ich auch versucht zu intervenieren. Das hat auch nicht funktioniert. Also, auf jeden Fall, wenn das so eine freie Sache ist und wo es klar ist, ich habe die Struktur da nicht vorgegeben, das ist natürlich da schon klar. Schule, das ist hier auch klar, also hier ist auch ganz klar, wenn es zu Auseinandersetzungen kommt, dann habe ich das Sagen. Also, das ist für die auch irgendwie klar. P2, und die sagen das auch: „jetzt kommt der Chef“. Das finde ich total bescheuert, andererseits hilft es mir natürlich auch, hier Strukturen vorzugeben. Weil, das ist natürlich auch so ein freies Feld. Aber trotzdem müssen hier natürlich auch Regeln eingehalten werden. Und da, ein gutes Beispiel, Name 8 war ja auch mal hier. Das ist ein Mann und Sozialpädagoge und hat versucht, was klarzumachen. Da haben die gesagt: „Du hast hier gar nichts zu sagen.“ Der hat da gesessen und gesagt: „Ich“? „Du hast hier nichts zu

melden." Dann sagte er: „Ich sage nichts mehr." Der ist raus und dann bin ich da hin „hier Leute". Dann war (-) Aber witzig. Weil ich ja auch nur eine Frau bin, da habe ich gedacht, ich hätte eigentlich nichts zu sagen. Aber das ist nicht so. Also da sind die Regeln oder die Strukturen auch irgendwo klar.

P1: In der Klasse, innerhalb des Klassenraumes (-). Ich meine, nach den Pausen gab es immer mal etwas zu klären, aber wenn der Unterricht lief, dann lief er auch.

P2: Und ich glaube, dass es keine Unterschiede gibt, zwischen den Flüchtlingen heute und den Flüchtlingen damals.

P1: Nein, das glaube ich auch nicht.

P2: Ne, kann ich mir nicht vorstellen. Da wird es, die Jungs, oder die Kinder, das ist ähnlich. Na klar, das ist ja nun auch eine Generation, eine andere Generation. Aber trotzdem sind die Schwierigkeiten die gleichen. Es hat sich auch nicht viel getan. Weil, die sind ja auch alle wieder zurück. Dann war das für uns ja auch wieder kein Thema mehr.

P1: Ja, aber noch einmal eben, es hängt natürlich auch von dem Bildungsstand der Eltern dieser Kinder ab, wie integriert, oder wie gut die dann mitarbeiten. Da gibt es auch große Unterschiede.

I: Gibt es möglicherweise auch Ressourcen in Klassen, die eben aus sehr vielen verschiedenen Kulturen bestehen?

P2: Naja, aus den Kindern selber. Wenn du merkst, dass Kinder bestimmt Stärken haben, die kannst du ja aufgreifen. Ich meine da gibt es ja nun tolle, also ich denke

immer, da gibt es ja tolle Konzepte, ob das Schlichtstreiter sind.

P1: Streitschlichter

P2: Streitschlichter, genau, oder wie sie alle heißen. Alles toll, aber das fängt ja schon bei den Kleinigkeiten an. Also, dass du Ressourcen erkennst, die aufgreifst und da drauf aufbaust. Das finde ich natürlich. Aber sonst, keine Ahnung, ob das in den Klassen (-)

P1: Du meinst jetzt, ob bei deutschen oder bei ausländischen Kindern, dass sie sich gegenseitig zum Beispiel helfen, das tun sie. Die sind da sehr vorbehaltlos, finde ich immer, die Kinder. Die nehmen sich so, wie sie sind und (-). Wobei ich jetzt hauptsächlich wieder die Mädchen meine. Bei den Jungen auch, die Mehrheit auch. Es gibt da immer so ein paar, nicht immer, aber es gibt dann doch Leute, die dann so rein (-). Mit ihren Aggressionen nicht so recht wissen wohin.

P2: Aber wenn du die richtig erwischt, das ist schon so.

P1: Selbst die Kinder von Name 9, wenn du die, nur für einen Moment sie (unv. Wort)

P2: Ja, aber das kann man ja aufbauen. Ich sehe das immer nicht so aussichtslos.

P1: (-) Die sind ja auch nicht böse eigentlich, die sind nur wild und aggressiv. Aggressiv ja nicht, aber wild und unerzogen. Aber sie sind nicht wirklich boshaft. Kann man nicht sagen, ne.

…

Transkription des Interviews mit External 2

Legende:

Interviewer=„I“; Proband=„P“

Pausen=(...)

unverständliches Wort=((unv.Wort))

Wort- und Satzabbrüche= (-)

- Anonymisierungen=[…]

I: Wunderbar. Gut. Die erste Frage ist, welche Erfahrung hast du im Umgang mit psychisch-belasteten Kindern?

P: Also, wenn es auf den Kontext Schule bezogen ist, dann hab ich die Erfahrung mit psychisch belasteten Kindern […] im Umgang mit Kindern, die im Bereich emotionaler, sozialer Entwicklung auffällig sind […] da ging es ganz viel darum, wie man die Strukturen, die drumherum sind, die die Familien überhaupt irgendwie anzapfen können, nutzen kann. Und im Rahmen dessen war ich auch in Schulklassen und hab hospitiert, hab mit Lehrern Austausch geführt, hab mir die Kinder angeguckt, hab einzeln Maßnahmen mit Kindern gemacht. […].

I: Ok.

P: also das war so der Großteil, den ich da gemacht habe.

I: Gut. Hast du Wissen und Kenntnisse darüber, wie sich psychische Belastungen von Kindern in der Klasse äußern? Also welche Symptome die zeigen und so?

P: Also, wenn ich über die Kinder nachdenke, bei denen wir wissen, dass sie tatsächlich psychisch-belastet waren, wenn da zum Beispiel Traumatisierungen vorlagen oder ähnliches, dann gibt’s da-, also dann ist die Bandbreite ziemlich groß das Symptom quasi, das was man sieht, reicht von Stören des Unterrichts, das ist ja die Standardannahme, ein Kind wird laut und und passt nicht in diesen Kontext. Viele Lehrer beschreiben das ja immer gerne mit „wie die über Tische und Bänke gehen.“, die halten uns da auf und das sind die Kinder, die wirklich die Probleme machen. Dann, was man aber auch sieht, sind Kinder, die sehr zurückgezogen sind,

die sich gar nicht in den Unterricht einbringen, wo viele Lehrer da vielleicht auch gar nicht den Blick dafür haben, dass da vielleicht auch eine Belastung vorliegt, in welcher Form auch immer. Und es kann aber auch-, also das sind halt die Auffälligkeiten, die reichen von-, also das ist quasi die Spannbreite, die man sieht, aber dazwischen ist halt alles möglich und es gibt auch Kinder, die psychisch belastet sind, die nicht auffallen. Von denen wir aber wissen, dass sie psychisch belastet sind, wo wir aber sehen„ Ok, die sind sehr gut in der Lage dazu, sich da zu integrieren und den Alltag zu bewerkstelligen.“ .

I: Wie kann sich denn diese psychische Belastung oder auch die Symptome, die die Kinder zeigen auf die anderen MitschülerInnen auswirken?

P: Ist auch ganz unterschiedlich. Also es gibt Schülerinnen und Schüler, die da ganz entspannt mit umgehen, die sagen „Ja, Gott, dann ist da halt einer, der ein bisschen-, der ist halt anders, aber anders sein ist vollkommen ok!“ und da gibt es auch Lehrer, die da gut mit umgehen können. Es steht und fällt halt häufig mit der Lehrkraft. Und die Schülerinnen und Schüler, die damit ganz gut umgehen können, haben sich da auch oftmals nicht von ihrem eigentlichem Ziel, was die ja oftmals haben, also so zielstrebige Schüler gibt es ja oftmals auch schon in der Grundschule, was ich sehr faszinierend finde, weil ich, also ich war das glaube ich nicht, die schon sehr den Fokus auf das Lernen setzen und dann sagen „ach, ich kann das doch Ausschalten, wenn da um mich herum was passiert.“ . Es gibt aber auch tatsächlich Schüler, die so laut sind und so viel Aufmerksamkeit auf sich ziehen, dass Unterricht nicht stattfinden kann. Und gerade wenn die Kombination von mehreren Schülern mit psychischen Belastungen in einer Klasse stattfindet, vielleicht auch gar nicht unbedingt psychische Belastung, das kann ja auch

sein, dass das einfach irgendwie Kinder sind, die aus einem schwierigem sozialen Umfeld kommen oder die, wo Eltern auch einfach sagen „Also die, also uns ist auch einfach gar nicht so wichtig, dass unser Kind die sechzig Minuten auf dem Platz sitzen kann.“. Das hat das nicht gelernt und ja, die dann in Kombination -. Da lassen sich Schüler dann auch gerne schnell ablenken. Das ist der Moment, wo Schüler laut werden, die psychisch belastet sind. Wenn Schüler leise sind, die beeinflussen andere Schüler wenig bis gar nicht. Also da gibt es dann natürlich auch diese ganz sozial veranlagten Schüler und Schülerinnen, die dann so „Ach, was hast du denn?“ und die kümmern sich dann gerne, aber ansonsten lenken die natürlich wenig ab und deshalb wird -, also mit eine der Thesen, die man ja immer so hat, wird der Fokus auch weniger auf die gelenkt, weil die Unterricht stattfinden lassen.

I: Ja. Welche Herausforderungen entstehen denn für Lehrkräfte in Klassen, in denen psychisch, oder psycho-sozial belastete Kinder sind?

P: Also ich glaube die größte Herausforderung ist, sich damit auseinander zu setzen, dass man dafür nicht ausgebildet wurde und festzustellen, dass man der Situation oftmals nicht gewachsen ist und einfach nicht weiß, was man tun soll. Darin sehe ich die größte Herausforderung, weil das ein riesiger Zwiespalt ist in den sie da stecken. Einerseits habe die irgendwann mal gelernt an einer Uni und in Ref., die müssen denen was beibringen, hier geht es um Lernen, die müssen Mathe, Deutsch und Englisch können und dann kommen da auf einmal Kinder, für die ist das gar nicht wichtig, für die ist gerade was ganz anderes wichtig. Für die ist wichtig, dass die in sozialen Kontakt treten und die haben auch nicht gelernt „Danke“ und „Bitte“ zu sagen und vorsichtig an die Schulter zu klopfen, sondern die hauen ein

Kind, weil das ihre Form der Kontaktaufnahme ist und damit können viele Lehrer nicht umgehen. Ich glaube, das ist ihre größte Herausforderung. Aber, also, dass sie damit nicht umgehen können und merken, dass sie es nicht können, ich glaube, dass der Schritt, der schwierigste an allen ist. Sich einzugestehen „ich brauche da Unterstützung“ und das das System, in dem wir, also das ist ja auch von Ort zu Ort unterschiedlich, aber bei uns an den Schulen, spezifisch in Stadt 1, aber auch im Landkreis, ist es so, dass die oftmals aber auch alleine gelassen werden. Man muss es dazu sagen. Also es gibt dann Schulen, die zum Beispiel ganz gut zum Beispiel mit „XXX“ ausgestattet. Dann haben wir irgendwie hier zwei Lehrer hier in Stadt 1, wenn die ganz, wenn die eine persönliche Beziehung haben zu den Kollegen, dann arbeiten die gut zusammen, dann haben die auch gutes Handwerkszeug in Anführungszeichen, dann gibt es Methoden, die denen vielleicht dabei helfen, damit umzugehen, auf die Kinder zuzugehen, die Kinder wahrzunehmen und zu sagen „Alles klar dann“ -, klassiches, klassischer Satz aus der sozialen Arbeitsstörung haben vor Rang. So lange diese Störung nicht weg ist, kommen wir auch nicht voran. Das geht halt einfach nicht. Ja, ich glaube, dass das die größte Herausforderung ist. Das zu akzeptieren, zu sagen, ja dann muss ich mich da jetzt drum kümmern und das hat dann Vorrang. Und das oftmals die Basis, die in Schulklassen geschaffen wird, um wirklich mit denen zu arbeiten nicht sicher genug ist. Es wird halt von vorne rein gesagt, es geht halt ums Lernen, aber eine gemeinsame Basis zu haben, um zu sagen, wir können auch gemeinsam Lernen, wir akzeptieren, dass wir an unterschiedlichen Punkten sind und jeder darf von seinem Punkt aus starten, weil haben zum Beispiel eine gemeinsame Vorstellung davon, wie wir miteinander umgehen oder ähnliches, ich glaube, dass die Basis oft nicht groß genug ist. Aber ich-, also ich glaube auch, dass die Vorwurfshaltung, die viele Leute haben, egal ob es Lehrkräften gegenüber ist, Integrationshelfern oder wem

auch immer, das das definitiv nicht der richtige Weg ist, weil keiner im Moment in der Situation ist, dass er an der Stelle, wo er ist, richtig ausgebildet ist, weil das -, der Prozess ist halt schwierig.

I: Glaub ich. Du hast das schon so ein bisschen angedeutet, was gibt es denn für Unterstützungsmöglichkeiten für Lehrende in so einer Situation, wenn sie belastete Kinder in der Klasse haben?

P: Also was es mittlerweile an Grundschulen fast überall gibt, sind neben pädagogischen Mitarbeitern auch tatsächlich auch Schulsozialarbeiter, die, da gab es vor anderthalb oder zwei Jahren nochmal eine große Offensive vom Land, dass an den Grundschulen fast überall Schulsozialarbeiter sind, gerade wenn es Ganztagsschulen sind, die natürlich unterstützen können. Dann haben Schulsozialarbeiter oftmals mittlerweile ein Team um sich herum gebaut mit den pädagogischen Mitarbeitern, vielleicht auch mit Leuten aus der Tagespflege, die den ganzen Tag mit unterstützen, dann Bundesfreiwilligendienstleistende, ganz viele Bundesfreiwilligendienstleistende, die sind überall. Das ist auch gut, dass die überall sind. Das hat oftmals den Effekt, dass die Lehrer, die Möglichkeit haben eine Situation so ein bisschen zu entlasten, weil sie zum Beispiel einzelne Schüler jemanden an die Hand geben können, die dann in diesem professionellem Kontext Schule aber auch eingebunden sind, die sind Teil der Schule. Dann gibt es externe Unterstützer, zum Beispiel Integrationshelfer, das ist jetzt seit diesem Schuljahr anders geregelt irgendwie. Da gibt es jetzt einen Schulhelferpool. Das war vorher anders. Es gibt ja einen gesetzlichen Anspruch von Eltern auf Integrationshilfe für das Kind. Diese Integrationshilfe müssen sie dann beantragen beim Amt für Kinder, Jungend und Familie oder beim Sozialamt, das kommt auf den

Förderbedarf dann des Kindes an. Lehrer haben immer gerne gesagt oder auch Schulleitung ganz groß „Gehen sie mal, holen sie mal den Integrationshelfer für ihr Kind, ihr Kind ist ganz anstrengend!“. Damit hängt aber auch zusammen, dass dieses Kind einen Stempel aufgedrückt bekommt, dass es von einer seelischen Behinderung bedroht ist oder seelisch behindert ist, erst dann gibt es ja einen Integrationshelfer nach Paragraph 35a, blablablabla. Das ist jetzt anders gelöst, es gibt jetzt eben diese ganzen Integrationshelfer in einem Schulhelferpool und da wird anhand von Fallzahlen an den Schulen entschieden, wie viele dieser Integrationshelfer an die Schulen gehen und die Schulen selber entscheiden, in welche Klasse jetzt gerade jemand gehen kann, um zu unterstützen. Das hat den Vorteil, dass es nicht einzelne Kindern sind, die diesen Stempel bekommen, die-, der organisatorische Aufwand im Vorfeld sehr viel geringer ist, der gesetzliche Anspruch der Eltern ist trotzdem noch da. Also wenn Eltern sagen-, also den kann man ja nicht einfach wegstreichen, das obliegt einfach niemanden hier in Stadt 1 zu sagen „den gesetzlichen Anspruch hast du nicht mehr.“ und die Schulen können das selber organisieren, das heißt, wenn ein Lehrer Unterstützung braucht, um auf die Ausgangsfrage zurückzukommen, kann er „Hier“ schreien und dann muss man innerhalb der Schule gucken, wie macht man das. Gibt es noch Stunden, dass dieser Integrationshelfer mit dazu kommen kann. Und dann ist es auch möglich zu sagen, ich habe hier zwei, drei Kinder, die in einem bestimmten Unterrichtsfach, weil die inhaltlich nicht so gut aufgestellt sind, weil die mit der Lehrkraft nicht so gut klarkommen oder so, mit diesem Menschen arbeiten und das ist nicht nur an ein Kind gebunden. Das wäre eine Möglichkeit. Weitere externe Hilfen wäre „XXX“. Da muss man, also es gibt ein Meldeverfahren, da kann man in der Schule sagen „ich fülle diesen Meldebogen aus“, der geht von der Klassenlehrkraft zur Schulleitung, von der Schulleitung zu den verantwortlichen Förderlehrkräften, die das Team mit

betreuen und dann wird überlegt, inwieweit man da weitermachen kann. Das ist ein sehr ausgereifter Prozess, das heißt es gibt auf jeden Fall Förderpläne, das ist immer damit verbunden diese Förderpläne werden mindestens alle zwei Monate, glaube ich, besprochen. In den Förderplänen sind konkrete Ziele festgesteckt, zur Mitarbeit der Lehrkräfte, der Eltern, der XXXfachkräfte. In einem XXXteam sind immer noch ein Sozialarbeiter mit dabei, der auch Einzelfallarbeit mit den einzelnen Kindern machen kann oder wenn man feststellt, bei so Hospitationsgängen oder so, dass man irgendwie sagt „ja, ok, das liegt vielleicht doch eher an so einem Klassenproblem“, könnte man auch sagen, die Sozialarbeiter von XXX, die machen mal so ein XXXtraining für die ganze Klasse um mehr Grund darein zu bringen. Und dann gibt es im nächsten Schritt noch die Möglichkeit, wenn es Kinder sind, die viel mehr Unterstützung haben müssen, Einzelunterstützung. Dann gibt es noch „Name-Kräfte“, die machen professionelle Einzelfallarbeit im Unterricht mit Schülern. Die sind, im Gegensatz zu Integrationshelfern, die ja nur so ein paar Wochen Kurs irgendwo an der Schule 1 machen hier, pädagogisch professionell ausgebildet, also das mindeste, was sie glaube ich mitbringen müssen ist eine abgeschlossene Erzieherausbildung. Aber ich glaube die-, ich bin mir unsicher, was die Kollegin, die jetzt hier in Stadt 1 unterwegs sind für eine Ausbildung haben, aber die sind professionell ausgebildet. Die können auch in einen Hilfeplan eingeteilt werden, die Zuteilung der „Name-Stunden“, das kann man sich wünschen als Lehrkraft oder als Schulleitung und sagen „Hallo, ich brauche die unbedingt, ich brauch jetzt mal professionelle pädagogische Hilfe an diesem Kind und zwar für so und so viele Stunden. […]. Ja, das ist das, was man von extern holen kann. Wenn es tatsächlich um so was wie ein Sozialtraining geht oder man so ganz konkrete Problemstellungen hat, könnte man auch immer mit uns in Kontakt treten. […] könnte man sagen, man überlegt sich eine Art von Hilfe, die man installiert in Form von

regelmäßigen Treffen mit diesen Kindern, über einen freien Träger. Das könnte man finanzieren über fallübergreifende Hilfen, die Option gibt es auch, das wissen aber die Wenigsten und das ist auch nichts, was irgendwo auf dem Papier steht, also wo man sagt „Oh, ich würde gehen.“, das ist nur so, wenn man irgendwie mal mit Leuten intensiver ins Gespräch kommt und wirklich gar nicht mehr weiß, was man machen soll, dass man das sagt „Ok, da könnte man auch nochmal einen Schritt weiter gehen.“. Also das sind die Sachen, die ich so aus meiner Arbeit kenne. Ach so, die Schulpsychologin, Landesschulpsychologin, die kann natürlich auch hinzugezogen werden, die ist aber natürlich total überbucht. Also ist sie seit Jahren! Ich kenne sie jetzt seit fünf Jahren und seitdem weiß ich, die hat eigentlich immer viel zu viel zu tun. Das geht aber auch! Ah, wir haben auch noch einen -, geht auch noch, einen Kinder und Jugendpsychiatrischen Dienst vom Gesundheitsamt. Das ist dann noch so eine Leistung vom Landkreis, die man anfragen könnte. Das ist aber noch nicht lange so. Also die haben wir jetzt erst seit, ich glaube, einen knappen Jahr und da geht es auch noch drum, wie die sich aufstellen. Aber da könnte man nochmal sagen, gerade wenn es um psychischen Hilfebedarf in irgendeiner Form geht und man das nicht richtig einschätzen kann, die können einem dann dabei helfen, so eine Einschätzung zu machen. Das wäre auch noch eine Option.

I: Was fehlt denn vielleicht noch, um Lehrkräfte oder Schulen zu unterstützen?

P: Also mal ganz -. Also frech gesagt, fehlt die Bereitschaft von Lehrkräften und Schulen sich auf die Situation einzulassen. Das ist das, womit wir tagtäglich zu tun haben. Schulen, Lehrkräfte, Schulleitungen, wie auch immer, rufen an und sagen uns „Es geht so nicht. Ihr müsst uns helfen.“.

Dass sie Gesetzeslage eine vollkommen andere ist und dass XXX da überhaupt gar nichts machen kann im Kontext Schule, das ist dann schon wieder ganz egal, aber die wissen dann, dass das XXX irgendetwas mit denen zu tun hat und dann meinen die, wir sind dann verantwortlich. Das ist natürlich nicht so, aber das muss man denen dann auch erzählen, wie so was läuft und zum Glück sind viele dann auch einsichtig in Anführungszeichen und sagen dann „Ok, das wusste ich nicht.“. Es gibt schon relativ viele Fortbildungsangebote, die man, also die auch uns bekannt sind, wo es um unterschiedliche Bereiche geht. Ich glaube, dass so was Flächendeckendes trotzdem sinnvoll wäre, um sich überhaupt mit dieser Fragestellung auseinanderzusetzen. „Was kann überhaupt psychische Belastung sein? Wo kommt das her?“ und dann geht es für mich immer wieder an den Punkt, dass man an dem Menschenbild arbeiten muss. Das ist immer das Ausschlaggebende. Man muss, wenn man mit allen Kindern arbeitet, es ist egal, ob die eine psychische Belastung haben oder nicht, muss man sich mit seiner eigenen Vorstellung von Menschenbild auseinandersetzen. Man muss sich reflektieren, man muss überlegen, was mache ich da überhaupt. Das fehlt Lehrkräften. Lehrkräfte sind oft dafür ausgebildet Kindern etwas beizubringen, die haben dann didaktisch vielleicht noch einiges auf dem Kasten, aber wenn es darum geht, wirklich in Problemsituationen pädagogisch sinnvoll zu handeln, stehen die ganz schnell an einem Punkt, wo sie nicht mehr weiterwissen. Das kann ich denen nicht verübeln, überhaupt nicht! Mach ich auch nicht, also das wäre, wer wäre ich, wenn ich mir sowas anmaßen würde, ich glaube, dass da ein ganz, ganz großer Haken ist und das können auch keine pädagogischen Fachkräfte, im Sinne von keine Lehrkräfte im Schulkontext auffangen. Das geht nicht. Da muss sich in meinen Augen, was in der Ausbildung ändern. Ganz stark. Und da hat, also Bundesland 1 hat das ja irgendwann mal versucht mit der Umstellung vom GHR-300 auf irgendwie, du studierst jetzt zehn Semester und du

hast ein Praxissemester im Bachelor und ein Praxissemester im Master mit der Idee, dass das wissenschaftlich begleitet wird, dass diese Fragestellungen aufgearbeitet werden können. Ich weiß nicht, ob das immer noch so ist. Das war so, als ich aufgehört hab zu studieren. Ich war bei der Ummodelung dieses Studiengangs irgendwie mit dabei bei uns und ja, das ist natürlich wünschenswert, dass auch dann die Fragestellung natürlich aus der Praxis mitgebracht werden an die Uni, die Uni auch versteht, was da überhaupt passiert und vielleicht so ein bisschen Schwung darein kommt. Aber da weiß ich nicht, ob das wirklich stattfindet. Ich habe letztens von einem Bekannten gehört, der Lehramt studiert, er darf keine Kurse und Vorlesungen zur Inklusion besuchen. Er wird Gymnasiallehrer. Das ist nicht vorgesehen. Ja. Also das ist so ein bisschen an der Inklusion vorbei, aber herzlichen Glückwunsch!

I: (ironisch) Klasse.

P: Ja, das dürfen die nicht, so viel Platz haben die leider nicht in den Seminaren. Ja, ich glaube da hakt es oft.

I: Ja! Ja. Jetzt noch ein paar Fragen zu interkulturellen Klassen. Hast du eine Idee, was für spezielle Herausforderungen entstehen durch, also für Lehrkräften durch interkulturelle Klassen? Hast du da -, kennst du dich da irgendwie mit aus?

P: Also das was an uns weitergetragen wird, ist das es schwierig ist, weil die Familien überhaupt gar keine Vorstellungen davon haben, wie unser Bildungssystem funktioniert, das heißt, bis sie überhaupt erstmal da ankommen, ist schon mal ein langer Weg und, also es gibt

hier eine relativ gute Betreuung der Familie, muss man sagen, also die verstehen dann relativ schnell „Ok, dass Kind muss hier in die Schule. Das muss da regelmäßig hin. Ach hoppala.“ und wenn sie sich dann damit abgefunden haben, bei vielen Familien läuft das dann und dann wissen die das Kind muss da immer morgens hin und das ist auch nicht so eine ‚Komm-wann-du-willst-Struktur’, da muss man echt morgens um acht sein, dann hat man sich irgendwann damit angefreundet. Viele Kinder finden da halt auch gefallen dran, muss man auch sagen, für die ist das natürlich auch cool, weil die dann da irgendwie andere Kinder haben und das ja für die ist das dann schon auch schön. Was ein riesiges Problem ist, ist die Sprache. Also das hören wir an allen Ecken und Enden, es ist einfach schwierig, gerade wenn die Kinder im Grundschulalter hier ankommen und dann in die Schule gehen und dann diesen Unterricht mitmachen sollen, dann haben Lehrer wieder zu wenig Zeit und sind -. Also, ich weiß nicht, wenn man hier so eine Umfragen machen würde oder wie viele Leute hier Deutsch als Zweitsprache bei uns im Studium gemacht haben, nicht viele! Also wenn ich an mein Studium denke, ich habe auch erst Lehramt studiert und dann umgesattelt auf Bildungswissenschaften und uns wurde im Deutsch-Studium, also uns wurde das als Wahlangelegenheit damals so angeboten. Ja, kann man machen. Kann man machen, ist jetzt aber zusätzliche Arbeit. Überlegt euch das gut, ob ihr das machen wollt. Das müsste halt eine Pflichtveranstaltung sein und auch früher hätte das schon sein müssen, das ist eigentlich eindeutig. Und dann hat man ja, das schöne ist, wenn man das gemacht hat, dann hat man ja auch nochmal so einen Teil interkultureller Pädagogik mit vielleicht auch so einer Vorstellung davon, was in anderen Kulturen überhaupt irgendwie passiert, wie Eltern mit Kindern umgehen, was Schule bedeutet, was Bildung bedeutet, welchen Stellenwert Familie hat und Ähnliches. Was die vielleicht mitbringen hierher und das es auch Zeit einfach in Anspruch nimmt, dass die sich mit anderen, mit einer anderen Kultur

auseinandersetzen. So, das ich glaube das ist ganz oft nicht so. Interkulturelle Pädagogik ist an Schulen so gut wie nicht vertreten, also ich wüsste nicht wo und das macht es natürlich zusätzlich schwierig. Also auf jeden Fall -, aber das größte Problem in den Klassen -, also unsere Klassenlehrer sind alle interkulturell. Ich weiß nicht was die offizielle Bezeichnung von einer interkulturellen Klasse ist, aber –

I: die meisten sind -

P: Genau. Also hier ist zumindest an den großen Schulen, also Schule XX und Schule XY, die haben alle interkulturelle Klassen und zwar auch ganz oft ziemlich dicke. Also da sitzen dann auch viele Kinder. Wobei ich bin selber auch in Schule XX zur Schule gegangen, du wahrscheinlich auch?

I: Ja!

P: Wir hatten auch damals schon immer solche Klassen.

I: Ja, ja genau.

P: Schülername 1 aus Land 1, dann kam jemand aus Land 2, also das, ja. Land 3 hatten wir auch noch! Das war immer ganz, ja. –

I: Ja. Ja.

P: Aber also Sprache und auch das Verständnis auch so, oder? Also die, ja, also was an Schulstruktur vielleicht auch

fehlt ist die Möglichkeit überhaupt in Kontakt zu treten mit Eltern und diese Nähe herzustellen. Das ja, vielleicht einfach die Beziehungsarbeit und so die ganzen, die besorgten Bürger sind halt auch Lehrer. So blöd das klingt, aber das ist, die haben halt Sorgen, ganz oft auch, dass sie irgendetwas falsch haben. Also die haben auch Angst, vor was auch immer! Also was, gerade Kinder, oder? Tschuldigung, was soll mir dieses Kind tun? Was soll mir ein Kind mit einem anderen kulturellen Hintergrund mehr tun als ein deutsches Kind? Bitte. Passiert nichts. Also weiß nicht, ja. (lacht)

I: (lacht) Ja. Welche Herausforderungen können denn für die Schüler in, also allen in den Klassen entstehen, dadurch, dass einfach Kinder aus verschiedenen Kulturen gemeinsam unterrichten werden?

P: Also die Schüler und Schülerinnen selber, ich glaube, die sehen da gar nicht so eine große Herausforderungen. Die werden eher übertragen von Lehrern und Eltern. Gerade, also es gibt da natürlich auch zum Glück auch viele Lehrer, die da sehr offen sind und die, die Problematik da nicht so sehen und sagen „Ja, komm, wir kriegen das schon irgendwie hin.“, die sich in dem Bereich auch zumindest irgendwie ein Buch in die Hand nehmen und sich überlegen, wie kann ich denn damit umgehen. Eltern sind da oftmals einfach ganz schwierige Multiplikatoren, die das an die Kinder weitertragen und da ist, also, dadurch wird der Umgang dann schwierig. Ich glaube die Kinder an sich sehen das nicht als Problem an. Ja, die, also die reden mit Händen und Füßen und die erklären mit draufzeigen, das ist mein Pausenbrot. Pausenbrot? Pausenbrot. Und dann ist gut. Gerade in den jüngeren Jahrgängen ist das unproblematisch und viele kommen ja, die kommen ja schon aus Institutionen. Welches Kind geht heute nicht in den

Kindergarten? So, also, ja. Bei mir war das beispielsweise so, ich war nicht im Kindergarten, aber wir waren halt immer so dreißig Kinder. Ich bin halt in Straße XY aufgewachsen und da waren wir immer so dreißig Kinder so drum herum und ja. Aber mittlerweile kommen die aus einer Institution, wo bereits interkulturell irgendwie interkulturell gearbeitet werden müsste, rein theoretisch, wo Kinder aus unterschiedlichen Nationen sind, die unterschiedlichen Background mitbringen und die kommen dann in die Schule und ich glaube, die Kinder sind nicht das Problem. Das was problematisch ist, ist eher das was an Lehrern getan wird.

I: Ja. Was gibt es denn vielleicht für Ressourcen in interkulturellen Klassen?

P: Naja ganz Viele! Genauso wie in nicht interkulturellen Klassen. Aber im Speziellen in interkulturellen Klassen, ja, wie definiert man denn die Ressourcen, die da da sind? Also was ich ganz faszinierend finde ist, dass man da sieht, dass diese Offenheit da ist und das aufeinander zugehen, dass das nochmal eine andere Form von sozialen Umgang miteinander ist und Hilfsbereitschaft, die da eine Rolle spielt, wenn ein Kind sieht, ein anderes Kind versteht irgendetwas nicht, dann ist die erste Intention des Kindes nicht zu sagen „Du hast dunklere Haut als ich mit dir rede ich nicht.“, sondern zu sagen „Ich helfe Dir!“ und diese Hilfsbereitschaft, die da drin steckt, die ist eine Ressource, die da sehr viel mehr sichtbar werden kann, wenn man dafür den Nährboden in Anführungszeichen hat. Aber es kann natürlich auch in eine ganz andere Richtung gehen. Also alles was man als, also was wir als positive Ressource sehen würden, kann man natürlich auch im negativen sehen. Also alles was Kinder mitbringen an Ausgrenzungspotential, kann da natürlich auch potenziert werden. (überlegt). Ja, also Sprache an sich sowieso. Also überhaupt auch zu

verstehen, es gibt unterschiedliche Sprache und andere Sprachen zu hören, diese Ressourcen, die da irgendwie ausgebaut werden. So eine Anlage, praktisches Interesse an Sprachen zu haben, die Möglichkeit, sich damit zu beschäftigen, ist natürlich auch nochmal ganz spannend. Und überhaupt ein Interesse an Fremden und Offenheit, so ich glaube das das, ja, so die Diversität bla, bla, bla, also alles das könnte man natürlich als Ressource sehen.

Kann aber auch immer in das Gegenteil umschlagen, wenn man da die Leute hat, die sich ganz viele Sorgen machen und so viel Angst mit sich herumtragen, weil ja so viele Dinge schiefgehen. (lacht)

…

Transkription des Interviews mit External 3

<u>Legende:</u>

Interviewer=„I“; Proband=„P“

Pausen=(...)

unverständliches Wort=((unv.Wort))

Wort- und Satzabbrüche= (-)

- Anonymisierungen=[…]

I: Die erste Frage ist, welche Erfahrung hast du mit psychisch belasteten Kindern?

P: Viele. Ich habe 7 Jahre in der Beratungsstelle für Opfer von Missbrauch gearbeitet während dem Studium und während des Referendariats. Das ist die Erfahrung Nr. 1 und wir haben die Kinder dort beraten und begleitet. Und dann natürlich in der Schule, also jeden Tag eigentlich.

Psychisch belastet durch Gewalt, sexuelle Gewalt, Flucht, Traumata, posttraumatische Belastungsstörung vermutlich auch. Drogenabhängigkeit der Eltern, volles Programm.

I: Und wie äußern sich oder wie können sich diese Belastungen bei den Kindern in der Klasse äußern?

P: Das geht von, ich mach jetzt mal so ein Beispiel mit 'nem Klischee, was aber meistens oft bestätigt wird, von Mädchen, die ganz still in der Klasse sitzen bis über Kinder, die nur mit anderen Kindern sprechen, also so Mutismus, halt spezifischer, bis hin zu ich hau meinen Mitschülern aus dem Nichts oder vermeintlich aus dem Nichts, eine Reißzwecke ins Gesicht und mach noch mal die Hand hin und her oder ich werfe, weil ich den Unterrichtsraum verlassen muss, weil ich zum Beispiel Stühle geworfen habe, also der Junge jetzt, von draußen den Stuhl durch die Glasscheibe, schnapp mir die Schere und geh damit auf andere Kinder los bis hin zu, zwei Jungs haben letzte Woche den Gruppenraum komplett zerlegt. Also vorletzte Woche, in der Woche vor den Ferien. Sie haben irgendwie alles aus den Schränken ausgeräumt, nur weil eine Vertretungslehrerin da war, die sie nicht kannten, für eine Stunde. Ja. Das ist so die Bandbreite.

I: Wie oft kommt sowas vor?

P: Jeden Tag. Also mindestens einmal. Aber ich bin auch in vier verschiedenen Klassen.

I: Was kann das denn für einen Einfluss auf die anderen Schüler in der Klasse haben?

P: Es gibt Kinder, die haben Angst vor dem Verhalten, ganz klar, haben (-) erzählen das zum Beispiel auch zuhause dann den Eltern, die finden das meistens dann nicht so toll und kommen dann an und fragen, was die Schule gedenkt dagegen zu tun, wir haben zum Glück immer eine Antwort drauf und meistens schon was gemacht. Kann aber auch sein, dass die anderen Kinder denken: „Oh, der kann das machen, ich mach mal mit, mal sehen, was passiert.“.

I: Was stellt es denn für Herausforderungen an die Lehrenden in solchen Klassen?

P: Das geht von, also, ich kenne mehrere Kollegen, die jetzt schon irgendwie 'ne längere Pause machen mussten, weil sie es nicht mehr ausgehalten haben. Eine Kollegin, mit der ich z.B. seit vier Jahren zusammen arbeite, die immer jetzt Klassen hatte, die mindestens 7 Kinder mit emotional-sozialem Förderbedarf, das heißt psychisch belastet auf jeden Fall, in der Klasse hatte, das ist für die jeden Tagen ne Herausforderung und an manchen Tagen kann sie es besser wuppen und wenn man dann aber mal über einen längeren Zeitraum das Gefühl hat, man kann gar nichts mehr machen und die Kinder sind verloren, dann geht das ganz schön an die Substanz.

I: Das glaube ich.

P: Einige werden dann häufig krank. Ja.

I: Gibt es denn irgendwelchen Möglichkeiten, die Lehrende dabei unterstützen im Umgang mit psychisch belasteten Kindern?

P: Ja, eigentlich hat jede Schule 'nen Beratungsdienst, wo sich auch die Kollegen Unterstützung holen können bzw. wo die Beratungslehrer weitervermitteln können an Institutionen. Dann gibt es kollegiale Freiberatung, die wir auch im Beratungsteam anbieten, wo eben dann auch umfänglich geguckt wird, also ganzheitlich, was braucht das Kind, was braucht die Familie und was braucht die Klasse und die Kollegin, um weiter damit umgehen zu können.

I: Gibt es noch irgendwas, was fehlt oder was noch gebraucht würde?

P: Ja, von der Behörde finanzierte Supervision in manchen Fällen. Das merkt man ganz klar, weil Beratungslehrer, Sonderpädagogen und Sozialpädagogen, die im Beratungsteam sind und 'ne Fallberatung machen, sind halt keine Supervisoren oder Psychologen. Das ist 'ne andere Sache.

I: Welche speziellen Herausforderungen entstehen denn durch interkulturelle Klassen bzw. durch Klassen, in denen

eben Kinder verschiedener Herkunft gemeinsam unterrichtet werden?

P: Ganz oft fängt das an bei sprachlichen Schwierigkeiten, weil dann die Kommunikation nicht funktioniert und ein Kind, was sich nicht ausdrücken kann durch Sprache, versucht dann beispielsweise durch in Körperkontakt gehen, in Kontakt zu anderen zu kommen. Wenn aber ein Kind dabei ist, was körperliche Gewalt erfahren hat, kann es damit wieder nicht umgehen und dann entwickelt sich so eine Dynamik, allein aufgrund dessen weil die nicht sprechen können untereinander. Und dann kommt eben noch dazu, manche Kinder die 'ne Flucht miterlebt haben, haben posttraumatische Belastungsstörungen oder zumindest Symptome davon, können sich vor allem oft ganz schwer konzentrieren, kriegen dann durch die sprachlichen Schwierigkeiten nicht die Inhalte im Unterricht mit und dadurch entsteht auch so ein Kreislauf aus Frustration und dann wieder nach außen agieren.

I: Gibt es da auch spezielle Herausforderungen für die Lehrkräfte in solchen Klassen?

P: Ja klar, wir haben z.B. in einer Klasse zwei Kinder, die kommen aus Land 1, sprechen minimal Sprache 1, weil sie zur Region 1 Minderheit in Land 1 gehören oder gehört haben, die sind jetzt hier, leben in massiv beengten Verhältnissen, das wissen wir, zwei Familien mit ungefähr 12 Leuten in zwei Zimmern. Und die sprechen beide kein Deutsch, sind jetzt in der 1. Klasse. Der eine wiederholt die 1. Klasse gerade, hat auch in dem Jahr wenig gelernt, was Sprachliches jetzt angeht. Wir haben erstmal nur sprachlich unterstützt und für die Lehrerin ist das wahnsinnig schwierig, allein auf der Ebene von Lernen, da Material zu erstellen,

weil theoretisch können die Kinder nur in 1:1 Betreuung gefördert werden oder vielleicht 2:1 und das kann man selbst mit 20 Kindern in der Klasse nicht leisten.

I: Entstehen dann auch irgendwelche speziellen Herausforderungen für die Schüler und Schülerinnen?

P: Die anderen?

I: Ja.

P: Ja klar, wenn z.B. 'ne Kollegin was (-) oder ein Kollege irgendwelche Inhalte immer wieder erklären muss, weil es auf der sprachlichen Ebene nicht funktioniert, ist es für die, die es eben schon verstanden haben, langweilig. Dann fängt der eine an mit dem Stuhl zu kippeln, dann fängt der nächste an Sachen zu werfen. Das ist oft schwer auszuhalten oder auch dann einfach für die anderen Kinder wenn man im Sportunterricht ein Spiel erklärt und also so auf der sozialen Ebene, wir wollen spielen oder Fußball spielen und der hat die Regeln nicht verstanden, dann werden die irgendwann sauer. Also das geht oft, wenn man Kollegen hat, die viel investieren und sich da mit viel Geduld 'ran wagen, geht es ne Weile gut, aber irgendwann sind die anderen total abgenervt.

I: Gibt es denn auch Ressourcen in interkulturellen Klassen vielleicht?

P: Ganz viele. Also (…) das fängt schon allein an auf wie viel Sprachen wir immer 'Happy Birthday' singen können. Ist wirklich so, weil wir immer jede Sprache, die in der Klasse

gesprochen wird, so über die Grundschulzeit hinweg dazulernen mit allen zusammen. Aber auch so Dinge, wie wenn ein neues Kind kommt, das beispielsweise eine Sprache noch nicht kann, gibt es auf jeden Fall oft ein oder zwei Kinder, die dann beim Übersetzen helfen können. Also und auch die unterschiedlichen Kulturen und die unterschiedlichen Blickwinkel sind auf jeden Fall 'ne Ressource.

I: Wie äußert sich das mit den verschiedenen Kulturen? Merkt man das im Unterricht?

P: Ja, teilweise auch auf 'ne Art, die ich persönlich nicht so schön finde, z.B. wenn dann Kinder mit Land 2 Migrationshintergrund sagen, Klasse 1: „Nee, das ist ein „Land 3“ Papa hat gesagt, ich darf nicht neben dem sitzen.“.

I: In dem Alter schon?

P: Ja. Oder, „Iihh, du isst Schweinefleisch. Ich darf deinen Tisch nicht mehr berühren“. Klar. Oder wenn man mit, (...) wir waren damals mit der Klasse auf einem Ausflug und dann ist ein Mädchen zu einer wildfremden Frau, die hatte einen ganz kurzen Rock an, hingegangen und hat die total niedergemacht und beleidigt, weil das Papa nicht möchte, dass Frauen solche Sachen tragen. (…) Es äußert sich aber auch im Positiven, weil man z.B. auch, wenn wir so Erzählkreise vom Wochenende machen, sowohl die Kinder dann von religiösen Feiertagen erzählen und man dann ganz viele Dinge mit aufnehmen kann. Und auch da ist es ja dann wieder 'ne Ressource, das viele Kinder ganz unterschiedliche Bräuchen und Rituale zu Hause erlebe und nicht irgendwie dass es 'ne Mehrheit gibt, die was Ähnliches

erlebt und wenige, die was anderes haben. Ist schon spannend.

…

Transkription des Interviews mit External 4

Legende:

Interviewer=„I“; Proband=„P“

Pausen=(...)

unverständliches Wort=((unv.Wort))

Wort- und Satzabbrüche= (-)

- Anonymisierungen=[…]

I: Dann nochmal. Ok! Die erste Frage ist, welche Erfahrungen Sie haben im Umgang mit psychischen belasteten Kindern?

P: […] das heißt die Erfahrung kommt dann aus dem Beratungskontext, jetzt konkret […], dass ich dort eben mit psychisch belasteten Kindern beispielsweise Testungen durchführe, Intelligenztestungen mache oder sie im Unterricht beobachte, wenn es bei mir jetzt auch konkret um Leistungsprobleme in diesem Zusammenhang geht und vorher halt in der Arbeit an der Grundschule mit dem gesamtem Spektrum der psychischen Belastungen, die Schüler mitbringen können.

I: Das ist schon ein ganz gutes Stichwort. Haben Sie Kenntnisse darüber, wie sich denn psychische Belastungen bei Kindern in der Klasse ausdrücken können?

P: Ja! Soll ich also auf die konkreten Kinder mal eingehen?

I: Ja.

P: Ja, das ist -, ich denke so ein ganz ganz wichtiger Punkt ist die Konzentrationsfähigkeit und vor allen Dingen aber auch der Umgang mit allem was irgendwie -, was stört. Also die Kinder sind oft, ich sag mal sehr dünnhäutig in vielerlei Hinsicht, dass sie sehr schnell und stark reagieren können auf Sachen, die auf sie einprasseln. Dass sie eben auch, ja, nur wenig Möglichkeit haben mit Konflikten umzugehen, also, das ist häufig so, dass belastete Kinder da eben sehr schnell auch so ausagierendes Verhalten zeigen können wenn Sachen, ich sag mal abweichen von dem was-, ja vom Alltag, also sei es, dass jemand mit ihnen in Streit gerät oder

dass eben aber auch eine Veränderung im Alltag ist. Ein anderer Lehrer oder eine Absage von irgendetwas worauf sich das Kind gefreut hatte, also all diese Sachen, reagieren die Kinder deutlich empfindlicher drauf und was man jetzt gerade bei Geflüchteten beobachten kann ist auch, dass viele Kinder sich halt stark zurückziehen können. Also das zum einem eben Kinder sind, die sehr ausagierendes Verhalten haben, aber auf der anderen Seite eben auch Kinder sind mit Belastung, die sich sehr zurückziehen, sehr still sind. Was zum Beispiel auch schon vorgekommen ist, tatsächlich auch mit mutistischen Verhaltensweisen, also dass sie nicht gesprochen haben oder nur mit bestimmten Personen gesprochen haben. Ja. Erstmal so.

I: Ja. Welchen Einfluss können denn diese Ausdrucksformen auf die anderen Schüler und Schülerinnen der Klasse haben?

P: Ja, also, zum einem eben auch eine gewisse -, also was oft ein Problem ist, was man als Lehrer manchmal versteht, warum ein Kind in bestimmten Situation auf eine bestimmte Art reagiert, dass kann ein Mitschüler nicht und das ist zum Beispiel eine Sache, dass die Kinder beispielsweise mit Belastungen in die Schule kommen und dann getriggert werden, dann kommt es zu einem Konflikt und das andere Kind kann halt zum Beispiel mit dieser heftigen Reaktion nicht umgehen und dann steigert sich das eigentlich noch. Oder aber dass Kinder eben auch ausgegrenzt werden, weil sie in ihrem Verhalten, ja, ich sag mal anders sind, schräger sind, anders reagieren. Also sie finden dann auch schlechter Spielkameraden. Je nach psychischer Belastung kann das sehr sehr unterschiedlich sein. Also auch ein Kind, was sich zurückzieht, das wird dann irgendwann auch nicht mehr gefragt, ob es mitmachen kann oder ein Kind, was sich komisch verhält, das wird tatsächlich dann auch oft eben

nicht mit einbezogen in Spielsituationen, würde ich sagen. Und, was natürlich auch ist, ist, dass je nach dem wie stark die Kinder das auch zum Ausdruck bringen, kann natürlich auch eine ganze Lernsituation belastet sein, also weil-, weil halt die Konflikte und auch die Probleme dann in den Pausen ausgetragen werden dann in den Klassenverband reinkommen, so. Ja.

I: Ok. Welche Herausforderungen entstehen denn für die Lehrkräfte in Klassen, in denen psychisch belastete Kinder sind?

P: Also ich glaube die größte Herausforderung ist tatsächlich, das alles richtig einzuordnen. Ich denke das psychische-, psychisch belastete Kinder sehr sehr stark darunter noch zusätzlich leiden, dass wir in Schulen sehr Symptom-, sehr viel an den Symptomen arbeiten und die auch bemerkt sind. Also dass natürlich zum Beispiel ein Fehlverhalten als solches interpretiert wird und als solches gemaßregelt wird ohne auf die Gründe zu gucken, warum das Kind jetzt auf diese Emotion sich falsch verhält. Also das ist, glaube ich, die wichtigste Aufgabe für die Pädagogen, das Verhalten des Kindes im Zusammenhang verstehen zu lernen, das Kind dann auch kennen zu lernen und auch einzuordnen. Also gerade jetzt auch bei Traumatisierung ist es eben ganz wichtig, dass man weiß, dass wenn das Kind zum Beispiel getriggert ist und in einer Situation, dann ja ausflippt oder auch nicht mehr irgendwie zu bändigen ist, dass es dann beispielsweise nichts bringt in so einer Situation mit Druck zu arbeiten und dann noch zusätzlich da irgendwie da noch verstärkend drauf einzuwirken, sondern dass man das halt einordnen kann und weiß warum das Kind so ist in der Situation und das ist schwierig und das ist eben dann auch schwierig den anderen Kindern zu erklären, dass es also auch zum Beispiel -. Das bestimmte -, also das

Verhaltensweisen auch unterschiedlich bewertbar sein müssen dann, in solchen Situationen. Wenn man jetzt ein stark psychisch belastetes Kind in der Klasse hat, muss man vielleicht manchmal auch Dinge aushalten, die man bei anderen Kindern vielleicht dann nicht -, also dass kann ja auch eine Signalwirkung sein für andere sich genauso zu verhalten, sondern das müssen dann irgendwie auch alle Beteiligten auch lernen, dass es da auch unterschiedliche Maßstäbe manchmal geben kann und dass es für ein Kind eine unglaubliche Leistung ist, mal eine Viertelstunde am Platz zu sitzen und für die meisten Kinder ist das eine Selbstverständlichkeit. Aber das dann zum Beispiel anerkennen, wenn ein Kind das schafft, das ist dann halt auch wichtig.

I: Welche Möglichkeiten gibt es denn um Lehrende zu unterstützen im Unterricht mit psychisch belasteten Kindern?

P: Ja, also das Wichtigste, finde ich, ist erstmal Wissen, also das Fortbildungsangebote, dass es da Aufklärung darüber gibt, das auch die Möglichkeiten, das zum Beispiel Schulpsychologen beratend hinzugezogen werden, dass das genutzt wird und das es auch die Möglichkeiten eben gibt oder die Kapazitäten dafür. Ich denke auch, dass es auch Raum geben muss eben für Supervision, für Austausch um überhaupt das los zu werden. Auch so die Probleme, die man vielleicht hat. Es ist zum Beispiel auch unglaublich wichtig, dass man bestimmte Verhaltensweisen des Kindes nicht auf sich bezieht. Also da tun sich auch einige Lehrer durchaus mit schwer, dass sie denken so – Wenn das Kind zum Beispiel seine Wut über die Situation die Eltern auf die Lehrer projiziert und dann loslässt, dass man halt auch irgendwie von sich abgrenzen kann und da braucht man, denke ich, in manchen Dingen eine Unterstützung und Backup. Und es ist auch wichtig, dass eben die Schule

selbst mit einbezogen wird. Also das auch Leitung und auch das andere Schulpersonal auch bereit ist, wenn der Lehrer sich zum Beispiel auf die Schüler einstellt und sich da fortbildet, dass die anderen ihn auch unterstützen und da auch -, also da auch empfänglich für sind. Also zum Beispiel ein Hauptproblem ist, man hat das Kind in der Klasse und es funktioniert, aber das Kind in der Pause bei der Aufsicht einer anderen Person funktioniert nicht und dann passiert was und dann-, es kommt der Lehrer, der andere Lehrer, der Aufsicht hat, wütend zu dem Lehrer hin und sagt „Ja dein Name XY hat wieder die ganzen anderen Kinder geschlagen“ oder sonst was ist passiert oder hat irgendetwas gemacht was nicht geht, sondern dass die anderen das auch im Grunde das einzuordnen wissen und auch dann - , ja, vielleicht dann auch an den Klassenlehrer weniger vorwurfsvoll rangehen, sondern eher stützend. Also ich glaube, da ist-, kann man die Klassenlehrer viel unterstützen. Oft ist es ja so, dass die Kinder ja tatsächlich beim Klassenlehrer deutlich besser funktionieren und dass weil viel über Beziehung geht und gerade Lehrer, die da sehr offen sind und das auch können, zu den Kindern wirklich eine gute Beziehung aufbauen können, aber dass das halt in einer Vertretungssituation oder in anderen dann ganz schwierig sein kann.

I: Klappt das denn in den Schulen, dass das unterstützt wird oder dass man sich gegenseitig unterstützt?

P: Das ist sehr unterschiedlich! Also das kann man, glaube ich, überhaupt nicht pauschal sagen. Also es gibt, glaube ich, Schulen, die sind da super gut und haben auch eine sehr gute Kommunikation untereinander und es gibt auch Schulen, wo die Lehrer sehr einzelkämpferisch arbeiten und so ihr Ding durchziehen. Also das ist wahnsinnig unterschiedlich. Und auch was zum Beispiel die

Fortbildungen zum Thema psychische Erkrankungen angeht, sind die Schulen sehr unterschiedlich aufgestellt. Also manche nehmen das jetzt gerade im Zusammenhang mit der Flüchtlingskrise auch zum Anlass sich in Hinblick auf traumapädagogische Sachen auch fortzubilden und da steckt ja viel drin, was eben auch auf andere psychische Erkrankungen zutrifft. So, und ich hab selbst für mich auch gemerkt, dass die Arbeit, die ich hier […] habe, die hätte mir auch sehr geholfen in der Arbeit an der Grundschule vorher, weil auch viele Kinder, die hier aufgewachsen sind einfach hohe Belastungen haben und auch-, ja bis hin zur Posttraumatischen Belastung. Ja.

I: Ok. Jetzt geht es zu den interkulturellen Klassen. Welche speziellen Herausforderungen bestehen denn in interkulturellen Klassen?

P: Viele. Ja, also da -, ja es fängt zum Beispiel auch, wenn man da jetzt auch an psychische Probleme denkt, fängt es ja damit an, dass man das voneinander-, ja einzuordnen muss. Also was ist jetzt zum Beispiel eine interkulturelle Besonderheit, was ist eine persönliche Besonderheit, was hängt vielleicht mit Traumatisierung zusammen, mit psychischen Belastungen. Und auch in der Ansprache mit den Eltern ist es halt wichtig, dass man da auch als Lehrer zum Beispiel drüber bescheid weiß, wie das in den Herkunftsländern verstanden wird. Also man kann nicht einfach zum Beispiel von psychischen Problemen sprechen oder von „Gehen Sie mal mit ihrem Kind zu einer, zu einem Psychologen.“, weil das bei vielen Eltern einfach Ängste auslöst und eine starke Abwehrhaltung, weil sie was ganz anderes damit verbinden! Und das heißt es ist erstmal zum Beispiel bei den-, der erste Punkt ist, die Kinder oder die Jugendlichen sich untereinander richtig verstehen und das einzuordnen, aber auch, dass die Lehrer auch mit den

Kindern und auch mit den Eltern von den Kindern auch nicht das gleiche voraussetzen können, wie bei zum Beispiel bei Hier-Aufgewachsenen. Also das man eine andere Ansprache braucht oder auch eine andere Beratung, sozusagen. Ich weiß jetzt nicht, ob das so in die richtige Richtung geht?

I: Ja! Die ist ja recht offen die Frage.

P: Genau. Und generell kann man tatsächlich erleben, dass Kinder, also gerade je jünger sie sind, desto weniger spielen eigentlich kulturelle Dinge eine Rolle, so also bei Grundschülern ist es im Grunde all das, was wir als interkulturelle Probleme haben kommt von, eher von Eltern und auch eher von Lehrern, Erwachsenen, von unseren Erwartungshaltungen. Je jünger die Kinder sind, desto schneller finden die eigentlich zueinander und haben keine Berührungsängste und ja je mehr sie dann aber auch ihre, manchmal auch ihr kulturelle Rolle annehmen, je älter sie werden, kommt es dann eben auch zum Beispiel zwischen den Kindern und Jugendlichen zu Barrieren, also -. Und das ist -. Also ich habe vorher an einer Schule gearbeitet mit einem sehr hohen Anteil an Kindern mit Migrationshintergrund. Das war eine Grundschule und in 90% aller Klassen hat Religion zum Beispiel nie eine Rolle gespielt. In einer Klasse schon und das war mal ganz interessant zu beobachten. Das eine sehr religiöse Lehrerin, also sehr Religion 1-religiöse Lehrerin und ich glaube, das das so über die Jahre bei einigen Schülern zu so einer Verstärkung ihre eigenen Identität oder ihrer eigenen religiösen Identität hervorgerufen haben, die bei den anderen Klassen überhaupt nicht vorhanden war und ja, das ist-, das fand ich ganz spannend.

External 4

I: Ja. Sie hatten das schon so ein bisschen angesprochen, gibt es denn Herausforderungen, die entstehen für Schüler und Schülerinnen in interkulturellen Klassen?

P: Also eine Sache ist auf jeden Fall, was wir immer im sonderpädagogischen als Sprachhandlungskompetenz bezeichnen. Also das natürlich Schüler mit geringeren Kenntnissen in der deutschen Sprache, haben weniger Möglichkeiten ihre Gefühlswelt eigentlich auch auszudrücken und das ist vor allen Dingen bei Konflikten führt es dann, wenn diese Sprachhandlungskompetenzen fehlen dazu, dass eben auf andere Möglichkeiten zurückgegriffen wird, um den Konflikt zu lösen und das ist dann halt eher dann mit körperlichen Konflikten. Also das auch eben Streitigkeiten sehr schnell aufgrund von Missverständnissen entstehen. So ein ganz beliebtes Ding ist zum Beispiel das zwei Kinder sich prügeln und man kommt dazu und fragt „Was ist passiert?“ und beide Kindern sagen dann halt „Der andere hat angefangen!“ und meistens ist es so, dass der Erste den anderen ausversehen geschubst, getreten oder sonst was hat, dass das keine Absicht war, sondern das passiert. Der andere hat aber nicht nachgefragt, sondern hat zurückgetreten und beide waren jetzt fest der Meinung der andere hätte angefangen. Der Erste hat ja gar nicht gemerkt, dass es ausversehen passiert ist und der Zweite hat gedacht „Der hat mich einfach so getreten.“ und dass die Schüler beispielsweise nicht die Frage stellen „Warum hast du mich getreten?“. Also das hinterfragen sie nicht, sondern wenn mich einer tritt, trete ich zurück. Und das wird ja manchmal auch durchaus von Eltern durchaus so propagiert „In der Schule, du musst dich wehren und wenn die anderen gemein zu dir sind, dann hau ruhig mal zurück!“, so. Und das passiert natürlich schneller, wenn Sprache, als Kommunikationsmittel fehlt und bei Kindern, die eben auch sehr starken Belastungen ausgesetzt waren, ist es auch so das eben-, zum Teil eben auch Handlungsmuster sind, die

sie am eigenen Leib erfahren haben. Also Kinder, die zu Hause geschlagen werden oder die auch Gewalt in der Familie erleben, die kennen das natürlich auch eher, dass man so Konflikte löst. Und dann muss man da auch langsam-, also das muss man dann im Grunde auch erst mit denen erarbeiten, wie das auch auf anderer Ebene geht, weil Kinder lernen auch von ihren Eltern und logischerweise oder von dem, was sie erleben und das denke ich, ist bei geflüchteten Kindern auch das Problem, dass -, oder kann ein Problem sein, wenn sie selbst viel Gewalt erlebt haben, dass sie natürlich dann auch in Krisensituationen eher dazu neigen das auch selbst dann zu nutzen, weil sie es nicht anders gelernt haben. Und ja, das ist eben einer der wichtigen Punkte, wo man als -, wo man eben ansetzen kann eben über Sprache und auch da denen das wirklich beizubringen und das muss dann halt tatsächlich auch im Unterricht verankern und kann das nicht einfach irgendwie nur als Reaktion auf Konflikte machen, sondern muss das halt wirklich etablieren. Und da ist es eben auch was man erlebt, dass je nach kultureller Herkunft, die Eltern ja auch selbst nicht die Erfahrung haben, wie sie zum Beispiel erzieherisch tätig sein können. Also im afrikanischen Raum ist es durchaus üblicher, dass körperlich zum Beispiel bestraft wird. Und das sagen die Eltern auch und sie sagen auch oft in den Gesprächen, dass sie wissen, dass sie das hier nicht machen dürfen und daran sich auch halten, aber sie zeigen dann auch die Hilflosigkeit, dass sie eigentlich keine Alternative haben. Sie wissen nicht, wie man Kinder anders erzieht. Also die Eltern und das haben wir-, bemerke ich öfter in den Gesprächen fehlt so ein bisschen, ja, auch so erzieherische Kompetenzen so wie sie hier bei uns ich sag mal so ein bisschen gelebt werden, weil sie das eben so aus ihren Herkunftsländern so in der Form nicht kennen. Das war eben eine andere-, das ist ja auch eine andere Familienrolle, -situation auch eine andere Familienhierarchie als hier. Und all das lässt sich nicht so einfach übertragen und das findet dann natürlich auch in den Klassen statt, so in der

Elternarbeit, aber auch in dem, was die Kinder von zu Hause gewöhnt sind und dann steht das auch manchmal im Wiederspruch, was die Eltern sagen, was der Lehrer sagt und all diese Dinge. Das sind also schon vielschichte Probleme so, die entstehen können und ja, das ist schon recht komplex, dass immer zu lesen.

I: Ja! Ja. Gibt es denn eventuell auch Ressourcen in interkulturelle Klassen?

P: Ja, natürlich! Also man lernt ja durch Vielseitigkeit. Also die Kinder lernen schon auch über den Tellerrand zu gucken und auch, dass man mit Verschiedenheit umgehen kann und sich auch arrangieren, dass man auch Gemeinsamkeiten findet und das ist auf jeden Fall ein großer Schatz, den man da nimmt und auch wenn zum Beispiel Kinder von ihren Ländern, von ihren Ritualen, von ihren Sitten, Gebräuchen erzählen, dann ist das eine große Bereicherung und auch Sprache kann sehr bereichernd sein, wenn man sich das anguckt und man kann daran auch zum Beispiel viel lernen. Also das-, ich finde schon, das hat viele Vorteile und es hat auf der anderen Seite auch eben Vorteile, dass man, auch wenn man belastete Kinder eben in die Klasse integriert und wenn man als Lehrer darauf eingeht, kommt das anderen Kindern auch zu Gute. Wir haben hier viele, viele Kinder, die große Probleme haben und das ist, das steht halt nicht immer mit der Kultur zusammen und ich finde gerade, dass wir jetzt, also so wie zum Beispiel das Thema Traumatisierung im Vordergrund steht, zeigt halt doch, dass man auch durch die vielen geflüchteten Menschen, die bis nach Deutschland gekommen sind, auch sich Gedanken macht, so „Was können wir vielleicht noch besser machen, als zum Beispiel in den 90er Jahren oder früher.“.

…

Transkription des Interviews mit External 5

<u>Legende:</u>

Interviewer=„I“; Proband=„P“

Pausen=(...)

unverständliches Wort=((unv.Wort))

Wort- und Satzabbrüche= (-)

- Anonymisierungen=[…]

I: Meine erste Frage ist auch direkt welche Erfahrung Sie im Umgang mit psychisch belasteten Kindern haben?

P: Ich bin ja viele Jahre als Kinderärztin tätig gewesen und die Eltern kamen meistens mit den psychischen Problemen ihrer Kinder erst zu mir, das heißt zum niedergelassenen Kinderarzt, bevor sie eine der Fachkräfte, also Kinder- und Jugendpsychiater, aufsuchten.

I: Und haben Sie Kenntnisse darüber, wie sich psychische Belastungen bei Kindern in der Schule und in der Klasse ausdrücken können, also welche Symptome da gezeigt werden?

P: Ja also die Symptome sind sehr vielfältig und werden nicht immer gleich gedeutet als Ausdruck von psychischer Belastung. Die Kinder und das nimmt in den letzten Jahren in der Kinderarztpraxis sehr zu, leiden unter Kopfschmerzen, sie leiden unter Bauchschmerzen ohne organischen Befund und dieses habe ich sehr viel erlebt. Die Kinder leiden unter Schlafstörungen, sie leiden unter Appetitmangel, also unter Symptomen, die man primär erstmal einer organischen Erkrankung zuordnet und erst nach Ausschluss einer organischen Erkrankung darüber dann mit den Eltern spricht, welche möglichen anderen Ursachen in Frage kommen, wobei man, wenn man Erfahrung hat, häufig schon aus der Anamnese Hinweise dafür bekommt, dass es eine nicht organische Genese hat. So, das ist die eine Seite, also organische Befunde von psychischen Problemen. Dann haben wir Kinder mit Schlafstörungen, die nicht einschlafen können oder nicht durchschlafen können. Dann haben wir Kinder, wo die Eltern von Erziehungsschwierigkeiten berichten, also klassisches Beispiel 'Mein Kind hört nicht', wo die Kinder mit anderen Kindern nicht so gut zurechtkommen,

entweder mehr schlagen oder sich sehr zurückziehen, wobei dann immer, man gut erfragen muss, was könnte die Ursache dafür sein. Ja, das sind so die Sachen, die mir jetzt so schnell einfallen, also vielleicht können wir das erstmal soweit (-)

I: Die nächsten beiden Fragen beziehen sich jetzt darauf, welchen Einfluss diese Symptome auch auf die Mitschüler und die Lehrenden haben können. Haben Sie da irgendwelche Erfahrungswerte?

P: Ja also besonders im Vordergrund stehen natürlich immer die Kinder, die laut sind, also die viel Lärm machen. Wobei immer ja die Frage ist, wie weit ist Lärm nicht normal bei Kindern, also das muss man immer sehr gut gucken, wer jetzt eigentlich der Auffällige ist, das Kind oder die Umgebung. Also das finde ich eine sehr wichtige Frage. Also die mehr externalisiert sich verhaltenden Kinder, die lärmen, die nicht hören, die sich nicht sozial verhalten können, die keine Rücksicht nehmen können, das ist die eine Seite. Die verletzend wirken sowohl psychisch als auch körperlich. Und dann die andere Gruppe von Kindern, die sich vermehrt zurückziehen, die nicht in den Kontakt gehen können, die sich weder mit anderen Kindern in Kontakt begeben noch mit dem Erwachsenen in Kontakt begeben, die schwer aus der so genannten Reserve zu locken sind.

I: Gibt es denn Möglichkeiten, die Lehrende dabei unterstützen mit diesen Kindern umzugehen? Haben Sie da also zum Beispiel als Ärztin (-) also dass man da in Austausch treten kann?

P: Also wir haben keine institutionalisierte Form zu diesen Themen gefunden. Ich habe dann häufig einzelfallbezogene Gespräche mit den Lehrern angeboten und bei Kindern, die in Schulen mit besonderem Förderbedarf waren, habe ich von meiner Kinderarztpraxis auch den Eltern angeboten, dass wir ein gemeinsames so genanntes Fallgespräch mit allen Betreuenden gemacht haben und das war immer von sehr guter Wirkung, weil dann jeder seinen Blick auf das Kind darlegen konnte und wir da häufig zu guten Sichtweisen und Maßnahmebeschlüssen gekommen sind. Also ich habe aber nur einzelfallbezogene Unterstützung sowohl der Kinder als auch der betreuenden Personen organisiert, keine Programme oder so entwickelt.

I: Aber das haben Sie angeboten oder sind da Lehrer auch an Sie herangetreten?

P: Also das war, ich habe in Ort XY in der Praxis gearbeitet, das war von mir so eine Spezialität, sage ich jetzt mal. Weil ich hatte als Spezialgebiet auch XXX und da war das häufig auch ein Thema.

I: Gibt es denn irgendwas, was Sie meinen, was Lehrende da unterstützen könnte, jetzt neben so Einzelfallgesprächen, was Sie für sinnvoll empfinden würden in Bezug auf Kinder mit psychischen Belastungen?

P: (…) Na ja, auf jeden Fall Fortbildungen in der Frage, wie reagieren Kinder auf welche Belastung. Also dass man z.B. wenn ein Kind jetzt sehr viel lärmt oder nicht hört, dass man dann immer gut die Frage stellt, was ist der Grund dafür. Also dass man die Lehrer befähigt, diesen Weg zu gehen, einerseits die Grenzen zu setzen und andererseits aber das

Verständnis für das Kind aufzubringen, was natürlich in den großen Gruppen häufig schwierig ist, aber was glaube ich essentiell ist, um zum Verständnis für diese Kinder zu kommen und was da meines Erachtens nach sehr wichtig ist, dass die Lehrenden immer wieder in Einzelkontakt mit dem Kind gehen und häufig dann nicht in dem Sinne also die Kinder jetzt isolieren von der Klasse im Sinne von einer Strafe, sondern sie manchmal herausnehmen aus der Gruppe im Sinne einer zusätzlichen Zuwendung, also dass ist (-) meiner Erfahrung nach ist die, das eigentlich durch den Kontakt zum Kind, das allerwichtigste an Verbesserung seiner Situation geschaffen werden kann, durch den persönlichen Kontakt zum Kind. Was natürlich in einer großen Gruppe, in einer Klasse immer sehr, sehr schwierig ist.

I: Was gibt es denn für Besonderheiten bei den Kindern mit Flucht- oder Migrationshintergrund in Bezug auf psychische Belastung oder psychische Störung?

P: Ich habe wenig Erfahrung mit interkulturellen Klassen, muss man einfach sagen und ich glaube, dass es im Klassenverband sehr schwierig ist, die Situationen, die die Kinder erlebt haben, nachzuempfinden. Aber ich glaube, dass es sehr wichtig ist (-) ja es kommt natürlich sehr auf das Alter jetzt an. Ich weiß nicht auf welches Alter Sie sich (-)

I: Vor allem Grundschule, beziehe ich mich drauf mit der Intervention.

P: (…) Ja, also einmal ist, also ich würde sagen das Verstehen sowohl auf sprachlichem Niveau, als auch das

Verstehen kultureller Besonderheiten (-) also das halte ich für sehr essentiell im Umgang mit diesen Kindern. Dass sie die Möglichkeit bekommen auch zu zeigen, wie anders in ihren Kulturen bestimmte Situationen gemanagt werden. Das finde ich als einen sehr, sehr wichtigen Punkt. Dass man sozusagen unsere Normalität nicht immer als gegeben für diese Kinder voraussetzt.

I: Auch in Bezug jetzt auf psychische Störungen oder ganz allgemein?

P: Allgemein. Das meine ich jetzt allgemein, ja.

I: Wie ist das jetzt bei psychischen Belastungen? Sieht man da Unterschiede zwischen Kindern mit Fluchthintergrund und deutschen Kindern oder kommen dann doch ähnliche Symptome eigentlich?

P: Das finde ich interessant. Also nach meiner Erfahrung kommen doch sehr ähnliche Symptome zustande und vor allem so ein bisschen auch geschlechtsbezogen, dass die Jungen eher diese hyperaktiven Verhaltensweisen zeigen im Grundschulalter. Das ist eigentlich was internationales, ja.

I: Welche Ressourcen bringen denn die Kinder mit, mit Migrationshintergrund und Fluchthintergrund?

P: Das ist so differenziert. Ich kann da gar nicht sagen, dass ich sage (-) Ich finde das sehr, sehr interessant, wie unterschiedlich Kinder mit Migrationshintergrund aus ihren Erfahrungen Konsequenzen ziehen und (…) es ist im

Wesentlichen sehr abhängig vom Erziehungsstil und dem Verhalten der Eltern gegenüber den Kindern. Also wenn Sie jetzt z.B. Eltern haben, die wir ja heute sehr oft erleben, die ein sehr gewährendes Verhalten den Kindern gegenüber an den Tag legen, wenig Grenzziehung, sehr viel immer begründend, was Sache ist, wenn sie so ein Verhalten haben, dann und das haben Sie international bei vielen ja, finde ich nach meinen Erfahrungen (-) und aber bei vielen Kindern mit Migrationshintergrund ist es auch so, dass die Eltern einfach manche Verhaltensweisen fordern ohne große Begründung und ohne es lange zu diskutieren, also keine Ahnung, wenn man jetzt sagt: „Du bleibst jetzt hier, du gehst jetzt nicht raus“, dann bleibt das Kind hier. Und also insofern kann ich jetzt nicht sagen, also die Kinder aus der Kultur verhalten sich so oder haben solche Verhaltensauffälligkeiten und die Kinder aus einer anderen Kultur anders sondern meine Erfahrung, wobei ich das nicht theoretisch untermauern kann, ich weiß nicht ob das so stimmt, das ist jetzt einfach meine Erfahrung, dass es sehr abhängig ist vom Verhalten der Eltern gegenüber ihren Kindern und was ich viel erlebt habe, gerade türkischen Jungen gegenüber ist also ein geschlechtsspezifisches Verhalten, dass also von Jungen viel, also sagen wir mal das wir heute so Machoverhalten nennen, akzeptiert wird. Das finde ich schon teilweise auch schwierig, wo ich dann auch Gesprächsbedarf einfach erlebt habe.

I: Die sind dann aber auch bei Ihnen in der Praxis deswegen oder wegen anderen Dingen.

P: Die sind dann oft wegen anderen Sachen (-) also da finde ich dann manchmal Sachen auffällig, die die Eltern aber nicht auffällig finden.

…

Transkription des Interviews mit External 6

Legende:

Interviewer=„I“; Proband=„P“

Pausen=(...)

unverständliches Wort=((unv.Wort))

Wort- und Satzabbrüche= (-)

- Anonymisierungen=[…]

I: Die erste Frage ist ganz allgemein, welche Erfahrungen sie im Umgang mit Kindern mit psychischen Belastungen haben.

P: Mit diversen psychologischen Belastungen. Also unspezifisch ob Migration oder nicht?

I: Genau, einfach ganz allgemein.

P: Welche Erfahrungen ich damit habe? Also, welcher berufliche Hintergrund? Also, wir nehmen ja hier in der psychiatrischen Klinik jedes Jahr, weiß ich gar nicht, wir haben immer parallel acht bis zehn Patienten, aus verschiedensten Kulturkreisen, aber eben auch aus deutschen Familien mit Tikstörungen, Störungen des Sozialverhaltens, emotionale Störungen, Zwangsstörungen, also das große, nicht das vollständige, aber ein großes Spektrum von psychischen Erkrankungen landen bei uns bis zum Alter von 14 Jahren. Schulverweigerer und so was alles. Und da bin ich seit XXX hier in dieser Klinik und hab deswegen mit meinen vier bis fünf Patienten pro, für die ich immer zuständig bin, einen ziemlich guten Überblick über dieses ganze Themenfeld. Wenn das die Frage war, dann kann ich das so umreißen, ja.

I: Haben Sie Kenntnisse darüber, wie sich die verschiedenen psychischen Belastungen der Kinder in der Schule oder in der Klasse ausdrücken können?

P: Ja. Also, die Schulklasse ist ja mit zum Teil ein problemerzeugendes Feld. Wenn Kinder irgendwelche Besonderheiten mit sich hertragen, weil sie vielleicht ein bisschen ein kleines Handicap haben in einem Bereich, dass sie irgendwie nicht so gut mitkommen, oder dass sie von zu

Hause aus vielleicht ein bisschen anders sprechen oder eine andere Kultur haben, wie auch immer, dann kann es, dann wird ein Kind mit einer irgendwie geringfügigen Auffälligkeit schnell auch zum Gegenstand besonderer Beachtung bei Schülern. Und die können dann zum Teil auch abfällig reagieren, zum Teil ausgrenzen, zum Teil irritiert, verständnislos. Das wiederum wirkt sich dann verstärkend auf die psychische Problematik der Kinder aus. Das wiederum erzeugt wiederum verstärkte Symptome. Wir haben da häufiger mal einen Teufelskreis, je mehr sich Kinder denken, oder vielleicht erfahren haben, dass sie gemieden oder gemobbt, im Extremfall gemobbt werden, ziehen sie sich manchmal mehr zurück, vermeiden das mit, das darüber sprechen, beziehen höchstens noch ihre Eltern ein, die dann wiederum zum Teil dysfunktional darauf reagieren, indem sie sich mit dem Kind massiv verbünden, vor das Kind stellen, oder gegen vermeintliche Feinde angehen, oder indem sie das Kind selber wiederum ausschimpfen, warum es sich vielleicht so blöd verhält. Also die komplexesten Wechselwirkungen haben wir zwischen schulischem Feld und dem, wie es sich auf die psychische Störung auswirkt. Zum Teil ist natürlich durch die Platzierung von eingeschränkten, kognitiv eingeschränkten Kindern in diesem ganzen Inklusionsfeld zum Teil auch eine Überforderung bei Kindern der Fall. Und die Inklusion hat nicht nur Vorteile, sondern hat auch, nicht selten, den Nachteil, dass Kinder chronisch sozusagen in einer Vergleichssituation Unterlegenheitserfahrungen machen oder auch Überlegenheitserfahrungen. Und dass ist nicht immer so, wie das dann in den Zeitungen steht, dass quasi die Großen dann immer gleich die Kleinen auffangen und denen irgendwie helfen, sondern es kann sich auf Dauer auch ganz schön kränkend anfühlen. Und deswegen ist die Schule manchmal, beziehungsweise das soziale Miteinander in Klassen bei Leistungs- und Fähigkeitsunterschieden manchmal ein selbst ein verursachendes Feld für psychische

Erkrankungen im Sinne einer emotionalen Störung mit Schulvermeidung bis hin zu Schulverweigerung.

I: Wie kann sich das noch zeigen, diese Belastungen? Was für Symptome sind da in der Schule zu erkennen?

P: Wie das in der Schule dann als Symptom bemerkbar ist?

I: Genau.

P: Wir haben ja ein sehr allgemeines, wir haben ja keine spezifischen Störungen. Wir kennen Kinder, die sich kaspernd, ablenkend, störend verhalten, um von ihrem Problem abzulenken. Wir kennen Kinder, die Anforderungen verweigern, die quasi provokant gegen an gehen um eine vermeintlich gefühlte Schwäche zu überspielen. Wir kennen Kinder, die einfach still werden, zurückziehen, die sich gar nicht mehr äußern, die die Schule als solches gar nicht mehr besuchen wollen, die zu Hause Bauchschmerzen, Körpersymptome kriegen oder in der Schule enorme, meinetwegen bei Schulvermeidern dann auch Magenschmerzen, solche Empfindungen entwickeln, die dann in der Schule als irgendwie körperlich beeinträchtigt imponieren. Und die dann total verunsichern, was machen wir denn jetzt mit einem Kind mit Bauchschmerzen? Also, das sind so verschiedenste Bereiche. Es gibt natürlich auch psychische, also im weiteren Sinne psychische Störungen, Tikstörungen, Zwangsstörungen, die dann in der Schule irritierend und auch ärgerlich auftreten. Die durch laute Geräusche und so weiter die ganze Klasse durcheinander bringen. Und wiederum die Kommentierung der Mitschüler und Lehrkräfte wiederum auf das Kind zurückwirken. Also,

Schule ist für uns eigentlich ein Feld mit verursachendes und nicht nur mit erleidendes soziales Umfeld des Patienten.

I: So ein bisschen haben Sie es ja schon angedeutet, was können denn diese Ausdrucksformen der psychischen Belastung für Auswirkungen auf die Mitschüler oder die Lehrenden haben? Welche Herausforderungen entstehen da?

P: Ja also, Lehrkräfte sind nach meiner Erfahrung entweder unsicher bis ratlos, fühlen sich dann manchmal nicht kompetent, oder glauben, es übersteigt ihre Fähigkeit, es zu verändern oder damit gut umzugehen. Sie sind zum Teil auf Grund der Überforderung oder auf Grund der Tatsache, dass bestimmte Problemverhaltensweisen so störend oder den Unterrichtsablauf beeinträchtigen auch gereizt, verärgert, mitunter auch in ihren Kommentierungen wertend für die Klasse und haben ja öfter mal das Erleben, dass sie dem Kind, dem einzelnen Kind nicht gerecht werden können. Oder den anderen, wenn sie sich dem Kind wieder zu sehr widmen. Also dieser Spagat, der durch Inklusion und andere Herausforderungen ja sowieso nicht ganz einfach ist, der wird natürlich durch psychische Störungen nicht ganz einfach. Also wir haben manchmal große Mühe, den Lehrkräften quasi diese Anstrengung aufzubürden, darum zu werben, sich quasi doch noch mal verstärkt einem einzelnen Patienten wieder zuzuwenden, damit er rauskommt aus seiner Vermeidung und seinen Problemschleifen. In der Hoffnung, dass sich auf Dauer das auch ökonomisch rechnet, also vom Zeitaufwand.

I: Haben Sie Ideen, wie den Lehrkräften da geholfen werden könnte?

P: Also, wir machen ja oft so runde Tische, sogenannte runde Tische, wo wir uns mit den Lehrkräften, Therapeut, Lehrkräfte, Elternteil zusammensetzen. Manchmal auch ärztlichen Fachleuten dazu, um die Symptomatik, die dahinterstehenden Schwierigkeiten abzugleichen und damit die Lehrkräfte das besser durchschauen, was ist dahinter. Und damit wir dann quasi praktische Ideen ableiten, was kann der Einzelne beitragen, damit sie sich nicht überfordert fühlen, sondern mit ein paar greifbaren Vorschlägen ausgerüstet fühlen, ohne jetzt zu viel Bürde aufgebürdet zu bekommen. Wir versuchen aber auch, dieses häufig vorzufindende „Schuldige suchen“ Spiel zu unterbrechen, weil das gibt nicht selten Lehrkräfte, die denken, das Kind hat ein Problem, die Eltern sind schuld. Und das erzeugt eine enorme Feindseligkeit und Schutzreaktion bei Eltern, die dann auch mit den Lehrkräften nicht mehr kooperieren wollen, weil sie immer denken, sie müssen ihr Kind irgendwie vernünftig in die Schule schicken. Was sie aber nicht, einfach nicht können. Und das wäre ein zweiter Ansatzpunkt, nicht nur Information und Aufklärung, praktische Ideen, sondern auch so etwas wie Beziehungsverbesserung durch gegenseitiges Verständigen zu moderieren. Das ist ein zweiter wichtiger Baustein, was Eltern, glaube ich, den Lehrkräften auch hilft. Im Sinne vor der Beziehung zu den Eltern. Wir haben nicht selten Eltern, die mit den Lehrern quasi verkracht sind und da nicht mehr gut kooperieren. Das ist für die Lehrkräfte auch belastend. Vielleicht, schwierig sind auch noch die gruppendynamischen Prozesse, die unter Kindern ablaufen. Die Lehrer haben ja, es gibt ja eine hohe Dunkelziffer von unterschwelligen gegenseitigen Sticheleien und Bewertungen. Das haben die Lehrer oft gar nicht im Blick. Und da weiß ich nicht so genau, was den Lehrern am Ende wirklich hilft, außer dass man ihnen sagt, sie müssen eigentlich noch besser sein. Vielleicht braucht es da besondere Aufsichtsstrukturen, besondere, so auch grad im Pausenbereich, da haben sie dann das geringste. Im

Pausenbereich auch so was wie präsente Personensysteme, damit da dann keine dysfunktionalen Prozesse ablaufen, quasi. Eine andere Art von Freizeitgestaltungs-, Anregungsmilieu. Kräfte, die dort positiv wirken. Um so typische Effekte unter Kindern, auch ein bisschen sie zu begrenzen, die mit psychischer Erkrankung erstmal allein auch nicht was zu tun haben. Ja, das fällt mir so ein.

I: Gibt es spezielle Herausforderungen, die bei Kindern mit Migrations- oder Fluchthintergrund entstehen?

P: Kinder mit Fluchthintergrund haben, soweit ich das hier gesehen habe, zum Teil durch ihre Persönlichkeitsstruktur zum Teil sind manchmal durchaus attraktiv für andere Kinder. Wir hatten so einen schwarzafrikanischen Jungen hier vor einiger Zeit, der war überaus beliebt und attraktiv, der hatte einfach eine Kreativität, der konnte auch durch seine Art zu reden jede Menge Freunde gewinnen innerhalb kürzester Zeit. Es gibt andere, bei denen ist das Gegenteil der Fall. Es hat also sehr viel was mit der Persönlichkeit zu tun. Und natürlich spielt die Sprache da eine durchaus bedeutsame Rolle, die Sprachkompetenz. Was aber nicht so ganz einfach ist, ist für Eltern mit Migrationshintergrund, dass sie oftmals die Konflikte, die zwischen ihren Kindern und den Kindern deutscher Herkunft ablaufen, die durchaus natürlich sein können, häufig deuten mit der Brille der Opfer. Mit der Opferbrille, also mit der Wahrnehmung, wir sind ja sowieso ausgegrenzt und die haben bestimmt nur was gegen mich, weil (-). Das passiert auch mitunter Eltern manchmal hier. Das Konflikte entstehen, weil man meint, Mensch, die sollten vielleicht jetzt mal mehr dies machen, oder mehr beim Kind sein, wie auch immer, schnell eingeschnappt reagieren, weil sie sich quasi (-). Ist ja auch kulturell geprägt hier zu Lande, den Ausländern, den Migranten, den Leuten mit Migrationshintergrund, denen wird

ja nicht genug geholfen, und das erzeugt manchmal eine Opferperspektive, die bewirkt, dass sie sich schnell auch so als Opfer gemacht fühlen und dann aber auch in die Defensive gehen und nicht mehr in die Klärung. Sich dann zurückziehen, vorwurfsvoll, eingeschnappt reagieren. Das macht es dann schlimmer. Also das haben wir nicht selten. Solche Fälle, da muss man gut gegensteuern, damit das nicht passiert. Ja, ansonsten spielen natürlich manchmal auch religiöse Geschichten eine irgendwie verunsichernde Rolle, aber das ist hier in meinem Umfeld selten das Problem. Ob da jemand noch mal beten muss oder irgendwie eine andere Essgewohnheit hat, das ist kein Konfliktthema. So was sind persönliche Symptomatiken, die manchmal dann nochmal 'ne verstärkte Migrationsbrille bekommen, in dem Sinne, wie ich es eben erwähnt habe.

I: Unterscheiden sich denn die Symptome der Kinder?

P: Zwischen denen mit Leuten mit Migrationshintergrund?

I: Genau

P: Also, ich habe da keine Statistik drüber, ob wir bei mir Patient mit Migrationshintergrund (-), andere psychische Störungen sehen. Das fällt mir schwer, darauf zu antworten. Wir haben durchaus krasse Fälle, zum Teil auch mit neurologischen, oder auch Behinderungen dazu. Wir haben ja auch Kinder mit Behinderungen und psychischen Störungen, die zum Teil in ihren Herkunftsländern auch nicht richtig behandelt worden sind, wo da keine Behandlungsangebote sind, wo Eltern auch auf Grund ihrer Kultur wenig Vorwissen haben, zum Teil mehr Scham entwickeln, über das, was ihre Kinder haben, mehr

Vermeiden, zum Teil aber auch ihre Kinder zu wenig wiederum anleiten. Die sich so ein bisschen mehr verschmelzen. Also die öffentliche Auseinandersetzung, das Bewusstsein, der Familien, der Eltern mit Kindern mit psychischen (-), das ist gehäuft eingeschränkt, je nachdem, wie lange sie schon da sind. Weil sie sich nicht so lange haben damit auseinandersetzen können. Und deswegen ist es manchmal schwerer, Eltern mit Migrationshintergrund so zu beraten, dass sie den Standards, also, dass sie quasi einen neuen Blick auf die Kinder richten, weil sie noch einen weiten Weg haben, bewusstseinsmäßig zum Teil noch aus dem Mittelalter kommen, mal so als Bild gesprochen. Das ist so ein Punkt. Fällt mir noch was ein? Rollenvorstellungen spielen da natürlich auch noch 'ne Rolle, nicht. Also, das Rolle (-). Wir haben ja als Profis zum Teil Probleme, dass die Familien dann, manchmal dann den Kontakt mit männlichen Therapeuten verweigern, weil die Frauen dürfen dann nicht mit jemandem zusammensitzen, der männlich ist. Wenn da niemand weiteres zusammensitzt. Also, hierarchische, auch binnenfamiliäre Strukturen verhindern auch manchmal Veränderungen, wenn die eigentlich zuständige Mutter bestimmt Dinge nicht darf. Das sind tendenziell gehäuftere Phänomene als bei nicht Migrationsleuten. Einfach durch den historischen Hintergrund. Aber, dass es hier weniger Symptome gibt, oder mehr als bei Migranten, Migrationskindern, das wüsste ich nicht. Ob die jetzt mehr gemobbt werden, oder nicht, oder mehr emotionale Störungen (-). Das ist wahrscheinlich, dass das passiert, aber ich kann darüber keine Statistik sagen. Oder andere (-). Ich glaube, dass es hier und da Familien gibt, die aus fernen Ländern kommen, die gezielt auch Deutschland aufsuchen, weil sie hier mehr bessere medizinische Hilfe erhoffen. Und deswegen, weil sie dort sozusagen, so ziemlich allein gelassen sind, dass das manchmal sogar ein Migrationsanlass ist, weil hier mehr Versorgung für das Kind da ist, also für schwerbehinderte Menschen. Aber das ist

jetzt nicht die reine psychische Behinderung sondern die Kombi.

…

Transkription des Interviews mit External 7

<u>Legende:</u>

Interviewer=„I"; Proband=„P"

Pausen=(...)

unverständliches Wort=((unv.Wort))

Wort- und Satzabbrüche= (-)

- Anonymisierungen=[…]

I: Die erste Frage ist erstmal ganz allgemein, welche Erfahrung sie im Umgang mit psychisch belasteten Kindern haben.

P: Also, ich habe ja Psychologie studiert. […]. Soweit habe ich Erfahrung mit Kindern und Jugendlichen […]. Und da war ja meine direkte Arbeit mit Kindern und Jugendlichen mit seelisch-psychischen Problemen.

I: Haben Sie Kenntnis darüber, wie sich psychische Belastungen bei Kindern in der Schule und in der Klasse ausdrücken können?

P: Fast alle Kinder, die ich in so vielen Jahren getroffen habe, die Kinder, die zu Hause auffällig waren, die waren auch 99% in der Schule auffällig. Weil, die zeigen sich irgendwie, die Probleme, die zeigen sich überall, zu Hause, unterwegs, in der Gesellschaft. Und erst in der Schule, auch im Kindergarten. Und die Auffälligkeiten waren erst Desinteresse für die schulischen Pflichten. Das, was mir auch aufgefallen ist in so vielen Jahren, dass die Kinder überhaupt keine Lust auf Schule haben. Und die nehmen das ja nicht ernst, die wollen zu Hause bleiben. Oder die gehen zur Schule, aber die sind so weit auffällig, dass sie einfach irgendwie alles machen, was sie wollen. Und die halten sich ja nicht an die Regeln, weder zu Hause noch in der Schule. Und da merkt man, "oh irgendwas ist ja los". Wie gesagt, Desinteresse an schulischen Pflichten. Das ist für mich immer bei Kindern irgendwie der Punkt, wo ich sage „O.k., irgendetwas ist hier los mit dem Kind."

I: Wie kann sich denn dieses Desinteresse in der Schule zeigen?

P: Ja, wie gesagt. Das, ich nenne das Pflichten, aber, das die Schule überhaupt (-). Die anderen Kinder, die gehen gerne zur Schule, die lernen gerne. Die kommen einfach, die Probleme beim Lernen. Und überhaupt diese kleine Gesellschaft in der Schule irgendwie lösen. Auch teilweise nicht lösen, aber damit zurechtkommen. Und besonders sich an die Regeln halten, die Lehrer ernst nehmen. Das ist alles, was mir zeigt, ein Kind kann einfach verstehen, wo er ist und was er macht und welche Pflichten, in welchem Rahmen irgendwie er oder sie ist. Aber die Kinder, die einfach Auffälligkeiten haben und seelisch, psychisch nicht so stabil sind, die können ja nicht sich in diesem Rahmen halten. Die nehmen Unterricht ja nicht ernst, die lernen ja nicht, die wollen überhaupt nicht, die zeigen, dass sie überhaupt kein Interesse haben. Wie gesagt, die haben schlechte Noten, entweder keine Freunde oder Auffälligkeiten. Verhalten ist nicht in Ordnung gegenüber anderen Kindern. So zeigen sie, dass sie irgendwie kein Interesse überhaupt haben an der Schule.

I: Kann sich dieses Verhalten, was die zeigen, auch auf die anderen Schüler irgendwie auswirken? Also, hat das irgendwie einen Einfluss auf die Klasse oder die Mitschüler?

P: Ja, normaler Weise diese Kinder sind alleine, keiner möchte überhaupt diese Kinder als Freund nehmen. Und ich hab auch gehört, die Klasse möchte das Kind nicht bei sich, weil das Kind entweder laut ist oder geht auf andere verbal oder auch körperlich los, oder macht geschmacklose Witze, oder stört irgendwie beim Unterricht, manchmal wirklich außergewöhnliche Wirkung auf jeden Fall.

I: Was für Herausforderungen stellt das denn an die Lehrenden, so ein Kind in der Klasse zu haben?

P: Manchmal sind Gespräche mit Eltern. Ich habe mit Lehrern, wirklich ich habe ja Mitleid. Manchmal fragt man, warum sind die Lehrer so streng. Für mich, die sollen einfach wirklich streng bleiben, sonst die verlieren einfach irgendwie diesen Rahmen, was überhaupt drinne passt und was nicht. Manchmal ist das auch nicht gut, weil dann gibt es keinen freien Raum, das man einfach sagt, o.k. diese Kinder, die passen auch dazu. Herausforderung ist das wirklich für die Lehrer. Weil, die sollen irgendwie alles zusammenkriegen. Und manchmal schaffen die und manchmal schaffen die nicht, die Lehrer. In Gesprächen mit Lehrern, die haben gesagt, wir versuchen. Aber irgendwie, das Kind passt überhaupt nicht zu unserer Gruppe. Aber, wir können das Kind ja auch nicht irgendwie rausschmeißen. Wir schleppen also das Kind mit und alle Seiten merken das. Die anderen Kinder merken das, das Kind selber merkt das, dass es einfach irgendwie mitgeschleppt wird. Es ist egal, ob du da bist oder nicht. Und das ist ja auf jeden Fall irgendwie sehr, sehr schlechte Atmosphäre immer. Manche erfahrene Lehrer, die können auch nicht alles irgendwie schaffen. Kommt darauf an, was für eine Art von Art und Weise überhaupt die Lehrer haben. Für mich, die männlichen Lehrer, die haben mehr Glück.

I: Ach tatsächlich?

P: Ja.

I: Inwiefern?

External 7

P: Ich weiß es ja nicht, also, die Art und Weise von männlichen Lehrern, das ist einfacher, nicht so kompliziert, ruhiger. Hat Perspektive, was die überhaupt als Idee reinbringen, die sind locker und die sind nicht streng. Das kommt so vor, dass sie überhaupt nicht streng sind. Für Kinder immer männliche Lehrer, die sind angenehmer.

I: Das ist ja interessant, das wusste ich auch nicht.

P: Genau, die sind angenehmer, besonders für die ausländischen. Ich weiß es auch nicht, warum, aber das ist auch meine Erfahrung. Wenn man mit einem Lehrer überhaupt ein Gespräch führt, am Ende man kommt nicht mit leeren Händen raus. Oder man kommt nicht irgendwie auf 180, aggressiv, ohne Antwort raus. Aber das ist mir manchmal passiert mit Lehrerinnen, dass man, irgendwie kommt man raus und fragt „Und jetzt? Was sollen wir jetzt machen?“ Aber mit männlichen Lehrern nicht. Das ist auch meine Erfahrung. Ich weiß nicht, warum, aber die sehen die Welt total anders. Bei uns Frauen, das ist ja so wirklich. Und die Kinder, die bei mir sind, (-). […] lade ich einfach die Lehrer hier zum Therapiegespräch her. Weil, mein Punkt, wir sollen für unsere Probleme eine Lösung finden. Glauben sie ja nicht, von zehn Einladungen kommen einfach alle männlichen Lehrer 100%ig, wirklich. Sagen wir 8 und 2 kommen nur von den weiblichen. Die weiblichen, entweder die haben keine Zeit privat, die machen also da nichts, oder die sind einfach gestresst. Aber alle, wirklich alle männlichen Lehrer, die kommen und die machen Gespräche und die lassen sich Zeit. Die kommen mit Ideen, also, das ist so eine Erleichterung, Befreiung, dass die Kinder die Lehrer hier persönlich, nur für sich, sehen. Weil meine Patienten, das ist meine Erfahrung, die lehnen alle, fast alle, wenn ich vorschlage: "Gut, dann mache ich einen Termin mit deinem Lehrer. Da komme ich irgendwie zur Schule", die lehnen ab.

Das wollen wir nicht. Die hassen Schulgespräche, die haben Angst, die wollen nicht. Es gab eine Aussage, wir werden sowieso in solchen Gesprächen in der Schule einfach fix und fertig gemacht. Die sind alle da, alle Lehrer sind da, Schulleitung ist da und wollen wir nicht. Aber die wollen alle, dass der Lehrer hier kommt und dann ist total anders, dass die einfach von sich raus reden, dass die über Probleme reden. Und das ist hier total andere Atmosphäre. Und ich habe vieles geschafft wirklich mit solchen Gesprächen, hier bei mir. Und die Kinder, die sind total anders darauf. Und die Lehrer, die sehen auch die Kinder total anders, dass sie sagen, "wir wussten ja nicht, dass er solche Probleme hat oder, dass sie einfach Ängste hat". Und dann ist das wirklich, wie gesagt, eine Erleichterung für Kinder.

I: Ja, das glaube ich. Gibt es denn irgendwie Möglichkeiten, die Lehrer zu unterstützen da im Zusammenhang mit psychisch belasteten Kindern in der Klasse?

P: Ich versuche, dass wir irgendwie zusammen ein Dreieck haben und nehme ich einfach Schule und Lehrer sehr, sehr ernst. Und dann gebe ich auch meinen Patienten „Ich bin für dich da.“ Das bedeutet ja nicht, dass ich nicht auf der Seite von Schule bin. Das soll einfach alles klappen, und das ist ja unser Ziel, dass du auch in der Schule weiterkommst. Nicht, dass wir sagen, o.k. alles klar, wunderbar. Dann du bleibst ab morgen zu Hause, machst du alles, was du willst. Das mache ich ja nicht. Aber ich bin bereit, jeden Schritt mit dir mitzugehen. Aber dann musst du auch deine Pflicht machen. Ich mache meine Pflicht. Das ist alles Abmachung, was wir einfach (-). Auch mache ich einen Vertrag, wenn ich Kontakt, so einen engen Kontakt mit Schule komme, dann mache ich erstmal einen Vertrag mit meinen Patienten. Da sage ich, o.k., das ist meine Pflicht, dass ich einfach, ich kümmere mich um Termin, ich spreche Telefonate, dass ich anrufe

und dann musst du auch deine Pflicht machen. Ich glaube, das war immer so, dass die Lehrer gesagt haben, das ist wunderbar, dass sie der Sache nachgehen. Ich glaube, dass soll einfach auch eine Unterstützung auch für Lehrer sein.

I: Gibt es denn irgendwas, was Lehrende noch brauchen an Kompetenzen oder an Unterstützungsmöglichkeiten?

P: Ja, die sollen mehr (-). Ich rede wirklich nur über meine Patienten mit Migrationshintergrund, […] Was wollte ich sagen, jetzt habe ich es vergessen. Die Lehrer, die sollen sich einfach, wie soll ich einfach sagen, die sollen einfach akzeptieren, dass die wirklich aus anderer Welt kommen. Die Kultur ist anders, die verstehen die Welt total anders. Und das ist ja so. Man kann nicht sagen, o.k., also ich komme mit meinen sieben Sachen nach Deutschland, möchte für immer hierbleiben. Aber, ich bin anders. Ich bin anders aufgewachsen, mit anderer Kultur. Und zu Hause werde ich auch anders. Ich habe das Gefühl, dass nicht manche, viele Lehrer, die haben die Hoffnung, o.k., du bist hier und du bist ein Kind, jetzt musst du einfach irgendwie anders denken. Das geht nicht. Das geht wirklich nicht. Ich weiß nicht, ob das einfach eine Hoffnung ist oder ein Muss oder eine Pflicht? "Jetzt musst du irgendwie das machen!" Das geht nicht. Es gibt Lehrer, die auch Migrationshintergrund haben, die haben auch den Bedarf. Wirklich, ich habe auch hier gehabt, dass er (-). Ganz nette, wunderbare Dame als Lehrerin hier, und sie hat erwartet, das Kind soll jetzt wie die Deutschen auch, wie die Gesellschaft denken. Die Eltern, die sollen sofort alles ändern. Dann können wir einfach zu einer Lösung kommen. Und das geht nicht. Das wirklich ja nicht. Das ist immer meine Bitte. Man soll akzeptieren, die sind anders, aber nicht anders schlecht, die sind anders. Also nicht bewerten, schlecht oder gut. Die haben eine andere Kultur. Aber ich

sage ja nicht, wir sollen immer uns anpassen, überhaupt nicht, oder die Gesellschaft soll sich anpassen, überhaupt nicht. Dass man versucht, so machen, dass die Kultur auch nicht schlecht wäre, oder: "Eh warum, Schweinefleisch isst du ja nicht, dann bist du einfach Außerirdischer", also bei uns. Und du siehst auch anders aus, deine Sprache ist komisch, du bist schlecht. Das ist momentan, seit Jahren, dass man das Gefühl einigen Kindern gibt, dass du was anderes bist. Und die werden total anders hier aufwachsen und sehen wir auch, anders werden in der Gesellschaft. Deswegen sage ich den Lehrern, ich weiß, das ist auch nicht einfach, wirklich, das ist nicht einfach, eine außergewöhnliche Leistung. Aber man soll wissen, dass die langsam, die brauchen Zeit. Man soll denen Zeit lassen. Nicht: Erste Klasse, sieben Jahre alt, warum redest du mit Akzent? Das ist ja so, weil die einfach zu Hause, die haben andere Muttersprache. Und die brauchen Zeit. Oder, "deine Eltern, die können überhaupt nicht auf Deutsch reden". Und dann, "Gespräche, das ist unmöglich". Das ist auch ein Punkt. Was die Kinder, die darunter leiden. Dass die ein bisschen Verständnis haben, mehr nicht.

I: Was für Herausforderungen können denn dadurch entstehen, dass man deutsche Kinder und Kinder mit Migrations- oder Fluchthintergrund zusammen in der Klasse hat?

P: Das ist eine Herausforderung, wirklich. Hut ab, so für die Lehrer, die das zusammenkriegen. Aber viele kriegen ja nicht zusammen. Ich habe so viele Kinder, solange diese Kinder irgendwie in Migrationsklassen sind, dass die alle, das heißt, keine Deutschen in der Klasse sind, die fühlen sich gut. Die fühlen sich geborgen, sicher, die lernen einfach wunderbar. Die haben keine Angst, zur Schule zu gehen. Die Schule macht Spaß. Aber wenn die Klasse, die haben,

die sind mächtig eines Tages der deutschen Sprache, die sollen in normale Klasse mit deutschen Kindern gehen. Dann auf einmal haben die keine Lust, zur Schule zu gehen, die wollen überhaupt nicht mit Deutschen zusammen sein. Auf einmal können sie überhaupt nicht auf Deutsch sprechen. Die verstehen auch kein Deutsch. Die Lehrer sind überhaupt nicht nett, die Lehrer achten überhaupt nicht auf die. Die sehen sich so klein. Dann auf einmal kommen Verhaltensauffälligkeiten. Ich habe auch bei einer Achtjährigen „Diagnose“ wegen der Schule.

I: Oh Gott, in dem Alter schon. Reguliert sich das irgendwann?

P: Ja, ich hoffe, […].

I: Sind da denn Konflikte dann auch in der Klasse?

P: Keine Konflikte. (-). Ich weiß es nicht, das ist so stressig, sie hat auf einmal vergessen, dass sie auf Deutsch verstehen kann. Sie kann auch auf Deutsch, sie ist so klasse, sie ist seit XXX Jahre hier. War auch in der Vorschule, alles wunderbar. Sie hat überhaupt kein Sprachproblem. Aber auf einmal, in der XXX Klasse und sie sagt „Ich kann nicht auf Deutsch, ich kann überhaupt nicht. Ich verstehe nicht, wenn die Lehrerin so schnell redet und die anderen. Es gibt Worte, die ich nicht verstehe. Dann möchte ich zu Hause bleiben. Die Eltern haben gesagt, das geht nicht, du musst zur Schule. […].

I: Oh Gott, und das in dem Alter.

P: Deswegen, das ist eine Herausforderung, besonders wenn die zusammenkommen. Weil die Kinder, die werden einfach aussortiert. Die Person nicht und die Person nicht. Und die Lehrerin ist auch Deutsche. Oh Gott, dann bin ich hier fremd.

I: Kommt das denn auch von den deutschen Schülern, dass die ausgegrenzt werden? Oder ist das (-)

I: Ich glaube schon. Wie, die sind langsam, die sind unsicher. Natürlich, wenn man ja zu Hause auch nicht auf Deutsch redet. Man kann ja nicht. Der Wortschatz ist total bedürftig. Die reden total mit anderen, also irgendwie Wörter, andere Sätze, oder Umgangssprache. Die können das nicht und die wissen sehr gering, dass sie einfach sagen. O.k., das ist aber auch, das ist kein Deutsch, was ich überhaupt gelernt habe, das ist kein Deutsch. Die reden anders und die Wörter versteh ich ja nicht. Aber ich glaube nicht, dass die Kinder absichtlich machen. Natürlich, die reden umgangssprachlich von einer deutschen Familie. Es gibt andere Satzbildungen, die reden schnell, das ist ihre Muttersprache, genauso wie bei meiner Muttersprache, dass ich nicht denke. Aber wenn wir einfach auf Deutsch reden, wir denken, wir setzen, wir bauen die Sätze und die Grammatik, das ist alles, das ist alles passiert, dass es langsamer (-). Man ist unsicher, man möchte, dass es alles richtig rauskommt. Gut, das kommt alles nicht richtig raus. Also, Satzbau ist falsch, Grammatik ist auch nicht wie das sein soll. Und dann auf einmal die deutsche Sprache, die sagen einfach, oh eigentlich geht das überhaupt nicht auf Deutsch. Auf einmal sagt sie „Das ist nicht Deutsch was ich rede, das ist was anderes.

I: Ja, ach Gott. Gibt es aber vielleicht auch Ressourcen in diesen Klassen, wenn ich jetzt deutsche Kinder und Kinder mit Migrationshintergrund zusammen habe?

P: Ressourcen, die kommen für mich alles vom Lehrer. Wenn die Lehrer (-). Gut, man kann vielleicht auch nicht erwarten. Das ist ja ihre Pflicht, und die wissen, wer überhaupt die Schüler hier sind. Man soll nicht nur Mathematik und lernen, irgendwie lesen und schreiben beibringen, man soll viele andere, die Ressourcen mitgeben. Das man diese unsichere (-). Die Lehrer, die sind Profi. Die wissen einfach, wer überhaupt sicher ist und wer nicht. Wer kommt mit und wer kommt nicht mit? Wer ist schwach, wer ist sehr stark? Diese Person braucht weniger von mir und diese mehr. Ein bisschen mehr geben, Wärme, Stärke, "oh, du bist ja klasse". Bestimmte positive Sätze mitgeben. Ich warte, dass du morgen auch kommst, du warst heute wunderbar, klasse. Das ist, die Ressourcen mitgegeben, vom Lehrer. Wenn die Eltern in solchen Fällen was mitgeben: „Du weißt auch ja nicht, wie man überhaupt auf Deutsch redet. Du bist auch nicht zur Schule gegangen hier“. Kommt einfach irgendwie von Kindern. Das heißt, "halt deine Klapp, die weißt ja überhaupt nicht einmal mein Problem Bescheid". Ich höre auch ab und zu mal dieses Wort von Kindern, von älteren Kindern. Dann bleibt nur der Lehrer oder die Lehrerin, die mich kaputt machen kann oder so stark, dass ich diesen Weg mit den anderen mitgehen kann. Für mich ist sehr wichtige Rolle, die die Lehrer haben für diese Kleinen, die stärken. Mit ganz einfachen Sätzen, ganz einfachen. […].

…
