# Supplementary material for: Mental health and cultural and linguistic diversity as challenges in school? An interview study on the implications for students and teachers
Source: PLoS One. 2020 Jul 20;15(7):e0236160. doi: 10.1371/journal.pone.0236160 (PMC7371207; doi:10.1371/journal.pone.0236160)
Supplement: S2 Transcripts — (PDF) [file pone.0236160.s002.pdf]

S 2 Transcripts

Excerpts of the transcribed interviews with the teachers

Transkription des Interviews mit Teacher 1

<u>Legende:</u>

Interviewer=„I“; Proband=„P“

Pausen=(...)

unverständliches Wort=((unv.Wort))

Wort- und Satzabbrüche= (-)

- Anonymisierungen=[…]

I: Also, genau, es geht ja bei diesem Interview jetzt um eine Intervention, die ich entwickeln will, die die psychosoziale Belastung bei Kindern reduzieren soll und diese Intervention soll eben in interkulturellen Klassen eingesetzt werden, also in Klassen, in denen Schüler verschiedener Herkunft unterrichtet werden. Und um die Intervention nach den Bedürfnissen der Lehrenden und SchülerInnen, ExpertInnen zu entwickeln, will ich dazu Befragungen machen, um zu gucken, was sind denn eigentlich die Bedürfnisse und genau, was wird noch gebraucht in den Klassen. Die erste Frage ist, welche Erfahrungen hast du im Umgang mit psychisch belasteten Kindern?

P: Ich habe ja jetzt erst mit der-, in diesem Schuljahr wieder begonnen mit einer ersten Klasse und bin wieder eingestiegen in den Schuldienst und wir haben zur Zeit, in dieser ersten Klasse zwanzig Kinder und zwei davon sind Kinder aus einem anderem Land. Wir haben einen Schüler, der aus Land 1 kommt und einen Schüler aus Land 2. Und, sag nochmal die Frage?

I: Welche Erfahrung du hast im Umgang mit psychisch belasteten Kindern?

P: Ja! Und der Junge aus Land 2 ist ganz unauffällig und läuft so mit im Schulalltag. Wir haben aber den Schüler aus Land 1, der seit zwei Jahren in Deutschland ist und in Stadt XY ist, der schwerst-verhaltensauffällig ist und der auch schon bereits dieses Verhalten in der Vorschule gezeigt hat. Und der war nicht an unserer Schule in der Vorschule, sondern in einem anderen Stadtteil und wir wurden dann schon quasi darauf hingewiesen zu Schulbeginn, dass es dort an dieser-, in der Vorschulklasse diverse Vorfälle gegeben hat. Und er dort Kinder bedroht hat, ja, starke Gewaltfantasien hat und auch wirklich, also es hat sich zugespitzt, dass er auch wirklich eines Tages dann ein Kind mit einem Messer bedroht hat. Und er zeigte auch andere

Verhaltensauffälligkeiten, also er hat-, man hat ihn beim Klauen erwischt, es ist ein Handy entwendet worden, das waren wirklich auch also jetzt nicht Kleinigkeiten, die da abhandengekommen sind und das hat er dann aber immer abgestritten und ja es zeigt sich einfach in seinem Verhalten, im sozialen Miteinander vieles was einfach sehr auffällig ist und was nicht so sein sollte und, ja.

I: Ok. Du hast das jetzt schon zum Teil gesagt, aber vielleicht kannst du nochmal zusammenfassen, wie sich diese psychischen Belastungen in der Klasse äußern, also im Unterricht?

P: Ja. Es ist so, dass es teilweise nicht möglich ist ihn ins Unterrichtsgeschehen zu integrieren. Also er ist, wenn man ehrlich sagt, maximal ein bis zwei Stunden am Tag beschulbar. Danach schaltet er hab und er hat auch so zeitweise so gewissen Absencen, dass er nicht mehr kontaktierbar ist, dass er in seiner eigenen Welt ist, so einen Tunnelblick bekommt. Manchmal macht er auch Geräusche dazu, so, so, ja so ein Keifen und guckt die-, er versucht einen bösen-, oder ich weiß nicht, ob er es versucht, er macht auf jeden Fall, wirft er den anderen Kindern böse Blicke zu und schnauft und er hat auch manchmal, das ist dann so, dass er sich auf den Boden schmeißt und zappelt und schon mal ein Erstickungstod nachgeahmt hat und wenn es ihm einfach zu viel wird oder er sich ja in dem Moment unwohl fühlt, ich weiß nicht, was genau dann dahinter steckt, aber nach einer gewissen Zeit des Unterrichts zeigt er halt diese Auffälligkeiten und er ist nicht in der Lage sich dann zu konzentrieren. Was gut möglich ist, also was, wo-, also man könnte halt schon, wenn man ihn begleiten würde und auch direkt-, es gibt ja Tage an denen wir zu zweit sind im Unterricht, also da sind wir in Doppelbesetzung und wenn man ihn direkt an der Seite hat und ihn immer wieder unterstützt und in-, sobald er Auffälligkeiten zeigt, in dem er irgendwie ein anderes Kind triezen will oder so, da einlenkt

und ihn gleich irgendwie in die Schranken weißt, das auf eine nette Art und Weise macht, dann reicht das meistens auch schon aus. Man muss ihn daran erinnern „Komm, konzentriere dich nochmal.“, und das geht dann auch, aber ansonsten ist es schwierig und wenn man alleine im Unterricht ist, ist es ganz schwierig ihn im Auge zu behalten und er macht das ganz subtil, dass er dann zu anderen Kindern geht, andere Kinder bedroht, jemandem verweigert auf die Toilette zu gehen oder dort jemanden gegebenenfalls noch abfängt und das sind halt so Kleinigkeiten im Vorbeigehen zeigt er dann aggressives Verhalten oder hat eine geringe Frustrationstoleranz, wenn jemand seinen Ranzen berührt, dann stellt er sofort demjenigen ein Bein, weil er sagt „Du hast meinen Ranzen berührt, das wollte ich nicht. Du bist da mit dem Fuß gegen gekommen.“ Er sieht dann aber nicht in dem Moment, dass der einfach auch im Weg lag und der andere Schüler gar keine Möglichkeit hatte dort auszuweichen, so. Das sind viele Punkte, die-, ja, er fühlt sich schnell angegriffen. Er ist überhaupt nicht dann einsichtig.

I: Ok. Welchen Einfluss haben diese Ausdrucksformen seiner Belastungen auf die Klasse oder auf die anderen Schüler?

P: Es ist insofern schwierig, dort dann auch Unterricht zu machen, weil man häufig dann einfach damit zu tun hat zu intervenieren und ihn irgendwie unter Kontrolle zu haben. Man könnte jetzt auch nicht-, also gerade in der ersten Klasse ist man viel in Bewegung und versucht den Kindern zu helfen, weil noch viel Bedarf da ist, auch in den Arbeitsphasen und sie noch viele Fragen haben zum Teil und da ist es einfach schwierig ihn an seinem Platz zu halten, dass er nicht andere Kinder im Arbeitsverhalten stören kann oder gegebenenfalls Streit anfängt und deswegen ist es dann schon-, geht es auf Kosten der Förderung der anderen Kinder und auch ihm wird man nicht

gerecht, also er bräuchte eine eins zu eins Betreuung, wo man einfach sagt „So. Und du würdest dann auch was lernen.“ So ist er einfach viel mit anderen Dingen beschäftigt und er braucht manchmal seine Auszeit, wo er dann ein Bild malt, um runterzukommen. Das haben wir schon mit ihm vereinbart und das geht auch. Aber das ist dann natürlich auch erstmal so ein Prozess, bis man dann-, ja er sucht sich die Aufmerksamkeit und das sucht er halt mit seinem Fehlverhalten. Es ist seine Möglichkeit in dem Moment, glaube ich, ich weiß auch nicht, er kann es sicherlich nicht steuern und weiß auch gar nicht, was er da eigentlich macht oder warum er das tut, aber, ja, er braucht viel viel Aufmerksamkeit. Das bedeutet halt, dass man aufgrund dieses Personalschlüssels, wenn man alleine zwanzig Kinder zu betreuen hat, was ja eigentlich noch eine gute Schülerzahl ist, weil normalerweise hat man sechsundzwanzig in einer Klasse, ist es einfach schwierig und wir möchten ihn ja so viele Stunden, wie möglich im Klassenverband halten, weil wir auch merken, dass es eigentlich ihm gut tut. Er möchte das auch. Er möchte dort Freunde finden. Er möchte dazu gehören. Das ist ihm ganz wichtig und er ärgert sich, wenn er dann auch mal wieder aneckt. Aber er, ja, er eckt halt immer wieder an, weil er einfach eine andere Art hat zu kommunizieren. Er hat einen viel aggressiveren Tonfall in der Stimme und ja, das hat er, das kann er nicht so gut kommunizieren manche Dinge, die ihn auch stören, was man einfach sonst dem anderem Schüler sagen würde „Entschuldigung, du stehst gerade im Weg“, er würde dann ihn anbrüllen oder ihn wegstoßen, das ist so eine Art der Kommunikation. Aber das, ja-. Insofern-. Ich glaube, das ist aber auch für die Schüler, man merkt, dass sie sehr sehr hilfsbereit sind und trotzdem, obwohl er schon wirklich viele Kinder bedroht hat-, er hat ein Kind auch mal, mit-, ohne jeglichen Grund, wir konnten es nicht mehr genau nachvollziehen, weshalb er wütend war auf dieses Kind oder weshalb er das gesagt hat, er hat diesem Kind gedroht, es war ein Mädchen und er hat gesagt „Ich weiß,

wo du wohnst und ich werde dort Jungs hinschicken-„ oder „Männer hinschicken und die werden dich umbringen.“ Und diese konkreten Fantasien, die verbalisiert er und die äußert er da in dieser Klasse und trotzdem ist es so, dass die Kinder trotzdem noch Verständnis dafür haben, weil sicherlich auch viele Eltern darüber gesprochen haben, dass er auch eine Vergangenheit hatte, die nicht einfach war und da zeigen sie viel Verständnis und insofern versuchen sie ihn teilweise zu unterstützen oder auch ein bisschen Verständnis dafür zu haben, aber das ist manchmal auch grenzwertig natürlich.

I: Welche Herausforderungen entstehen für die Lehrkräfte in so einer Klasse? Also in Klassen mit belasteten Kindern?

P: Ja die Herausforderung besteht darin, dass man sehr viel das Sozialverhalten schulen muss. Das ist ja in der ersten Klasse sowieso so, dass man viel Zeit investiert in solche, ins, ja um das soziale Miteinander zu schulen und zu fördern, aber da hat er natürlich extreme Defizite und dadurch geht viel Unterrichtszeit eigentlich verloren und man hat-, es vergeht dann halt keine Schulstunde, in der man nicht mit diesem Thema dann doch beschäftigt ist, weil man bestimmte Dinge auch einfach gleich klären muss, damit Lernen überhaupt möglich ist. Also wenn Kinder aus der Pause aufgewühlt zu einem kommen, weil da wieder ein Vorfall war, dann muss man natürlich diese Dinge erstmal klären, um dann weiterzumachen. Und das ist sicherlich eine Herausforderung, dass man das alles parallel laufen lassen muss. Und man muss ihn halt auch im Blick haben, wir müssen unsere Sachen wegsperren, wir haben einen Schlüssel am Pult und ich weiß, ich kann meine Schultasche da nicht einfach so liegen lassen und ich muss immer, egal, was es ist, ob es meine Stempel sind oder so, irgendwie wegsperren. Ich muss immer ein Auge drauf haben und das ist natürlich schon eine andere-, ja, das ist ein ungutes Gefühl, wenn man das weiß, dass da Sachen entwendet

werden können und ja, man möchte ja auch, dass es ihm gut geht. Also man sieht ja, dass er ganz ganz lieber Junge eigentlich ist und dass er auch gerne lernen möchte. Er ist echt wissbegierig dann auch, da hat er dann Momente und möchte gerne was lernen, aber nicht wirklich neben ihm sitzen, man kann nicht nur für einen Schüler da sein und das ist schwierig, finde ich. Zudem hat man noch weitere Termine, natürlich, weil wir dann Hausbesuch schon gemacht haben. Ich habe in zwei Wochen dann noch einen Termin mit der Familienhilfe. Es muss ein Dolmetscher organisiert werden, weil der Vater nur begrenzt deutsch spricht, die Mutter gar nicht und wir möchten natürlich, dass es keine Kommunikationsschwierigkeiten da gibt, gerade bei so einem Thema und das Bedarf natürlich alles viel Zeit, auch die Sozialarbeiterin zu finden, die noch mit dabei ist und auch den Ganztag betrifft es, dass man da Zeiten abspricht, wann man bestimmte Gesprächstermin noch vereinbaren kann, das ist natürlich einfach noch viel zusätzliche Zeit, die man da investiert, weil man ja auch möchte, dass er da einen guten Start hat.

I: Welche Möglichkeiten gibt es, um Lehrende im Unterricht mit psychisch belasteten Kindern zu unterstützen?

P: Also ich hätte mir gewünscht, oder das wird ja jetzt auch angeboten, aber einfach viel mehr Fortbildungen oder auch schon im Studium auch einfach-, das wäre im Rahmen des Studium schon einfach schön, wenn man dort dieses Thema schon, ja, bearbeiten kann. Man hat ja viele Dinge im Studium gemacht, die nicht unbedingt dann so hilfreich waren für das Berufsleben und das wäre schön, wenn man das in das Studium integriert, dass das einfach ein Thema ist, dass man drauf vorbereitet ist, wie man mit traumatisierten, stark traumatisierten Kindern umgehen kann. Man möchte ja auch auf viele Verhaltens-, oder auch das man es überhaupt schon weiß, was ist, was sind die Symptome eines Traumas. Also ich habe mich danach im

Anschluss ein bisschen belesen und weiß, dass dann einfach Kinder, die stark-, also so Müdigkeit ist zum Beispiel auch ein Ausdruck davon. Wir haben auch in einer Parallelklasse ein Kind, was fast schon apathisch dasitzt und dauerhaft müde ist und das kann halt alles ein Zeichen sein für so etwas. Man weiß natürlich nicht, was dahinter steckt und so, aber das sind halt-, dass man weiß, wie man dem begegnen kann, was man nicht machen kann, was das Ganze noch triggert vielleicht gegebenenfalls. Das ist wahrscheinlich bei jedem Kind noch anders. Aber das man Unterstützungsmöglichkeiten hat. Das man weiß, dass man jemanden an der Seite hat. Das jetzt-, ja, es wäre eigentlich schön, wenn-, was natürlich auch nicht möglich ist sicherlich, aber dass die Kinder schon im Vorfeld einfach schon psychologische Betreuung an die Seite gestellt bekommen und das eine Schulbegleitung für solche Kinder auch organisiert werden zeitnah. Ja, das ist ein sehr großer Aufwand und nicht jeder Schüler wird sowas bekommen, da sind die Gelder natürlich auch begrenzt. Wir wissen auch nicht, ob wir ihm auch sowas wirklich ermöglichen können, was ihm aber durchaus helfen könnte und das wäre schön. Ja. Also einfach geschult zu werden in der Thematik und ja, Fortbildungsmöglichkeiten gibt es ja mittlerweile auch, da kann man ja schon auch drauf zurückgreifen, aber das hätte man viel früher schon haben können. Das wäre sinnvoll gewesen.

I: Die nächste Frage wäre, was fehlt noch für den erfolgreichen Umgang mit belasteten Kindern? Da hast du jetzt schon viel zu gesagt. Willst du da noch speziell was zu sagen, oder?

P: Einfach, es fehlt an Personal. Das würde ich sagen. Das ist eigentlich so das Hauptthema immer und wenn man da jemanden hätte, das wäre schon gut. Wenn man das-, geschultes Personal auch. Nicht, dass man da irgendjemanden hinsetzt, der dann als Quereinsteiger auch

gar keine Ahnung hat, das gibt es ja auch, sondern qualifizierte und professionelle Leute dann mit in die Schulen bringt und das ganze dann auch, ja, dass man im Klassenrat natürlich auch ganz anders mit solchen Themen umgehen kann, dass man weiß, wie kann man so ein Thema integrieren.

I: Jetzt kommen wir zu interkulturellen Klassen, die können ja nochmal eine besondere Herausforderung an Lehrkräfte stellen. Welche speziellen Herausforderungen entstehen denn in interkulturellen Klassen, also in Klassen mit Kindern verschiedener Herkunft?

P: Du meinst jetzt sowas wie in Stadtteil XY der Fall war? Also wo man einfach wirklich viele Kinder mit Migrationshintergrund hatte? Einen hohen Anteil oder die Durchmischung jetzt an sich?

I: Also vor allem eben durch die Durchmischung, also auch der Fluchthintergrund, aber auch durch die Mischung von Kindern mit vielen verschiedenen Hintergründen.

P: Ja. Ok. Es ist natürlich jetzt so, dass es-, es wird ja versucht zu durchmischen in-, weil wir in der Schule in Stadtteil YY-, das ist ja ein gutes Einzugsgebiet muss man sagen und der Sozialindex ist da recht hoch, das heißt die kommen aus Familien mit gutem Hintergrund und bildungsnah sind die Familien. Das heißt, die haben eine ganz andere Grundvoraussetzung. Kommen schon in die Schule und können gegebenenfalls schon Lesen. Dann gibt es natürlich die Kinder, die aus diesem-, ja, mit Migrationshintergrund, die noch nicht die deutsche Sprache so gut beherrschen und die haben natürlich starke Defizite, was man dann natürlich besonders noch fördern muss in Extrastunden, dass man das noch versucht irgendwie auszugleichen. Gerade bei unserem Fall, jetzt bei Schülername 1 sieht man auch, dass die Kinder, die aus

dem Flüchtlingsheim kommen natürlich auch merken, dass sie ganz andere Bedingungen haben und dass sie auch materiell gesehen, das ist natürlich auch sehr konträr dann in Stadtteil YY, dass die Kinder, die wirklich alles bekommen in dreifacher Ausführung und die tollsten, schönsten Schulranzen und Schultüten selbstgenäht und weiß ich nicht was und er hat das nicht und er hat die einfachsten Mittel nur. Dennoch er hat sie, aber trotzdem-, sie können ja auch die Gelder bekommen und auch die Mittel bekommen, aber es ist natürlich was ganz anderes und er sieht auch, dass es denen leichter fällt, auch wenn du die deutsche Sprache beherrscht und wenn du dann einen anderen Hintergrund hast, die Eltern auch dahinter stehen und dir helfen können, er kann seinen Vater jetzt nicht unbedingt so auch in der Schule um Hilfe bitten, weil er ist derjenige, also Schülername 1, wäre jetzt derjenige, der am besten die deutsche Sprache beherrscht in der Familie. Er wird von seinem Vater eher zu irgendwelchen Gesprächen gerufen, wenn es um die Behörde geht und so. Und da merkt man schon, dass das den Kindern das auch auffällt und dass es wirklich ein großes Gefälle dort gibt und das frustriert ihn dann auch und er hat dann auch seinen Stolz und möchte nicht zum Beispiel den Anspitzer von jemand anderes haben nur, weil er den jetzt noch nicht von zu Hause mit bekommen hat, sondern sagt dann „Nee, er kann das auch so.“, also er merkt das schon, dass die anderen Kinder da schon privilegiert sind. Aber das ist jetzt nur der Extremfall in Stadtteil YY. Du wolltest ja jetzt eher auf das unterrichtliche-, oder?

I: Allgemein! Also was Dir auch immer dazu einfällt, ob es ums Unterrichten geht oder um Dynamiken zwischen den Kindern.

P: Was in Stadtteil XY der Fall war, war so, dass die Kinder unterschiedlichen Religionen zum Thema gemacht haben. Dass die „Religion 1“ in der Klasse- das war dann ja neunzig

Prozent der Kinder, das „Religion 2“ als Feind gesehen hat und die meinen „du bist Religion 2“, und Religion 2 ist ein Schimpfwort und das war dann schon mal ein Thema, nochmal was bestimmte Kinder ausgegrenzt hat, weil sie halt nicht Religion 1 waren. Das merke ich jetzt hier nicht so und ja, das war in Stadtteil XY schon sehr homogen in den Klassen, weil halt alle in den Ferien dann auch in die XXX gegangen sind und Buch 1 gelesen haben und so. Das wurde jetzt in meiner Schule aktuell noch nicht so zum Thema gemacht, insofern merkt man das halt einfach nur mit finanziellen Mitteln und im Sprachverständnis dann, dass er da schon die Defizite hat. Aber es gibt auch deutsche Kinder, die durchaus dann Schwierigkeit haben im Schulspracherwerb. Also so groß ist das Gefälle in der Klasse jetzt-, also in-, also so intensiv ist es nicht, weil man halt zwei Kinder hat und der Rest der Klasse recht homogen, so.

I: Entstehen auch Herausforderungen für die SchülerInnen in interkulturellen Klassen? So ein bisschen hast du es ja schon angedeutet.

P: Ja, also die-, also für die Kinder ist es halt auch schwer zu verstehen, dass Schülername 1 gerade so reagiert, wie er reagiert und das aggressive Verhalten, das ist befremdlich. Er macht den anderen Kindern Angst. Seine Auffälligkeiten-, er macht manchmal Grimassen, er ist laut, er brüllt, er droht, er wird handgreiflich, das sind Dinge, die den anderen Kindern völlig fremd sind und die im Umgang ganz anders sind. Das ist natürlich-, es schüchtert viele Kinder ein, das führt zu Problemen und da sucht sich Schülername 1 natürlich dann seine Gruppen in anderen, in den Pausen, dass er andere Kinder findet, die auch in seinem Heim wohnen beispielsweise oder seine Sprache sprechen und da isolieren sie sich da so ein bisschen, das merkt man schon. Nichtsdestotrotz versuchen sie oder versucht man, soweit man die Zeit hat, und man findet ja immer so kleine Inseln,

wo man ihn im kleinem Rahmen auch integriert und die Kinder finden dann schon wieder zueinander, aber es ist schon, schon schwierig, weil die Kulturen einfach auch sehr unterschiedlich sind und weil er in seinem Verhalten wirklich schwerst auffällig ist.

I: Welche Ressourcen gibt es denn möglicherweise in interkulturellen Klassen?

P: Die Kinder sind schon sehr-, die üben sich in der Empathiefähigkeit. Also es ist wirklich schon so, wie ich schon anfangs sagte, dass sie auch viel Verständnis dafür aufbringen, dass sie schon wissen, dass es in anderen Ländern Konflikte gibt, die wirklich fern sind von dem, was wir uns hier-, von den Zuständen, die es hier in Deutschland gibt und das öffnet natürlich-, erweitert den Horizont der anderen Kinder. Und sie wissen auch, dass man Rücksicht nehmen muss auf Kinder, die ganz andere Mittel haben. Das ist natürlich auch schön zu sehen, dass dann jemand anderes ihm dann was leiht und sagt „Hier ich hab hier drei Klebestifte, du darfst einen haben.“. Das ist dann sein eigener Stolz, der das dann ablehnt, aber das ist natürlich schön, dass man versucht das zu durchmischen und sicherlich könnte Schülername 1 auch viel aus seiner Kultur mit einbringen, was schön ist und interessant wäre, wenn man es zum Thema machen kann, aber da sind wir im Moment noch sehr vorsichtig, weil man merkt, dass dieses Thema, seine Heimat und so nicht unbedingt etwas ist, was man direkt im Klassenverband ansprechen sollte. Das merkt man schon, dass er da sensibel drauf reagiert und dass da-, man möchte ja auch nichts triggern gerade. Und ja aber das ist natürlich schon eine Möglichkeit auch für die Kinder sich im sozialen Umgang zu schulen, Verständnis aufzubringen und auch zu sehen, dass es nicht allen Kindern leicht fällt.

…

Transkription des Interviews mit Teacher 3

Legende:

Interviewer=„I“; Proband=„P“

Pausen=(...)

unverständliches Wort=((unv.Wort))

Wort- und Satzabbrüche= (-)

Anonymisierungen=[…]

Teacher 3

I: OK, also dann die erste Frage, welche Erfahrung hast du im Umgang mit psychisch belasteten Kindern?

P: Ja, also generell finde ich, dass wir tagtäglich mit psychisch belasteten Kindern auch in Kontakt kommen. Die Kinder bringen ja alle ihre Probleme von zu Hause einfach mit in die Schule. Sei es, ob jetzt ein Streit vorgefallen ist zwischen Geschwisterkindern oder sei es jetzt auch Leistungsdruck, der ja sich auch psychisch belastbar auf die Kinder auswirkt. Aber ich persönlich habe natürlich die Erfahrung gemacht, dass Kinder mit Migrationshintergrund besonders psychisch belastet sind, da sie in ein komplett neues soziales Umfeld kommen und mit einer neuen Sprache konfrontiert auch werden und dadurch natürlich auch an ihre Grenzen kommen. Die sind besonders psychisch belastet diese Kinder. Und diese Erfahrung habe ich gemacht, denn ich hatte drei dieser Kinder im Laufe der letzten drei Schuljahre gehabt.

I: Wie können sich denn diese psychischen oder auch psychosozialen Belastungen bei Kindern in der Klasse und im Unterricht äußern?

P: Generell ist das so, dass die Kinder natürlich nicht regulär am Unterricht teilnehmen können. Sie können dem Unterricht nicht so folgen, wie man's sich vielleicht wünschen würde für die Kinder und auch für den Klassenverband. Es ist so, dass sie einfach nicht mitmachen können, aufgrund der Sprachbarrieren auch. Aber auch aufgrund der sozialen Kontakte, die sie bis dato dann ja auch gar nicht noch geknüpft haben. Es ist ganz wichtig, dass ein Kind sich eigentlich wohl fühlt in einer Klassengemeinschaft und das ist bei den Kindern mit Migrationshintergrund, vor allem die Flüchtlingskinder gar nicht der Fall, die sind ja noch gar nicht richtig bei uns angekommen, die haben eine Vorgeschichte und die müssen das auch alles erstmal verarbeiten. Das spezielle Kind, das ich jetzt in der Klasse hatte, das ist dann

teilweise auch, also es verhält sich einfach nicht regelkonform. Es hält sich nicht an die Regeln, da es vielleicht die Regeln auch gar nicht versteht, aber auch gleichzeitig vielleicht auch gar nicht verstehen will oder vielleicht Aufmerksamkeit auf sich lenken lassen möchte. So kann sich das dann halt äußern, dass es dann zum Beispiel laut wird in der Klasse, aufsteht einfach und raus geht. Zu solchen Situation kam's auch bei mir in der Klasse und man muss dann natürlich immer gucken, wie kann man dem Kind helfen, wie kann man ihm das Gefühl vermitteln, dass er sich in der Klasse auch wohlfühlen kann und gut aufgehoben ist. Ja, aber Verweigerung von Leistung, das ist glaube ich eine der Lösungen, die die Kinder selber sehen, einfach oder auch Schulverweigerung generell. Dass die nicht mehr in die Schule gehen möchten, das gab's auch schon mal. Dass das Kind, dann auch weil es sich nicht mehr wohl fühlte in der Schule, dann auch ja krank wurde und zu Hause geblieben ist. Oder sich vielleicht auch krank gestellt hat, was auch sehr traurig ist. Auch für das Kind selber, wenn es dann auch so weit kommt. Genau, Streitigkeiten mit anderen Klassenkameraden, die kommen dann auch ans Tageslicht, natürlich, auch aufgrund der Sprachbarrieren, der Missverständnisse.

I: Das ist jetzt schon so praktisch 'ne kleine Überleitung zur nächsten Fragen, welchen Einfluss denn diese psychosozialen Belastungen und auch die Äußerungen dieser Belastung auf die Mitschüler in der Klasse haben können?

P: Ja, die Konsequenz natürlich ist dann auch, dass sich Kinder streiten, dass vor allem auch die Kinder, die dann natürlich hier auch immer zur Schule gegangen sind, also unsere Stammklassen, dass die sich auch ein bisschen gestört fühlen in ihrem gewohnten Umfeld. Sie müssen ja praktisch immer auf jemanden zugehen und immer dieses Verständnis für die Kinder aufbringen und das sind auch die

Grenzen, die unsere Kinder auch einfach, das sind halt deren Grenzen und es ist auch schwierig, wenn wir von den Grundschulkindern erwarten, dass sie alle fremden Kinder praktisch mit offenen Armen empfangen und für alles Verständnis aufbringen müssen, dass können die ja auch gar nicht leisten. Diese Konflikte, die dann entstehen, sind ja auch natürlich meistens auch Konflikte zwischen Kindern, die dann wenig Verständnis dafür aufbringen können und nicht verstehen, warum sie denn immer nachgeben müssen. Also ich mein jetzt damit auch die deutschen Kinder. Also jetzt speziell in meiner Klasse war das halt so, dass ich dann natürlich die Migrantenkinder hatte, die Flüchtlingskinder und dann auch die Kinder, die ich von der zweiten Klasse an begleitet habe. Es gibt einfach Kinder, die sowieso schon sozial auffällig sind und selber mit sich selbst nicht klarkommen im Schulalltag und dann auch noch Verständnis aufbringen müssen für diese Kinder, die dann die Sprache nicht kennen und sich dann auch nicht an die Regeln halten. Das verstehen die dann halt nicht, warum diese Regeln dann nicht für diese Kinder dann auch gelten. Genau, der andere negative Einfluss, genau, das geht ja darum, ist auch, dass der normale, der reguläre Unterricht absolut gestört ist. Also wir können keinen normalen Unterricht machen, weil wir uns dann um die Streitigkeiten und um die Probleme dieser Kinder dann kümmern müssen.

I: Ist ja genau schon zum Teil dann auch die nächste Frage, welche Herausforderungen denn dadurch dann für die Lehrenden entstehen?

P: Naja, die Herausforderung ist, dass man nicht, man wird, man kann einfach nicht nach dem Lehrplan unterrichten. Also man kann's schon, man muss das auch natürlich auch, aber es ist so, dass man einen Spagat machen muss zwischen: wie bring ich überhaupt dem Kind mit Migrationshintergrund was gerade erst zu uns frisch in die Klasse gekommen ist, die deutsche Sprache näher, wie

bring ich ihm, wie leiste ich die Wortschatzarbeit und auf der anderen Seite muss ich dann zusehen, dass ich Lehrplan orientiert auch mit den anderen Kinder daran weiter arbeite, dass die auch weiter kommen, denn die bekommen ja auch ihre Zeugnisse, die bekommen ihre Noten und stehen auch unter einem gewissen Leistungsdruck. Da müssen wir als Lehrer gucken, wie werden wir beiden Seiten gerecht. Wie können wir das dann machen? Mit welchen Materialien vielleicht, mit welchen vorbereiteten Fragebögen, was brauchen wir dafür vielleicht? Also das ist eine enorme Herausforderung sowohl sozial als auch vom, ja von den Leistungen her.

I: Gibt es irgendwelche, und wenn ja welche, also irgendwie Unterstützungsformen in solchen Situationen für die Lehrenden?

P: Also eine Unterstützungsform ist es für mich auf jeden Fall gewesen, dass wir damals eine Sprachlernklasse gehabt haben, wo die Kinder in festen, zu festen Zeiten hingehen konnten. Das heißt also, dass man sich irgendwo die Arbeit auch mit einem anderen Kollegen geteilt hat. Dass die Kinder dort, wusste man, dort können die Kinder Wortschatzarbeit machen, dort können denen die deutsche Sprache näher gebracht werden und man selbst konnte dann im Unterricht überlegen, wo kann ich andocken, um das Kind einfach mit in die Klassengemeinschaft, mit in den regulären Unterricht aufzunehmen. Wo gibt's da wirklich vielleicht Punkte, wo ich ansetzen kann? Das war bei diesem einen speziellen Kind bei mir ganz schwierig, das zu leisten, denn er war vom Leistungsstand einfach nicht, er hatte nicht den gleichen Leistungsstand, wie die anderen Kinder, obwohl das von den Eltern behauptet worden ist, dass er genauso weit ist, wie die Drittklässler dann halt auch sein sollten, die gleichen Grundrechenarten, dass er die schon alle kennt, dass er alle Lerninhalte schon kennengelernt hat, so wie unsere Drittklässler. Das war aber leider gar nicht der

Fall. Und da sind wir absolut an unsere Grenzen gekommen und geholfen hat's mir, wenn ich eine zweite Person in der Klasse auch mit hatte, sei's jetzt ein Förderschullehrer oder auch einfach nur ein FSJler, der neben dem Kind sitzen konnte und auch helfen konnte. Vielleicht auch mal ein Arbeitsblatt zu bearbeiten, ein ganz einfaches. Denn die Kinder sind einfach mit den kompletten, mit diesen wechselnden Aufgabenformaten, mit den verschiedenen Arbeitsblättern auch komplett überfordert. Und helfen würde natürlich auch, wenn man festes Material hätte, Arbeitsmaterial, was man als Lehrkraft einsetzen kann. Das habe ich versucht mit bestimmten Arbeitsmitteln. Weiß nicht, ob das dann noch später gefragt wird. Aber das hat einigermaßen entlastet, wobei man trotzdem immer wieder gemerkt hat, die Kinder haben wirklich nochmal das Bedürfnis auch mit der Lehrerin zu sprechen und auch mal von der Lehrerin etwas erklärt zu bekommen. Das gilt natürlich, das gleiche gilt für die Kinder, für die normale Klasse, wo Lehrplan orientiert weiter unterrichtet werden muss. Und da muss man auf die Selbstständigkeit der anderen Kinder so ein bisschen hoffen, was man aber nicht von denen erwarten kann.

I: Gibt es noch was, was fehlt, was helfen könnte, was man in so einer Situation bräuchte, als Lehrender?

P: Ja, also fehlen tut bei uns jetzt auf jeden Fall die Sprachlernklasse, wie ich finde. Also weil das einfach nochmal ein Ort war, wo die Kinder zusammen kamen und wussten wir alle kennen die deutsche Sprache nicht gut und können da nochmal ansetzen. Und diese Sprachlernklasse ist ja bei uns an der Schule jetzt nicht mehr, soll so nicht mehr stattfinden. Das war etwas ganz organisiertes und etwas ganz tolles. Ich hab zum Beispiel eine Schülerin, die ist in der zweiten Klasse zu uns dazu gestoßen als Flüchtlingskind und hat enorme Fortschritte durch diese Sprachlernklasse einfach gemacht. Sie konnte zum Ende

der vierten Klasse Lehrplan orientiert mitarbeiten. Natürlich keine Aufsätze schreiben, wie die Kinder es bei uns dann halt auch wirklich leisten müssen. Aber sie ist Lehrplan orientiert mitgekommen und hat teilweise dann auch Noten bekommen zum Ende hin. Das war eine absolute Bereicherung diese Sprachlernklasse. Wenn die natürlich nicht mehr da sind, ist das sehr schwierig das einfach als Lehrkraft im Klassenverband zu leisten, Wortschatzarbeit zu machen oder die deutsche Sprache näher zu bringen, diesen Kindern und dann gleichzeitig wie gesagt, mit den anderen weiter zu arbeiten. Das schafft man einfach nicht. Also das heißt es fehlt eigentlich Arbeitsmaterial, für uns als Lehrkräfte, irgendwelche Hefte, die wir den Kindern an die Hand geben können, die selbsterklärend sind. Dass das auch einheitlich gemacht wird und nicht von jeder Lehrkraft unterschiedlich. Also dass wir alle gleich das handhaben und vielleicht auch Arbeitsmaterial, was von der ersten bis zur vierten Jahrgangsstufe dann auch geht, dass man weiß, das kann ich mir raus nehmen, damit kann ich mit dem Kindern arbeiten. Also das fehlt auf jeden Fall. Personell fehlt sowieso immer irgendwas an der Schule, also das man auch mal Unterstützung in der Klasse hat. Manchmal ist es einem gegönnt. Das wurde dann halt wirklich in meiner Klasse auch gemacht, dass ich FSJler zwischendurch bekommen habe und die meist für eine Stunde da waren, um mit dem Kind zu arbeiten, hat sehr geholfen. Und das ist aber, das weiß ich, das ist nicht an jeder Schule möglich und nicht an jeder Schule vorhanden. Also personell müssten wir auf jeden Fall besser aufgestellt sein.

I: Ok, jetzt geht's so ein bisschen spezieller um interkulturelle Klassen. Im Endeffekt, genau, also Klassen mit Kindern verschiedener Kulturen. Das ist ja wahrscheinlich jede Klasse an Schule XY. Aber welche speziellen Herausforderungen können denn dadurch entstehen, dass man eben Kinder aus verschiedenen Herkunftsländern in einer Klasse hat?

P: Also wir hatten zum Beispiel in unserer Klasse den Fall, dass wir das Thema Weltreligion natürlich auch besprochen hatten, das war ein sehr bereicherndes Thema für uns, für alle Kinder, weil alle Kinder auch interessiert waren, daran wie, was ist denn überhaupt eine andere Religion. Mit dem Thema Religion konnten die Kinder sowieso nicht so viel anfangen, aber dass sie kennengelernt haben, dass ein Kind zum Beispiel den Ramadan mitgemacht hat, also die Fastenzeit, dass man da auch wirklich nochmal nichts essen durfte. "Warum macht das Kind das überhaupt?" Also, dass die Kinder Fragen stellen durften und das miterlebt haben, das war eine absolute Bereicherung für uns alle. Wir haben auch gesehen, dass man da natürlich auch an die Grenzen kommt, das zu verstehen, warum das dann doch tatsächlich gemacht wird. Also das Interesse der Kinder für andere Kulturen und Religionen ist da und das kann man natürlich auch wunderbar umsetzen im Unterricht und besprechen. Das war auch ganz viel, es gab viel Anlass für uns, uns dann auch verschiedene Filme anzuschauen über die verschiedenen Kulturen und Religionen. Das kann man natürlich nur mit den älteren Kindern machen, aber auch schon mit den jüngeren anreißen. Auf jeden Fall ist es so, dass man da- jetzt hab ich den Faden verloren.

I: Also, jetzt ging's vor allem um Herausforderungen, die entstehen, aber, gleich kommen auch noch Ressourcen, von daher.

P: Genau, das waren jetzt die Bereicherungen. Die Herausforderung ist natürlich, dass die Kinder so unterschiedlich vielleicht auch kulturell erzogen worden sind, dass sie dann halt vielleicht dann auch kein Verständnis für aufbringen können, wie sich ein Kind verhält oder wie es vielleicht von zu Hause aus erzogen worden ist. Also, das kann natürlich auch zu Missverständnissen kommen, auch

die Reaktion von bestimmten Kindern, kann zu Missverständnissen führen. Ja, also wir müssen mal gucken, dass wir als Lehrkräfte eigentlich alle Kinder unter einen Hut, in der Klasse unterrichten und unter einen Hut bringen, aber das ist halt manchmal nicht möglich, weil je nach Zustand des Kindes, wenn ich jetzt an das Kind denke, was tatsächlich an dem Tag im Sommer nichts gegessen hat und auch überhaupt nicht zu Leistungen fähig war. Das wohl aber freiwillig laut Eltern gemacht hat, die Fastenzeit mitgemacht, dann gerate ich, komme ich da auch an meine Grenzen. Ich kann einfach die Kinder dann ja nicht zu Leistung zwingen, obwohl sie eigentlich da mitmachen müssten in der Schule.

I: Gibt's irgendwelche speziellen Herausforderungen für die Schüler und Schülerinnen in den Klassen, wenn die Mitschüler aus verschiedenen Kulturkreisen kommen?

P: Ja, das war ja dieser Punkt, wo ich sage, je nachdem wie man erzogen worden ist. Also, es gibt ja auch Kinder die dann zum Beispiel ein Kopftuch in der Schule tragen und die anderen Kinder verstehen das vielleicht auch gar nicht "Warum trägt sie ein Kopftuch?". Richtig erklärt wird's eigentlich nie. Man muss das irgendwie so hinnehmen. Aber dadurch kann es ja auch zu Ausgrenzungen kommen, dass man aufgrund des Unverständnisses, also dass man wenn man's nicht versteht, dann distanziert man sich ja auch von diesen Kindern. Und dadurch kann's ja auch zu sozialen Problemen kommen, dass die Kinder wie gesagt ausgeschlossen werden, keine Freunde haben. Ich finde das ist eine große Gefahr bei uns, weil man sich dann ja auch sehr absondert, also dass man sich dann-

I: Gibt es denn vielleicht auch Ressourcen, die in diesen Klassen sind, dadurch dass Kinder verschiedener Kulturen zusammen unterrichtet werden?

Teacher 3

P: Ja, auf jeden Fall kann man, also das hatte ich ja gerade schon angesprochen, das Thema Bereicherungen. Also allein für das Thema Religionsunterricht, für das Fach kann man natürlich viele Facetten rausziehen, wenn man verschiedene Kulturen in der Klasse einfach hat. Man kann Eltern in den Unterricht einladen und erzählen lassen, "wie läuft bei uns ein besonderes Fest ab oder ein besonderer Feiertag", "warum feiern wir was". Also das Interesse der Kinder ist auf jeden Fall vorhanden für verschiedene Kulturen und kann ja auch den Horizont der Kinder erweitern. Manche haben vielleicht, sind vielleicht durch die Eltern oder durch die Gesellschaft, haben bestimmte Vorurteile, die dann auch abgebaut werden können. Wenn man dann natürlich verschiedene Kulturen kennenlernt. In unserer Gesellschaft heute, die ja wirklich multikulturell ist, ist das nun mal auch eine Bereicherung, wenn man über Kulturen im Unterricht spricht, wenn man die dann halt auch auslebt vielleicht, dass man vielleicht eine Mutter was mitbringen lässt, Gebäck oder sonstiges, dass man verschiedene Kulturen auch kennenlernt. Also, das kann man wunderbar in den Unterricht integrieren, finde ich.

…

Transkription des Interviews mit Teacher 4

Legende:

Interviewer=„I“; Proband=„P“

Pausen=(...)

unverständliches Wort=((unv.Wort))

Wort- und Satzabbrüche= (-)

Anonymisierungen=[…]

Teacher 4

I: Meine erste Frage ist, welche Erfahrungen Sie haben im Umgang mit Kindern mit psychischer Belastung.

P: Also, ganz unterschiedliche Erfahrungen, also, einige Kinder sind total zurückgezogen und öffnen sich gar nicht, reden ganz wenig und sparen das Thema komplett aus. Man merkt ihnen aber an, dass mit ihnen was irgendwie nicht stimmt, dass sie irgendwie unzufrieden und traurig sind. Und vor allem bei Jungen beobachte ich eher so ein extrovertiertes Verhalten. Die müssen ihr inneres Unwohlsein, die Aggressionen, die sie dann auch haben, mit Bewegung rauslassen, mit verbalen Aussetzern. Also oft sind die Jungen, die ich dann in Klassen hatte eher zu merken als die Mädchen. Die waren eher so zurückgezogen und still.

I: Wie groß ist denn der Anteil, wie häufig kommt das vor, dass Kinder psychisch belastet sind?

P: Also, insgesamt finde ich, gibt es viele Kinder, die psychisch belastet sind. Angefangen davon, wenn die Eltern sich getrennt haben, wenn dann auch immer wieder Streitigkeiten in den Familien sind. Das ist ja schon eine psychische Belastung für Kinder und das ist heutzutage also ganz, ganz viel Thema im Montagmorgenkreis. Ja, also ich habe das Gefühl, das hat wirklich auch zugenommen. Und eben durch die Migranten, die jetzt zunehmend in die Klassen kommen, die ja auch bestimmte Erlebnisse mitbringen, nimmt das eher zu.

I: So ein bisschen hatten Sie es ja schon gesagt, wie äußern sich denn diese Belastungen auch in der Klasse und in der Schule?

P: Also, es fängt an mit solchen Sachen, wie Müdigkeit, einfach, weil sie ganz, ganz spät ins Bett kommen, weil sie Dinge mitbekommen, die sie eigentlich nicht mehr

mitbekommen sollen und dann nicht einschlafen können. Können sich ganz schlecht konzentrieren, ganz schlecht auf die Dinge einlassen, die dann so am Schulvormittag von ihnen abverlangt werden. Viele Kinder haben motorisch auch ganz große Defizite, aus unterschiedlichsten Gründen. Aber ich könnte mir vorstellen, dass das auch einer ist. Also, es wird sich halt nicht mehr so viel beschäftigt mit den Kindern, so mit der Schere irgendwas schneiden oder basteln. Sondern viele sitzen dann auch eben vor den Medien, vorm Handy und so und dadurch verkümmert das, sie sind weniger draußen insgesamt. Das sind alles so Faktoren, die da mit eine Rolle spielen, dass das Lernen insgesamt auch in der Schule schwieriger wird. Für die Kinder besonders.

I: Welchen Einfluss haben denn diese Ausdrucksformen, die diese Kinder zeigen, ihre Symptome, auf die anderen Kinder in der Klasse?

P: Also, wenn sie so still zurückgezogen sind, dann hat das nicht so große Auswirkungen, außer das sensible, empathiefähige Kinder eben merken, da stimmt irgendwas nicht und dass sie sich Sorgen machen und dann zu mir kommen und sagen „Mit der ist was und wir müssen uns kümmern". Und ja, dies andere Verhalten, das schürt natürlich auch Aggressionen. Also, wenn den Jungen auch an Mädchen Aggressionen entgegengebracht wird, werden die natürlich auch unzufrieden und das steigt so langsam an. Es passiert dann immer mehr. Kinder wehren sich. Man muss ständig darüber sprechen. Also, das ganze soziale Miteinander, das geht eher in so eine negative Richtung.

I: Und welche Herausforderungen entstehen dadurch für die Lehrkräfte?

P: Naja, man muss halt ständig, vor allem eben nach Pausen, wo die Kinder dann mehr Freiheiten haben, mehr haben als sonst, wo sie das dann auch rauslassen, muss

man das aufarbeiten. Man muss erst einmal über die Konflikte sprechen, bevor man sich dem eigentlichen Unterricht zuwenden kann. Manchmal ist es auch so, dass die dann in der Klasse auch noch aufeinander losgehen und sich dann hauen und man muss die auseinanderziehen. Und das ist manchmal bei den Erstklässlern schon echt eine Herausforderung. So dass wir auch zu zweit manchmal dann zusehen müssen, dass die sich nicht gegenseitig verletzen.

I: Gibt es da irgendwelche Unterstützung für die Lehrkräfte?

P: Also, wenig. Ich bin ja Beratungslehrerin hier an der Schule und dieser Umgang mit Wutgefühlen und so, das mache ich auch an der Beratung zum Thema. Teilweise einzeln, teilweise so in Kleingruppen. Und das ist so eine Sache, die ich auch anbiete hier an der Schule. Aber im Großen und Ganzen ist man als Lehrerin schon eher, sage ich, damit alleingelassen und viele Kolleginnen bräuchten aber Unterstützung. Das ist schon das, was ich wahrnehme. Und kommen dann auch und fragen "wie machst du das und was kann ich denn jetzt noch machen?" Dann versuchen wir, die Eltern da auch mit ins Boot zu holen, aber das ist nicht immer so einfach. Weil die selbst oft an ihre Grenzen gekommen sind und auch mit diesem Leben, was ihnen so abverlangt wird, teilweise überfordert sind. Das erlebe ich in dieser Elterngeneration ganz verstärkt.

I: Also ist da keine Bereitschaft der Eltern oder einfach keine Kapazität?

P: Ich glaub, sie haben einfach, viele haben keine Kraft. Also, wir haben hier Klassen, wo wirklich fast alle Kinder in der Nachmittagsbetreuung sind, die meistens zwischen 15.00 und 17.00 Uhr dann abgeholt werden. Das heißt, die sind von morgens um 8.00 bis nachmittags 17.00 Uhr in der Schule. Die Eltern arbeiten beide, um sich überhaupt dann hier ein Leben in Ort X leisten zu können. Es ist wenig Kraft

dann noch da, dem entgegenzukommen und auch der Wille, dann wenigstens die wenigen Stunden, die man miteinander verbringt, die sollen dann nicht auch noch, also anstrengend sein für alle, sondern einfach nur schön und entspannt.

I: Was würde den Lehrern und Lehrerinnen denn helfen, also, wie könnte man die unterstützen?

P: Also, tatsächlich wäre es hilfreich, wenn man irgendwie so Programme hätte. Das gibt es ja auch schon. Wir haben jahrelang so „fit und stark“ zum Beispiel gemacht, das ist so eine Unterrichtseinheit verpflichtend. Immer eine Stunde in der Woche mit den Erst- Zweitklässlern, wo es eben um das soziale Miteinander geht. Es gibt das Programm „Faustlos“. Da gibt es, glaube ich, eine Menge Sachen auch auf dem Markt. Das Problem ist einfach, dass wir inzwischen in der Schule so zu sind mit allmöglichen Zusatzprojekten, die ganze Medienerziehung ist dazugekommen, wir müssen Theater unterrichten wöchentlich. Also, wir haben so viele Sachen, die dazugekommen sind. Wir haben im Prinzip gar keine Zeit, uns um dieses wichtige Thema zu kümmern. Weil auch das Curriculum so voll ist, dass wir eigentlich immer nur versuchen, das irgendwie durchzuhechten und das andere läuft einfach so nebenbei. Und das ist eigentlich schade, weil ich merke, dass es an der Stelle wirklich oft brennt.

I: Gibt es denn vielleicht Fähigkeiten oder vielleicht Kompetenzen, die bei Lehrkräften besonders hilfreich sind? Vielleicht welche, die da besser mit klarkommen und so, weil sie besondere Eigenschaften besitzen?

P: Also, ich merke schon, dass die Kollegen, die selber besonders empathiefähig sind und die auch bereit sind jeden Tag irgendwie neu anzufangen mit bestimmten Kindern, denen immer wieder eine Chance zu geben und die auch, sage ich mal, eine sonderpädagogische Haltung (-). Wir

haben einige Sonderpädagogen bei uns in der Schule, die kommen mit diesen Kindern besser klar als andere, die vorwiegend diesen Lehrauftrag für sich im Fokus haben.

I: Was gibt es denn für spezielle Herausforderungen durch interkulturelle Klassen? Klassen, in denen sowohl deutsche als auch Kinder mit Migrations- oder Fluchthintergrund gleichzeitig unterrichtet werden.

P: Also, zum einen ist es so, dass viele dieser Kinder natürlich nicht auf dem Niveau unterrichtet werden können, wie die deutschen, weil die einfach die Sprache noch nicht sprechen. Viele, ja andere Dinge auch noch nicht mitbringen. Das heißt, sie bekommen immer, nicht alle, aber viele doch, bekommen immer Extraaufgaben, brauchen immer irgendwie eine Unterstützung. Entweder eine zusätzliche Lehrkraft oder ein anderes Kind. Weil die alleine das nur schwer bewältigen können. Oder sie bekommen eben komplett andere Aufgaben. Also die haben immer so ein Stück, so eine Art Außenseiterrolle, was sie auch spüren, was sie aber nicht wollen. Meine Kinder, die Migranten sind, die sagen ganz oft „Ich kann das alleine, ich brauch keine Hilfe.“ Aber ich sehe, dass sie doch Hilfe brauchen. Aber sie wollen diese Extrarolle eigentlich gar nicht. Und das macht sie also auch unzufrieden. Ich habe ganz nette Leute, die kommen und helfen sogar ehrenamtlich. XXX die immer zu mir kommt zweimal die Woche. Und die will helfen, aber ich merke immer schon, die Kinder wollen da eigentlich nicht hin, weil sie es nicht als (-). Die wollen keine besondere Rolle haben. Müssen sie aber bekommen, damit man sie überhaupt weiterbringt. Das ist so ein bisschen die Schwierigkeit. Und die haben natürlich auch teilweise wirklich Dinge erlebt, wo einem Angst und Bange wird, wenn man das so hört. Also, die erzählen dann manchmal eben auch vom Opa, der mit abgehackten Armen da irgendwie vor ihnen stand, als sie noch ein kleines Kind waren. Also, die haben so Sachen erlebt, die sie ja eben auch erzählen. Und

das sind eben Themen, die nicht unbedingt für unsere deutschen Kinder hier aus Ort XY wirklich so präsent sind.

I: Welche Herausforderungen entstehen denn dadurch für Lehrkräfte?

P: Naja, also, man muss noch mehr arbeiten als sonst. Also, man muss immer Extramaterialien haben. Man muss noch geduldiger sein als sonst, weil die Klasse an sich einem schon ja auch ganz viel abverlangt an Herausforderung. Und wenn man diese Kinder dazu bekommt (-). Die kann man, man kann im Prinzip diesen Unterschieden nicht gerecht werden. Und das macht ja Stress. Also, weil man im Prinzip ständig irgendwie denkt „Ach warum, du kannst hier gar nicht alle bedienen und die schon gar nicht." Und das ist irgendwie dann auch unbefriedigend. Und ich habe einige Kollegen, die kurz vorm Burn out sind, obwohl das hier alles total schön aussieht und so, aber weil die einfach so einen hohen Anspruch haben und mit dem, was sie dann schaffen und überhaupt gar nicht schaffen können, nicht zufrieden sind.

I: Entstehen auch Herausforderungen für die Kinder in solchen Klassen?

P: Naja, also die Kinder, die so normal mitlaufen, die guten, die bekommen halt weniger Aufmerksamkeit als sonst, weil die halt einem dann halt wo anders abverlangt wird. Also, die laufen so mit. Und ich höre auch ganz oft von Kolleginnen, das die sagen, „Mensch, es tut mir so leid um die, die auch mal Zuwendung bräuchten." Die wird absorbiert von anderen Kindern von denen. Die am lautesten schreien, oder die, die am schwächsten sind.

I: Wie ist denn das zwischen den Kindern? Merkt man da irgendwelche Probleme oder Reibereien?

P: Eigentlich, naja, also eben wie gesagt, Aggressionen, klar wenn sie sich dann streiten wegen irgendwelcher Geschichten. Das schon, na klar, das merkt man, das drückt dann auch auf die Stimmung. Aber eigentlich gehen die Kinder noch am selbstverständlichsten mit diesen Unterschieden um, gerade mit den Migranten. So, sie versuchen dann oft, also die sprechen eben gebrochen deutsch und oft verstehe ich sie dann auch nicht. Und dann versuchen die Kinder, das zu übersetzen. Also, die fühlen sich da dann ganz kompetent, die haben selber nicht so viel Probleme damit. Haben sich jetzt für Halloween verabredet und wollten dann, also die eine Familie hat keine Email Adresse und mit Handy ist auch schwierig. Dann haben sie so überlegt, wie sie so zueinanderkommen können. Die gehen damit so recht unkompliziert um.

I: Was gibt es denn eventuell für Ressourcen in interkulturellen Klassen?

P: Also, was es für positive Dinge mit sich bringt und so?

I: Genau.

P: Also, wir haben zum Beispiel neulich, also bei uns gibt es ein Geburtstagsritual, da bringt immer jemand einen Kuchen mit und dann setzen wir uns in den Kreis und es gibt ein ganz bestimmtes Ritual. Am Ende gibt es eine Wunschrunde. Jedes Kind äußert einen Wunsch und das Kind, was dann Geburtstag hat ist immer ganz beseelt und glücklich. Und neulich, also mein Schüler 1 aus der Klasse, der kommt aus Land 1, da sagte er, wir müssen in der letzten Stunde feiern, meine Mama bringt den Kuchen. Und dann war es zum Beispiel nicht so, dass die Mama den Kuchen brachte, sondern die gesamte Familie war da. Mit einer Mega-Torte, wo ein Bild mit Marzipan abgebildet war. Die hatten zwei Säcke in der Hand. Da war dann Party, also, alles für eine Party. Dann haben sie alles rausgeholt. Also,

die wollten Party feiern bei uns in der Klasse. Das war offensichtlich. Und dann habe ich gesagt, o.k., dann lass ich das jetzt. Dann haben wir ein richtiges Fest gefeiert, Luftballons wurden aufgeblasen, Musik wurde angemacht. Die Eltern haben mit den Geschwistern getanzt. Ein Bruder wurde noch aus einer anderen Klasse geholt. Also, die haben mal so Geburtstag gefeiert wie, also, wie die das da dann anscheinend machen. Also und am Ende des Tages sagte ein Mädchen „Das war die tollste Stunde in meiner ganzen Grundschulzeit.“ Also, das war ganz, ganz süß und die Eltern haben da auch mitgetanzt. Die Mutter eben verschleiert und der Vater hat dann mit rumgerockt. Und wir hatten noch eine andere Kollegin mit dabei. Also, mal eben wurde sowas dann gemacht. Einfach, weil die das wohl so feiern. Das war zum Beispiel süß. Und ein anderes Kind aus einer anderen Klasse, was auch ganz vernachlässigt ist, wo wir uns alle große Sorgen machen, da haben die Eltern, von denen wir eigentlich glauben, dass sie sich gar nicht gut kümmern, haben die ganze Klasse mit Eltern eingeladen und hatten ein ganz tolles Buffet aufgebaut. Und in zwei Räumen, wo sie mit vielen Familienmitgliedern wohnen, hatten eine Discokugel organisiert und haben da bis nachts mit den Eltern der deutschen Kinder getanzt und mit der Klassenlehrerin und so. Und das hat alles in diesem Ort 2 stattgefunden. Das war wohl ein ganz tolles Fest. Also, dieses feiern an sich zum Beispiel, das scheint bei denen irgendwie noch einmal eine andere Rolle zu haben als bei uns. Und das finden unsere Kinder und Eltern ganz toll.

I: Das glaube ich, schön.

P: Und das man einfach auch über die anderen religiösen Rituale spricht und solche Geschichten. Also, dass, der eine hat dann gefastet und dann habe ich gesagt „Sport ist dann ja schwierig.“ Dann würde ich es nicht so schön finden, wenn du dann jetzt beim Sportfest mitmachst, wenn du jetzt gar

nichts gegessen hast und so. Solche Themen sind einfach dann noch präsenter, als vorher.

I: Wie gehen die Kinder damit um, wenn jetzt einer von denen Rituale hat, die sie gar nicht kennen?

P: Auch ganz offen eigentlich. Du weißt das doch, sagt ein Mädchen zu mir. Ich belohne manchmal für irgendwelche Sachen. Und es gibt dann eben auch mal ein paar Naschies oder die dürfen dann eben keine Gelantine essen, und in Gummibärchen ist Gelantine. Und ich vergesse das immer wieder. Und dann erinnern mich die Kinder da dran. Also, für die ist das irgendwie so alles relativ normal.

…

Transkription des Interviews mit Teacher 5

Legende:

Interviewer=„I“; Proband=„P“

Pausen=(...)

unverständliches Wort=((unv.Wort))

Wort- und Satzabbrüche= (-)

Anonymisierungen=[…]

Transkription des Interviews mit Teacher 5

I: Dann lege ich mal los. Und zwar ist die erste Frage: Welche Erfahrung hast du im Umgang mit psychisch belasteten Kindern?

P: Also, genau, wie du es eben angedeutet hast, das ist natürlich schwierig, da die Grenze zu ziehen. Weil, die Kinder kommen hierher und ich weiß nicht, was sie alles im Gepäck haben. Ist ja klar. Aber natürlich gibt es dann Kinder, wo du irgendwann mit der Zeit mitkriegst, da ist auf jeden Fall eine Menge im Gepäck. Ich habe in dieser Klasse hier zwei Kinder, wo es mir ganz deutlich ist. Eins mit einem, es ist ein Flüchtlingskind, wo ich auch ganz lange gebraucht habe irgendwie überhaupt zu merken, wo der ist. Also, weil es auch so diffus war, was er für Schwierigkeiten hatte. Im Lernen, auch im Verhalten und so. Es war extrem schwankend und das konnte ich ganz schwer einsortieren. Und ein Kind, das einfach so ganz zurückgezogen in sich ist. Das ist aber, hat keinen Migrationshintergrund oder so. Aber die Eltern sind getrennt. Das wusste ich auch. Aber ich wusste nicht, wie damit umgegangen wird und wie das Kind auch damit umgeht und so. War aber ganz verschlossen und wenn man versuchte, mit ihr zu sprechen und so. Auch jetzt immer noch. Es ist ganz schwer, wirklich was rauszukitzeln und was zu erfahren von ihr. Aber das ist irgendwie klar. Es gab dann auch eine Situation, wo wohl von Problemen die Rede war, und dann sprach ich es direkt an. Unter vier Augen. Und dann kamen gleich die Tränen und sei alles durcheinander, seit Mama weg sei und so. Also, das ist einfach ganz eindeutig. Die beiden Fälle, würden mir jetzt als erstes so einfallen.

I: Du hast es ja damit schon so ein bisschen gesagt, wie können denn sich so Belastungen im Unterricht äußern?

P: Ja, genau, also, ich nehme jetzt mal die beiden Kinder, von denen ich eben gesprochen habe. Der Junge, der wusste einfach gar nicht, wo er hier eigentlich so richtig war und was von ihm erwartet wird und hatte eine ganz große motorische Unruhe und war richtig blockiert im Lernen. Das ist einfach das richtige Wort. Also, er ist ohnehin extrem lernschwach. Aber eben durch diese Unruhe und dieses ganz sprunghafte so, ja, war es eben ganz schwierig, überhaupt einen Anfang zu machen im Lernen. Auch im Kontakt aufnehmen, sowohl die anderen Kinder, als auch ich. Das war alles ganz diffus und ganz schwierig. Und bei dem anderen Kind ist so deutlich zu merken, dass die gar nicht offen ist. Sie kann sich gar nicht auf irgendetwas konzentrieren. Sie kann eigentlich ganz viel. Das ist ein ganz schlaues Mädchen. Und trotzdem schafft sie ganz wenig und macht wenig Fortschritte. Hat deswegen auch wenig das Gefühl, auf irgendwas stolz sein zu können oder so. Weil sie (-). Manchmal sitzt sie eine Stunde da und macht gar nichts. Also, obwohl sie davorsitzt und ich mich auch zu ihr setze. Und dann vergräbt sie aber ihr Köpfchen nur und (-). Und dann wiederum, wenn sie zum Beispiel jetzt Pausen hatte, wo sie jemanden gefunden hat, mit dem sie gerne spielt, und sozusagen, gut drauf ist, (-). Das ist echt relativ bei ihr, muss man echt sagen. Aber, also für ihre Verhältnisse gut drauf ist, dann sehe ich auch immer, dass sie auch echt eine Menge kann.

I: Ist sie dann überhaupt ansprechbar, wenn sie so in sich gekehrt ist?

P: Ja, aber sie möchte nicht angesprochen werden. Das merkt man schon. Und sie empfindet dann alles als Druck und sie ist ganz (-). Ich habe den Eltern das auch im Elterngespräch gesagt, ich habe das Gefühl, die kann nicht mehr. Die kann einfach nicht mehr. Der Druck ist so groß für sie, auf anderer Ebene, dass das Thema jetzt Schule und Leistung in der Schule, das dringt gar nicht durch, durch

diese ganzen Milchglasplatten, die irgendwann die Sicht versperren.

I: Kann das auch einen Einfluss auf die anderen Schüler oder auf die Klasse haben?

P: Also, bei dem Jungen ist es so, dass es auf jeden Fall insofern Einfluss hatte, als dass der mir das erste halbe-dreiviertel Jahr nur durch die Klasse getigert ist, sehr laut war, sehr unruhig war und natürlich ganz viel Unruhe in die Klasse gebracht hat. Er ist ja nicht das einzige kleine Kind hier. Das wirkt sich aus auf die anderen und das ist insgesamt echt unruhig gewesen. Und ich sage es jetzt mal ganz offen, die Tage, wenn er mal nicht da war, das war so deutlich zu merken. Es war einfach deutlich ruhiger in der Klasse. Und bei dem Mädchen ist es so, das hat so eine Wechselwirkung. Sie ist eben ganz weinerlich, ganz empfindsam und empfindlich auch. Also, manchmal passieren gar nicht Sachen und sie fängt trotzdem schon an zu weinen, weil sie immer das Gefühl hat, sie hat ganz viel Unglück und ganz viel Pech und so. Es steckt einfach ganz viel Unglück in ihr. Und dann fangen die anderen natürlich an „wieso weint sie denn jetzt schon wieder?“. Und dann hat sie das Gefühl, „die anderen ärgern mich“ und das ist so eine ganz blöde Schraube, in die man da kommt. Also das bleibt nicht ohne Wirkung.

I: Welche Herausforderungen entstehen denn da für die Lehrkräfte in solchen Klassen?

P: Große Frage. Naja, es ist schon echt, das wird nicht langweilig, wenn du solche Kinder in der Klasse hast. Ja, echt, also, mit dem Jungen, du musst natürlich extrem differenzieren. Der konnte gar nicht, von Anfang an gar nicht an den gleichen Sachen arbeiten wie die anderen Kinder. Dadurch, dass er eben auch so extrem lernschwach war und gar keine Sprache hatte. Also, es ging noch nicht mal, dass

man irgendwie sagte „das muss jetzt grün ausgemalt werden oder rot“. Der hat fast ein Jahr gebraucht, bis er die Farben konnte und so. Also, es ist schon auch echt extrem. Also, man muss als Lehrkraft wahnsinnig differenzieren. Natürlich muss man trotzdem immer irgendwelche Elemente schaffen, die Gemeinsamkeit schaffen. Also bei allen, bei beiden jetzt auch. Also Verständnis, zum Beispiel, dass jemand andere Sachen nicht kann, herstellen. Dann, ja, auch versuchen, Kontakte herzustellen, zu sehen, wo passt das, wo können die in Kontakt treten mit anderen Kindern? Schon über die Sitzordnung, ich habe aber auch richtig Pausenverabredungen initiiert irgendwie. Das hat auch ganz gut gepasst. Aber natürlich findet es sich nachher dann auch von alleine immer mehr. Was habe ich noch gemacht? Natürlich hatte ich Elterngespräche. Klar, dann habe ich die Beratungslehrerin ins Boot geholt, Sonderpädagoginnen, weil es da eben jetzt auch einen sonderpädagogischen Schwerpunkt gibt. Ich habe im Falle des Jungen mich auch darum gekümmert, dass die eine Familienhilfe bekommen, weil die Eltern auch einfach überfordert waren mit dieser Problematik. Die haben drei Kinder und sprechen selbst die Sprache nicht. Die konnten ihren Sohn hier auch irgendwie gar nicht stützen, wie er es eigentlich brauchte. Vielleicht fällt mir im Laufe der Zeit noch ein bisschen mehr ein. Das ist das, was mir erst mal einfällt.

I: Ja, gerne sonst auch später noch. Gibt es denn Unterstützungsmöglichkeiten für Lehrkräfte, wenn sie mit psychisch belasteten Kindern umgehen?

P: Oh, da sprichst du echt was an. Ich finde, deutlich zu wenig. Ich habe mich bei Krankenhaus 1 gemeldet für den Jungen, weil es ja diese Ambulanz dort gibt. Und da bin ich auf die Warteliste gesetzt worden. Dann habe ich mich bei „XXX“ gemeldet, und habe vorher eine lange Liste bekommen, was ich alles vorweisen soll und gemacht haben soll und probiert haben soll, damit sich das für die

sozusagen lohnt, dass sie herkommen. Das habe ich dann alles abgearbeitet. Und dann kamen sie schließlich. Nach einem dreiviertel Jahr kam derjenige, der dafür zuständig ist. Und ich hatte das alles fein säuberlich sortiert. Das wollte er aber gar nicht sehen. Er wollte auch das Kind nicht ansehen. Er hat gesagt, das würde er im Erstkontakte erstmal gar nicht so stattfinden. Und hat mich auf eine weitere Reise geschickt. Ich soll also zum Kinderarzt gehen, da die Informationen einholen, XXXambulanz. Da waren noch verschiedene Sachen, die ich noch machen sollte. So, dass ich mich da echt, ehrlich gesagt, gar nicht ernst genommen fühlte. Und auch das Gefühl habe, bis das alles abgearbeitet ist, bis wir in der XXXambulanz zum Beispiel einen Termin haben, da ist der mit der Grundschule durch. Und das finde ich auch insgesamt ein Thema, wenn sie in die erste Klasse kommen, braucht man eigentlich sofort Hilfe. Ich hatte einen, der Junge hier ist mir komplett, der saß nie auf seinem Platz, ein anderes Kind ist immer als Feuerwehr, „didelidelit" durch den Klassenraum gegangen. Ich hatte echt gedacht, ich brauche jetzt hier Hilfe. Aber, bis die Kinder soweit diagnostiziert sind insgesamt, dass ich hier Hilfe bekomme, da sind die im dritten Schuljahr. Und das ist was, was ich auch echt auszusetzen habe. Weil ich denke, im Jahrgang eins, das ist ohnehin, das ist bekannt, das ist echt die anstrengendste Zeit. Und da muss man dann noch diese ganzen Diagnosen und all sowas machen, abwarten, bis man dann Hilfe bekommt. Das ist eigentlich echt nicht so günstig. Schon (unv. Wort) finde ich.

I: Ein bisschen spät dann. Was würde denn noch fehlen für, also, damit man besser mit psychisch belasteten Kindern umgehen kann? Oder, was würdest du dir wünschen?

P: Also, ich würde mir wünschen, dass den Kindern irgendwie mehr Aufmerksamkeit zu Teil werden kann. Ich kann das nicht leisten. Ich habe jetzt mittlerweile mehrere Kinder mit sonderpädagogischem Förderbedarf. Also, das

sind ja nicht meine einzigen beiden Fälle. Ich habe noch, es gibt noch Kinder mit ganz anderen Problemen und Auffälligkeiten so. Und so wie die beiden Aufmerksamkeit benötigen, das kann ich in so einem Klassenverband nicht leisten. Und das ist für sie das Schlimme. Das ihnen hier nicht geholfen wird. Und sie sind inzwischen so viel Zeit hier, verbringen so viel Zeit hier in der Schule. Dies Mädchen kommt meistens schon um halb acht, wird schon um halb acht gebracht und ist meistens bis um vier Uhr hier. Seit der ersten Klasse. Also, die verbringen richtig viel Lebenszeit hier. Und da ist so wenig Ressource für sie da. Das finde ich, das ist zu wenig. Das würde ich mir wünschen. Natürlich wäre es für mich auch eine Entlastung, aber natürlich geht es in erster Linie darum, was man für die Kinder irgendwie die optimalen Bedingungen schaffen will. Und das haben wir so in diesem System jetzt grade nicht. Wobei ich auch sagen muss, dass diese Klasse schon auch speziell ist. An anderen Schulen mag das anders sein, aber an dieser Schule ist das schon noch eine spezielle Klasse.

I: Welche speziellen Herausforderungen entstehen denn in interkulturellen Klassen? Also in Klassen, in denen ich eben Schüler verschiedener Herkünfte zusammen unterrichte.

P: Ja, verstehe ich schon. Da kann ich echt nicht so viel zu sagen, weil ich immer denke, dass können die natürlich viel besser beurteilen. Bei uns laufen sie einfach eigentlich so mit. Und jetzt, dass der Junge nicht so gut deutsch spricht oder so, das akzeptieren die Kinder. Darüber wird nicht gesprochen. Er kann oft spielen gehen, wenn die anderen nicht spielen gehen dürfen. Da fragt inzwischen auch keiner mehr, warum darf der das und warum darf ich das nicht. Ich habe noch zwei Mädchen aus Land 1 und aus Land 2, die arbeiten zum Teil in anderen Heften. Und auch das wird überhaupt nicht kommentiert oder erfragt oder so. Also, das finde ich, da sind die Kinder sehr offen. Das klappt gut.

I: Bedeutet das, gibt es da irgendwelche Herausforderungen für die Lehrkräfte?

P: Ich finde, also, ich habe das von Anfang an nicht so groß thematisiert, damit es eben auch kein Thema unbedingt werden muss, sie damit nicht so rausgestellt sind. Wenn es jetzt irgendwo wirklich Probleme gegeben hätte, oder irgendwelche Kommentare mir gezeigt hätten, dass da irgendetwas im Argen liegt, dann hätte ich mit Sicherheit darauf reagiert. Aber das war jetzt so gar nicht. Aber ich weiß es jetzt vielleicht auch nicht, ob sie was auf dem Schulhof erleben oder so, was ich nicht mitbekomme. Wäre gespannt, was sie später mal erzählen. Wie sie ihre Grundschulzeit wahrgenommen haben. Klar, deswegen sage ich, eigentlich müsste man sie das fragen. Aber aus meiner Sicht ist das eigentlich kein großes Thema für die anderen Kinder. Auf jeden Fall glaube ich wirklich nicht.

I: Entstehen denn vielleicht Ressourcen in interkulturellen Klassen?

P: Ja, finde ich schon. Also, erstmal finde ich es, unsere Gesellschaft ist in den letzten Jahren nun mal bunter geworden, und das ist eben, damit wachsen unsere Kinder jetzt hier auf. Ich habe kein Kind aus einem anderen Land in meiner Klasse gehabt, wie ich in der Grundschule war. Das finde ich, Schule soll ja aufs Leben vorbereiten. Das ist schon mal eine Vorbereitung. Dann erzählen die Kinder natürlich auch häufig. In Land 1war das bei uns so und so. Zum Beispiel, als es um Schule ging, Schule früher, da haben sie echt viel auch aus der Schule in Land 1 erzählt, so. Das fanden die anderen Kinder auch echt spannend. Ja., also ja, ich denke im Kern ist es doch mehr, dass sie schon von Anfang an so ein bisschen mehr Vielfalt auch erleben. Das ist hier jetzt ja auch schon echt eingeschränkt. Wenn man nur drei Kinder hat. Aber ja, immerhin.

…

Transkription des Interviews mit Teacher 6

Legende:

Interviewer=„I"; Proband=„P"

Pausen=(...)

unverständliches Wort=((unv.Wort))

Wort- und Satzabbrüche= (-)

Anonymisierungen=[…]

Teacher 6

I: Hast du noch irgendwelche Fragen, bevor wir starten?

P: Ne, wir können einfach (-).

I: O.k., wunderbar. Die erste Frage ist erstmal ganz allgemein, welche Erfahrungen hast du im Umgang mit Kindern mit psychischer Belastung?

P: Also, ich habe sie ja halt im Unterricht gehabt. Und habe halt einfach auch festgestellt, dass sie sehr viel mit anderen Dingen eigentlich beschäftigt sind, mit sich selber. Und sie eigentlich schwer Zugang finden, sich mit Lerninhalten auseinanderzusetzen, weil sie eigentlich gar nicht mental so dazu bereit sind. Weil sie ganz andere Dinge haben, die grade für sie viel dominanter, viel prägender sind in der Situation Schule. Auch, wenn sie vielleicht dann aus der Situation, die sie belastet, raus sind. Aber dann, ja da einfach dann so gar nicht den offenen Zugang dazu haben.

I: Gibt es noch andere Formen, wie sich diese Belastungen in der Klasse äußern können?

P: Naja klar, also ich sage mal nicht nur, dass sie selber halt eigentlich nicht in der Lage sind, zu lernen. Halten sie natürlich auch andere Kinder vom Lernen ab, indem sie halt irgendwie versuchen, (-). Oft ist das ja auch so eine Geschichte der Aufmerksamkeit oder der Beachtung, die ihnen halt oft sonst auch fehlt. Und dann sind sie natürlich auch dabei, dass sie halt versuchen, deine Aufmerksamkeit zu bekommen. Natürlich das auf jegliche Art und Weise versuchen. Erst machen sie es halt irgendwie vielleicht lieb und dann merken sie aber, ja o.k., wenn ich halt hier großartig positiv auffalle, dann bringt mir das gar nicht so viel. Dann versuche ich jetzt mal die Negativnummer. Das kommt halt leider häufiger doch dann auch vor. Und dann stören sie natürlich schon den kompletten Unterricht. Also da muss man schon ehrlich sein, dass das schon ein Problem

für die ganze Klasse dann auch ist. Von aufspringen, schreien, durch die Gegend rennen, unterm Tisch sitzen, laut weinen, Sachen durch die Gegend schmeißen, Hefte zerreißen, also, war alles schon dabei. Fliegende Locher, Schulranzen, Stühle, alles. Wenn dann da so ein wirklicher Wutausbruch kommt, dann ist da „Tabula rasa“. So, dass man dann auch selber zum Teil nicht mehr genau weiß, wie man denn die anderen Kinder schützen soll. Hatte ich häufiger mal so, die Situation. Auch nicht nur mit einem bestimmten Kind, sondern tatsächlich mit zwei, drei verschiedenen Schülern.

I: In einer Klasse dann?

P: Ja, also nicht unbedingt jetzt geballt auf einem Haufen, aber schon, dass dann immer mal der eine oder der andere dann mal austickte. Genau, dass man dann tatsächlich auch gucken musste, was mache ich jetzt? Ich stehe jetzt irgendwie dazwischen, die anderen Kinder zu schützen. Und das Kind auch vor sich selber zu schützen. Weil die dann teilweise so durchgedreht sind, dass sie tatsächlich sich dann auch selber, auch gefährden. Das sind natürlich die Extreme.

I: Wie wirkt sich denn sowas auf die anderen Kinder in der Klasse aus?

P: Ja also, die Auswirkungen waren natürlich, dass sie halt gesagt haben entweder, der oder die, ich hatte tatsächlich beide Geschlechter schon, ist halt einfach total furchtbar und die haben Angst vor dem oder ihr. Und wir wollen nicht mit der spielen. Oder ich kann überhaupt nicht lernen, das stört mich ganz doll. Aber natürlich auch andere Kinder, die dann vielleicht ähnliche Belastungen haben, die dann natürlich einfach auf den Zug aufspringen und dann halt sich dadurch ganz doll pushen lassen und dann halt mitziehen. Weil sie halt merken, der zieht jetzt alle Aufmerksamkeit in der

Situation, oder sie. Und für mich bleibt dann gar nichts mehr. Entweder, es potenziert sich, oder es ist halt so, dass andere Kinder halt sagen, sie distanzieren sich sehr davon. Und das zu Hause auch zum Beispiel erzählen. Also, ich hatte oft dann auch die Situation, dass viele Eltern kamen und Elternabende gar kein Gespräch mehr waren über das, was gelernt wird, sondern nur noch über das eine entsprechende Kind und was für Schwierigkeiten letztendlich für die Klasse mit sich bringt. Also, die anderen Kinder fühlten sich da doch schon sehr stark von gestört. Grade Ruhigere, sind da natürlich, dass die sagen „Um Himmels willen, was ist denn hier los“.

I: Was bedeutet das denn an Herausforderung für die Lehrkraft, wenn man solche Kinder in der Klasse hat?

P: Ja, das ist eigentlich ja total schwierig. Weil, du hast die Verpflichtung, denen etwas beizubringen. Den Bildungsauftrag, der dir irgendwo im Nacken sitzt und der dir natürlich ganz klar vorschreibt, was du bei den Schülern auch erreichen musst. Und wenn dann einer da durchdreht oder regelmäßig einfach nur unruhige Situationen in der Klasse herrschen, man eigentlich immer nur darauf bedacht ist, gewisse Schüler ruhig zu halten, dann bedeutet das natürlich, dass die anderen Kinder dafür keine Aufmerksamkeit oder keine Zeit bekommen. Was natürlich dann auch zu Lernrückständen führt. In meinen Augen ganz klar.

I: Bei der ganzen Klasse?

P: Bei der ganzen Klasse. Genau, tatsächlich bei der ganzen Klasse. Doch absolut, nicht nur bei dem betreffenden Kind. Obwohl ich auch schon einen Schüler hatte, der hat das fantastisch kompensiert. Also, der hat eigentlich den ganzen Vormittag nur gemacht, was er wollte. Aber war hervorragend in seinen Leistungen. Also, der war halt

einfach, hatte wahnsinniges Potential. Er war (-). Man möchte sich nicht überlegen, was der hätte machen können, wenn er es hätte ausschöpfen können.

I: Ist der denn dann am Ende, also nach einer gewissen Zeit, fügen die sich dann auch in die Klasse ein? Oder bleibt das (-).

P: Also nein, tatsächlich gar nicht. Er hat es immer irgendwie geschafft, dass er nicht grundsätzliche Ablehnung erfahren hat, tatsächlich von allen Kindern. Weil er ganz charming auch dann teilweise war, und grade so mit den Mädels, das echt gut hinbekommen hat. Und er ganz genau wusste, wie er wirkte. Also, er war ein ganz intelligenter Typ, der aber sehr die Situation für sich nutzen konnte, und sich dann aber manchmal einfach nicht im Griff hatte. Der aber auch ganz klar formulieren konnte, dass er dann irgendwann austickte. Der wurde irgendwann mit Medikamenten eingestellt, dann ging's. Und dann funktionierte das.

I: Wenn er das formulieren konnte, meinst du, dass er das selber sogar gemerkt hat, wenn er (-).

P: Ja zum Teil. Dass er halt sagen konnte, er hält das jetzt nicht aus, ob er mal fünf Minuten raus darf.

I: Ach so. Und das hat auch dann (-).

P: Das hat er aber erst gemacht, nachdem er tatsächlich dann eine Therapie auch bekommen hat. Dann hat er damit irgendwann angefangen.

I: Also das heißt, die Problematik blieb dann auch über die Jahre bestehen?

P: Blieb die ganze Zeit bestehen, ja tatsächlich. Weil häufig die Eltern ja nicht so dahinterstehen, nicht anerkennen, dass

es halt nicht funktioniert und das Ganze dann halt nicht unbedingt beschleunigen, dass es besser wird, sondern dann eher noch ausbremsen. Und eigentlich ihrem Kind dadurch gar keine Hilfe sind. Wobei es natürlich auch immer Gegenteile gibt, wo die Eltern sehr stark daran interessiert sind, weil es zu Hause vielleicht auch nicht geht. Aber sobald es zu Hause nicht so krass ist und nicht so sehr stört, habe ich das Gefühl, dass viele Eltern sagen, in der Schule, das kann ja nicht sein. Das sei dann unser Problem.

I: Gibt es denn da irgendwelche Unterstützungsmöglichkeiten für Lehrkräfte, wenn sie Kinder mit psychischen Belastungen in der Klasse haben?

P: Ich habe tatsächlich Gespräche mit den entsprechenden betreuenden Psychologen gehabt zum Teil. Das ist aber eher eine Sache, die man selber anschieben muss. Also, dass man meistens selbst so verzweifelt ist, dass man irgendwann sagt, so ich weiß da jetzt auch nicht mehr weiter, was ich machen soll. Dann hast du natürlich immer noch eine Sonderpädagogin an der Seite. Wo ich immer sehr gute Erfahrungen gemacht habe, die mich sehr, sehr doll unterstützt haben, grade mit solchen Kindern. Und die Klasse dadurch dann auch sehr stark entlastet wurde. Wenn sie dann einfach mal gekommen sind und dann gesagt haben „Komm ich nehme XY mit, und der/die darf sich dann noch zwei aussuchen, damit das nicht immer so eine gesonderte Geschichte ist. Und wir machen einfach was anderes, damit die anderen so ein bisschen vorwärtskommen. Und das muss ich sagen, hat alle total entlastet, hat aber auch den negativen Effekt, dass die Kinder das sehr stark bemerkt haben und auch formuliert haben. Wenn XY nicht da ist, dann ist das immer viel schöner. Oder morgens auch schon, „ja hier, sowieso ist nicht da“. „Oh, das ist ja gut so“. Und ich auch selber sagen muss, ich bin morgens teilweise zur Klasse gekommen, habe gesehen, Schüler ist nicht da, und war super

erleichtert. Weil ich einfach wusste, oh, wir haben mal einen entspannteren Tag. Und ich glaube, wenn mir das als Erwachsener schon so geht, wie schlimm muss das dann für einige Kinder sein, die da sehr sensibel auch für sind und da sehr stark auch drunter gelitten haben. Die dann da gar nicht irgendwie mit zurechtkamen. Ne, aber das fand ich sehr unterstützend mit der Sonderpädagogin zusammen oder auch mit der Psychologin. Dann XXX, fand ich auch super, habe ich auch total gute Erfahrungen gemacht. Die haben da sofort eigentlich agiert, haben sich da toll gekümmert und waren da wirklich sehr, ja sehr firm auch, indem, was man da auch tun kann. Die kamen dann auch schnell in die Klasse, haben sich das entsprechende Kind angeschaut und einfach mal geguckt, was ist da los. Wenn sie dann gemerkt haben, so eigentlich zeigt das Kind jetzt grade gar nicht das Verhalten, das mir da so beschrieben wird, dann kamen sie auch nochmal. Das war überhaupt kein Thema. Das fand ich immer wirklich richtig gut. Und die haben sich auch Zeit genommen, mit mir nochmal zu sprechen, mit den Eltern zu sprechen, gemeinsame Gespräche. Und ich sag mal, wenn es dann ganz eskalierte, dann war XXX zum Beispiel auch eine gute Ansprechstelle, wo ich immer gute Erfahrungen gemacht habe. Genau, das fand ich auch super. Da habe ich auch gute Erfahrungen mitgemacht. Die waren auch sehr professionell. Also haben nur gesagt, „ja hier, soundso sieht es aus. O.k., beschreiben Sie mal die Situation. Ich darf Ihnen nichts sagen, aber Sie können sich darauf verlassen, Sie werden wissen, dass wir was tun.“ Also dementsprechend fand ich, hat die Zusammenarbeit da auch sehr gut geklappt und hat mich sehr stark entlastet. Weil, zum Beispiel so Situationen vor Wochenenden waren für mich dann natürlich super. Zu sagen, o.k. ich kann das Kind jetzt so nicht nach Hause lassen. Das war schon echt gut. Genau. Im Unterrichtsalltag hat es das manchmal nicht unbedingt erleichtert, weil es die Kinder oft in eine Zwickmühle gebracht hat. Sobald diese Institutionen da reingekommen sind, weil sie dann zwischen ihren Eltern

standen und zwischen diesen Unterstützungssituationen, zwischen mir und die Eltern dann ja oft auch schlecht über die Lehrkräfte geredet hätten. Wir wären daran Schuld und wir hätten da angerufen und solche Sachen. Das hat die Kinder dann oft in irgendwie doofe Situationen geführt. Was den Unterricht auch wieder ein bisschen schwierig gemacht hat, weil sie dann so sich gar nicht zu verhalten wussten. Weil eigentlich, ich hatte immer ein gutes Verhältnis zu den Kindern. Aber die wussten dann nicht, ob ich sie noch mag. So also, stimmt natürlich nicht, dass hat ja für mich gar nichts gemacht. Aber das war noch so ein bisschen was, was noch so mit hinten reinspielte.

I: Gibt es denn irgendetwas, was du dir gewünscht hättest oder was du dir wünschst, was es noch an Unterstützungsmöglichkeiten gibt.

P: Ja also, ich sage mal, für uns an der Schule, da gibt es so eine XXX Gruppe. Sagt dir noch nichts? […] Und ich hatte auch vier verschiedene Kinder in unterschiedlichen Klassen, die auch diese Gruppe besucht haben. Das hat mir total geholfen. Weil das drei Sozialpädagogen waren, die halt wirklich sehr intensiv mit fünf bis sechs Kindern da gearbeitet haben. Und die Kinder sind da sehr gerne hingegangen. Das war toll. Das wäre vielleicht noch, was unterstützend war. Zusätzlich waren die dann auch immer noch mal in meinem Unterricht und haben das ihnen zugeteilte Kind dann auch im Unterricht noch ein bisschen begleitet. Aber da hätte ich mir gewünscht, dass man sowas kontinuierlich gehabt hätte. Und Schulbegleitung wäre da natürlich eine Möglichkeit. Eine Schulbegleitung hatte ich auch. Aber da muss ich leider sagen, das hat mir überhaupt nicht geholfen. Weil die Ausbildung dieser Leute ja einfach doch zu, gar nicht vorhanden ist.

I: War das dann so ein (-)?

P: Bufdi? Nein, ich weiß es ehrlich gesagt nicht. Das war einfach so ein Typ, der sein Studium abgebrochen hatte, irgendwie nichts gebacken gekriegt hat, und sein Kumpel meinte, mach doch Schulbegleiter. Und genauso war der auch.

I: Die haben so sechs Wochen eine Ausbildung oder sowas?

P: Wenn überhaupt. Ich muss sagen, das war für mich eher eine Belastung als eine Entlastung. Ich fand das nicht so schön.

I: Diese XXX Klassen, was genau heißt das?

P: Wenn ich dir das sagen könnte. Ich muss gestehen, da müsste ich jetzt auch nochmal nachgucken. […]

I: Aber das heißt, die werden da einfach dann aus den Klassen rausgenommen und separat praktisch unterrichtet.

P. Ja genau. Also, die haben jeden Tag zwei Stunden in dieser Gruppe, in der sie dann gemeinsam Spiele spielen, über ihre Gefühle sprechen und besprechen, wie geht es mir heute. Warum geht es mir so, wie es mir geht. Woran kann ich das denn bei jemand anderem erkennen, dass es dem nicht gut geht oder was würde ich mir wünschen, wie man sich mir gegenüber verhält, wenn es mir so und so geht. Genau, solche Sachen haben die dann tatsächlich da besprochen, und die durften dann da auch immer mal ein anderes Kind mit hinnehmen. Die haben dann auch erzählt, was die da machen und haben dann auch mal so eine Stunde mit der Klasse zusammen gemacht, damit die auch mal mitbekommen, was da läuft. Weil die anderen natürlich oft neidisch waren, dass sie da auch mal hinwollten. Weil sie natürlich nicht verstanden haben, was das da dann eigentlich ist. Ja und da waren natürlich auch Kinder, die zum Beispiel auch total still waren. Die sich ganz anders

gezeigt haben. Also, die nicht jetzt so laut und vielleicht auch gewalttätig waren, sondern die so ganz in sich gekehrt waren und ganz traurig eigentlich in sich. Also, das war so eine Mischung aus allem. Das war schon eine tolle Sache. Ja und wenn die nicht in dieser Gruppe waren, dann waren die entsprechenden Sozialpädagogen, die hatten immer zwei Kinder, für die sie zuständig waren, waren dann noch mit ein bestimmtes Stundenkontingent in der Klasse und haben versucht, die Kinder noch ein bisschen mental zu unterstützen, dass sie ein bisschen ins lernen kommen konnten. Ich glaube, eine sehr kostenintensive Sache, die nicht jede Schule irgendwie so aufrechterhalten kann. Ich weiß aber auch nicht, wie man darankommt.

I: Habe ich sonst auch noch nie gehört. Aber das klingt spannend.

P: Es ist eine tolle Sache, muss ich sagen. Steht und fällt natürlich auch ein bisschen mit den Leuten, die man da hat. Aber ich hatte zwei verschiedene Sozialpädagogen, die mich da unterstützt haben mit den Kindern, die waren super. Die haben einen ganz tollen Job gemacht einfach. Die Kinder hatten auch eine gute Bindung da zu denen. Die sind in den Pausen dann noch oft zu denen gegangen. Oder wenn sie ein Problem hatten, das war sehr schön. Und für mich auch, so ein bisschen, dass ich ein bisschen raus war aus der Geschichte. Weil man sich da emotional auch oft sehr da mit einbinden lässt. Man das zwar mit der Sonderpädagogin auch so ein bisschen teilen kann, aber das dann ja auch oft auf sehr fachlicher Ebene ablaufen muss. Und man das alles irgendwie in seine Schubladen und seine Papiere packen muss. Und dann konnte man das an der Stelle einfach auch mal so persönlich loswerden. Das war auf jeden Fall für mich entlastend.

I: Gibt es noch irgendetwas, was noch fehlt oder was noch helfen würde?

P: Ja, was fehlt? Ich glaube, das fängt schon ganz früh an, dass so was entsteht. Ich glaube, man müsste versuchen, dass man, was ja auch schon gemacht wird, dass diese Kinder frühzeitig in den Kindergarten kommen. Dass die frühzeitig auch irgendjemanden haben, der da von außen noch einmal einen Blick draufwirft. Jetzt gar nicht irgendwie, um sie vorzuverurteilen, weil sie irgendwie in einer bestimmten Ecke wohnen oder so was, sondern einfach nur um zu schauen, geht es denen wirklich psychisch auch gut. Dass man da früh interveniert. Weil, eigentlich ist es, wenn sie in die Schule kommen viel zu spät. Dann eskaliert das eigentlich nur noch komplett. Aber ihnen kann es vorher ja auch schon jahrelang nicht gutgegangen sein damit. Sonst würden sie sich ja nicht so zeigen. Also, das sehe ich eigentlich schon als eine große Chance, dass das viel früher eigentlich thematisiert werden müsste oder den Familien geholfen werden müsste. Weil, wir können es in der Schule nicht auffangen. Also ich glaube, eigentlich muss man das in den Familien schon so ein bisschen kompensieren oder anleiten. Den Eltern vielleicht auch so ein bisschen Hilfestellung geben. Wobei das natürlich auch wieder die Frage ist. Man kann niemanden dazu zwingen. Und ob man es dann annehmen mag ist ja dann auch die Frage, wenn man es halt irgendwie (-). Meistens sind es ja eh die, die sagen, so ich habe da Interesse dran, die dann sowas gerne in Anspruch nehmen. Und alle, die nur sagen „ja“, da ist das vielleicht jetzt nicht ganz so (-). Die möchten es dann eh nicht so gerne.

I: Das stimmt.

P: Ja, ich glaube, da in dem Bereich müsste man ein bisschen anfangen. Und in der Schule, ja ich glaube tatsächlich, Schulbegleitungen, die auch so ein bisschen so einen psychosozialen Hintergrund haben und auch wissen, was da bei den Kindern abgeht, die wären eine echte Hilfe.

Absolut. Die müssen ja vom Unterricht fachlich, also das glaube ich, kann jeder, der mal eine Grundschule selber besucht hat ganz gut irgendwie noch geistig bewältigen. Aber es wäre einfach schön, wenn dann jemand da ist, der den Kindern auch so ein bisschen Struktur durch den Tag gibt. Wir versuchen zwar, immer das gleiche jeden Tag irgendwie so ungefähr zu machen. Damit sie genau wissen was dann kommt. Das hilft ihnen auf jeden Fall. Aber oft ist dann auch mal ein Lehrerwechsel, dann ist mal ein Fachlehrer da, und dann eskalieren diese Situationen meistens. Oder es war eine große Pause, da gab es einen Streit. Die sind nicht in der Lage, den alleine zu klären. Dann bräuchte man eigentlich eine Stunde Zeit, um das aufzufangen. Dann ist man selber nicht da und dann hat man nach drei Stunden einen totalen Trümmerhaufen aus allen Kindern, weil dann keiner mehr glücklich und zufrieden ist und jeder doch was damit zu tun hatte. Von daher wäre es ganz gut, wenn man da jemanden hätte, der so ein Kind eigentlich von morgens, am besten zu Hause schon abholt. Weil es vor acht schon knallt auf dem Schulhof und es dann durch den Tag begleitet und einfach da ist. Muss ja nicht die ganze Zeit mit dem zusammen irgendwie spielen, um Himmels Willen. Soll ja auch mit anderen Kindern zusammen sein. Aber immer ansprechbar einfach zu sein. Das wäre schön. Schwer leistbar. Sehr teuer leider.

I: Ich würde jetzt einmal auf interkulturelle Klassen zu sprechen kommen. Was entstehen denn für spezielle Herausforderungen in interkulturellen Klassen? Also in Klassen, in denen eben Kinder verschiedener Herkunft gemeinsam unterrichtet werden?

P: Ja ich glaube, die Herkunft ist da nochmal, welche Herkunft muss man leider ein bisschen sagen. Ich glaube, dass muss man ein bisschen differenzieren. Weil ich das Gefühl habe, dass das eigentlich den Kindern völlig egal ist, wer woher kommt. Aber ich glaube, die Religionsgeschichte

spielt halt eine wahnsinnig große Rolle. Also es ist den Kindern total wurscht, ob da jetzt einer irgendwie asiatisch, afrikanisch, indisch aussieht. Aber es ist immer wieder so dieses, gut, jetzt vielleicht auch speziell in Stadtteil 1, feiert ihr Weihnachten, oder Nikolaus gibt es ja gar nicht. So, oder ja, das ist ein Nationalität 1. So, dann ist es auf einmal dann doch wieder was, was in Ordnung ist und legitimiert. Oder diese Essensgeschichte ist ein Problem. Und da irgendwie das Verständnis füreinander irgendwie zu bekommen, das ist, glaube ich, ein echtes Problem. Das ist wirklich schwierig. Weil die Familien da teilweise auch so manifest sind in ihren Einstellungen und ihren Werten, die sie ihren Kindern weitergeben. Und die XXX, die da so auf der Ecke sind, die sind, glaube ich, auch, das ist jetzt vielleicht auch weit aus dem Fenster gelehnt, relativ konservativ, um das mal so zu sagen. Die den Kindern einfach Werte vorleben, mit denen wir in Konflikt geraten tagsüber in den Schulen. Wenn sie eben grade so ein freies, lockeres Leben von deutschen Mitschülern zum Beispiel sehen oder ich würde auch sagen, andere westeuropäische Kids werden ja ähnlich groß. Die haben eine andere Sprache, aber die haben ähnliches, ja Werte und Religionsverständnis. Und ich glaube, dass ist der Knackpunkt leider. Das muss kein Problem sein, aber ich habe häufig schon das Gefühl, dass das ein Problem ist. Also, die Nationalität 1 Kinder, die halt total wohlsituiert da irgendwie waren, wo sich alle toll gekümmert haben, wo alles überhaupt kein Problem war so, die haben dann oft auf die „assigen Deutschen“ geschimpft. Die hatten ein ganz schlimmes Bild von „den Deutschen“. Die sind ja alle so leicht asozial. Die sind ja auch alle so offen mit ihrem Beziehungsleben. Und die knutschen auf der Straße. Das sind auch so Sachen, die deren Eltern ihnen wahrscheinlich auch erzählen. Und ich habe das Gefühl, das macht den Graben noch, noch tiefer. Und das macht das Lernen zwar manchmal interessant, aber das macht es auch unglaublich schwer. Weil immer wieder Konflikte entstehen. Wenn man das Gespräch mit denen hat. Ja, soundso viele

deutsche Kinder, es war irgendeine Statistik, fahren gerne Roller. So, dann sagt der Erste: ja ich bin ja kein Deutscher, ich bin ja Nationalität 1. Und dann sagst du ihm. „Aber du bist doch in Deutschland geboren, du hast doch einen deutschen Pass“. „Ne, ich bin Nationalität 1. Meine Mama sagt, ich bin Nationalität 1“. So dass irgendwie, das man sich da fragt „hm“. Also, wenn der selber offensichtlich von sich sagt, er gehört irgendwie dann doch nicht dazu oder irgendwie schon. Er weiß vielleicht selber gar nicht so genau, wo er eigentlich hingehört. Oder in dieser Stadtteil 1-Community schon, weil da ist es bei ganz vielen so. Und dann gibt es da den Deutschen. Der Deutsche ist furchtbar. Das ist irgendwie total interessant, weil es so viele Reibungspunkte einfach gibt, die viel zum Lernen beitragen können. Die aber auch wirklich viel, viel Geduld bedürfen. Weil viele da auch wirklich so bedingungslos erzogen werden, dass sie das nicht tolerieren können. Und so, diese Salami zum Beispiel auf dem Brot oder so, immer ein riesen Thema. Bis man das raus hat. In jeder Frühstückspause „Ih, du isst Salami. Du bist haram“. Wo man sich denkt, was ist dieses Wort? Brauch man ja dann so ein bisschen. Das, ja das ist schon schwierig. Das macht es den Kindern auch unnötig schwer. Das hat Potential, aber auf der anderen Seite auch eigentlich wirklich schwer.

I: Was bedeutet das denn für Lehrkräfte dann in diesen Klassen? Welche Herausforderungen entstehen da?

P: Na, dass die sich ganz besonders überlegen müssen, wie sie manche Themen auch unterrichten. Also ich würde jetzt mal so als, naja so als das Top 1 Thema, was problematisch ist Sexualerziehung zum Beispiel. Das ist ein riesen Problem für die Eltern. Die wollen partout nicht, dass ihre Kinder daran teilnehmen. Die sind dann krank und die dürfen dann nicht kommen. Und die Mädchen dürfen nichts von den Jungen wissen und die Jungen nicht von den Mädchen. Und, also ein riesen Ding. Und die Kids sind so interessiert daran.

Dass man den Eltern auch sagen kann „Wissen Sie, die klären sich hier auf dem Schulhof gegenseitig auf“. Und dann erzählen sie sich nämlich Quatsch. Und dann entstehen irgendwelche Sachen, die total unwahr sind. Lassen Sie sie doch lieber daran teilnehmen. Und dann zeigt man ihnen die ganzen Materialien, da fallen einigen Leuten die Augen aus. Da haben die totale Probleme mit. Sie sagen, das darf man nicht. Das wäre so ein Persönlichkeitsrecht, was verletzt wird. Und das ist tatsächlich schwierig. Da muss man ganz sensibel irgendwie mit vorgehen. Und man erreicht auch nicht jeden. Das stimmt schon. Und schon im Unterricht ist tatsächlich auch ein Problem. Da sind auch dann auffällig viele Mädchen häufig an dem entsprechenden Tag dann krank. Oder sie kommen nicht.

I: Das geht dann aber von den Eltern aus eher.

P. Ja ich denke das. Die Mädels würden das niemals sagen, dass sie nicht kommen dürfen oder so. Da bin ich mir sicher. Also ich glaube, viele leiden da auch drunter. (unv. Wort). Dass sie dann einen Mittelweg finden und dann sagen, gut dann gibt es einen Burkini, hatten wir schon mal einen Fall. Das Mädchen durfte dann immer mitschwimmen. Aber in den meisten Fällen ist es tatsächlich eher so, dass sie dann nicht kommen, traurig aber, an den beiden Punkten zeigt sich das ganz deutlich, wie schwierig das ist. Aber ist auch wieder ein Religion 2 Ding. Das kann ich überhaupt nicht sagen, was bei (-). Obwohl, das ist auch wieder nicht richtig. Weil ich auch zum Beispiel viele Nationalität 2 auch in den Klassen habe, und die ja auch Religion 2 sind. Und bei denen ist das überhaupt kein Thema. Obwohl die Mütter da häufig auch Kopftücher tragen und religiös sind so, hatte ich da noch nie ein Thema, dass irgendein Kind nicht irgendetwas durfte. Also ein Mädchen, bei den Jungs ist das ja nicht so das Problem. Ja stimmt. Hat damit vielleicht auch

noch mal wieder so mit der speziellen Bevölkerungsgruppe zu tun.

I: Spannend. Welche Ressourcen entstehen denn in diesen interkulturellen Klassen?

P: Ja also, ich muss sagen, ich habe durch die Eltern eine ganz große Ressource gehabt. […]. Und das ist so gut angekommen, dass die Eltern immer gefragt haben „Wann treffen wir uns wieder?“ Und dann habe ich es tatsächlich einmal im Monat gemacht. Es waren auch alle Papas eingeladen. Es sind eigentlich immer nur die Mamas gekommen. Und die haben da gebacken und gekocht und Essen mitgebracht. Und wir haben total nett uns unterhalten. Und die eine hat die andere zwar nicht verstanden, aber es war irgendwie egal, weil zwei hatten irgendwie Borek gemacht. Und dann „Wie hast du das gemacht?“ „Ja, wie hast du das gemacht?“ Und dann hat die eine übersetzt für die andere. Und irgendwie haben sie sich dann total nett ausgetauscht. Und das hat irgendwie so ein bisschen dazu geführt, dass so ein bisschen auch zwischen den Kindern (-). Weil, unsere Eltern verstehen sich ja. Wir sind uns gar nicht so unähnlich. Oder die sind in Ordnung. Dann da auch wieder total eine Akzeptanz irgendwie gewachsen ist. Das war total schön. Also ich glaube, die Eltern sind sehr, sehr wichtig in diesem Bereich. Die auch gerade mitzunehmen über was, dass sie halt gut können. Die sagen dann auch „aber ich kann nicht so gut sprechen“, oder „ich kann meinem Kind nicht helfen bei den Hausaufgaben“ oder so. „Das kriegen wir irgendwie nicht so hin.“ Und dann kann man mit anderen Eltern, die dann eine Idee haben, vielleicht dann irgendwas zusammen entwickeln. (unv. Wort). Dann kommt das gar nicht erst so weit. Das ist gar nicht gut. Ja und im Unterricht selber finde ich es eigentlich toll, dass die Kinder dann auch von ihren unterschiedlichen Festen und Bräuchen dann auch erzählen. Was hast du denn (-), Zuckerfest, was habt ihr da gemacht? Oder, wie macht ihr das denn an

Weihnachten? Oder, können wir nicht mal zusammen (-). Oder dass wir dann gesagt haben, o.k., wir feiern Weihnachten. Aber wir feiern dann auch mal Zuckerfest hier. Aber da müsst ihr mir sagen, wie feiert man das denn? Dann haben wir alle Eltern eingeladen und haben dann einfach gemeinsam so von jedem irgendwie so ein Stück mitgenommen. Das fanden irgendwie alle toll. Am Ende haben alle gemerkt, so o.k., ist ein anderer Tag, aber letzten Endes, so unterschiedlich war das gar nicht. Es ist irgendwie eine nette Party, alle kommen, es gibt Geschenke, alle haben gute Laune. So, ob das nun zu dem einen Tag oder dem anderen ist, das hat dann auch wieder so ein bisschen dazu beigetragen, dass man gemerkt hat, es ist eigentlich total albern zu sagen, dass wir so unterschiedlich sind. Eigentlich sind wir total gleich. Genau, das fand ich auch immer gut. Ja, was hat das noch für Vorteile? Es ist nie irgendwie langweilig.

I: Das glaube ich.

P: Ja, weiß ich nicht? Unterschiedliche Sprachen sind natürlich auch toll. Teilweise haben auch die Lehrbücher dann schon immer so Anregungen, dass dann noch mal so drinne steht irgendwie, unterschiedliche Sprachbeispiele sind, so ja „wie heißt das denn in deiner Sprache?“ Oder so. Dass man dann feststellt o.k., oh, das ist ja so ähnlich. Oder das ist so ähnlich wie das oder so ähnlich wie dieses. Wie schreibt man das denn in deiner Schrift, wenn du ein Nationalität 3 Kind dabeihast oder so was. Das ist schon schön. Dann mal da drüber zu sprechen. Das ist schon ganz gut. Aber es ist mehr so eine kulturelle Sache, als dass es jetzt wirklich einem Lerninhalt dienlich wäre, muss ich ehrlich sagen. Mehr so miteinander.

…

Transkription des Interviews mit Teacher 7

Legende:

Interviewer=„I"; Proband=„P"

Pausen=(...)

unverständliches Wort=((unv.Wort))

Wort- und Satzabbrüche= (-)

Anonymisierungen=[…]

Teacher 7

I: Welche Erfahrungen hast du im Umgang mit psychisch belasteten Kindern?

P: […] in jeder dieser Klassen hat es Kinder mit mehr oder minder starken psychischen Belastungen gegeben. Das waren an der XXXschule eher Kinder von Alkoholikern, auch Schwerstalkoholikern und ja auch immer wieder Kinder, die unter der Trennung ihrer Eltern sehr gelitten haben.

Ich habe ein Kind mit selektivem Mutismus (-) Ihre Eltern waren Nationalität 1, der Vater Alkoholiker, die Eltern waren dann später getrennt. Dieses Mädchen, die hat in der Schule nie gesprochen und sie war auch körperlich sehr eingeschränkt. Wenn wir einen Stuhlkreis gemacht haben, dann musste ich sie an die Hand nehmen und zu ihrem Platz führen. Von sich aus blieb sie völlig passiv. Ja, aber ich habe einmal, da hab ich dieses Mädchen einmal am Nachmittag auf einem Spielplatz mit Freundinnen Sprache1 sprechen hören, dass heißt sie konnte also sprechen.

Ich habe auch einen Jungen unterrichtet, der im Strafvollzug geboren wurde, weil seine Mutter dachte, wenn sie schwanger ist, ja also dass sie nicht einsitzen braucht. Der Junge ist bei seinen Großeltern aufgewachsen und er hat erst während der Grundschulzeit, also bei mir, erfahren, dass seine Großeltern nicht seine Eltern sind.

Und ich hatte eine Schülerin, bei der wurde ein Hydrocephalus diagnostiziert […]. Sie hat auch unter großen psychischen Störungen gelitten.

Ja also, ich habe Kinder von geistig behinderten Eltern unterrichtet, die selber schwer gestört waren.

Was noch?

Ich habe Kinder von kinderreichen Familien unterrichtet, die sehr unter der Armut gelitten haben und so psychisch belastet waren.

In den letzten Jahren habe ich tatsächlich auch vermehrt Schülerinnen und Schüler unterrichtet, die durch ihre Fluchterfahrungen und den, ja den, Kulturschock nur mit sehr großen Problemen am Unterricht teilnehmen konnten.

Ich hab dann häufig, ja habe ich erst im Laufe der Grundschulzeit gemerkt, warum ein Kinder besonders zurückgezogen oder auffällig oder aggressiv oder so ist und, dass da eben eine psychische Belastung zu Grunde liegt.

Ja, also ich glaube (-) ich der Meinung, dass es keinen Lehrer gibt, der nicht mit Kindern mit psychischen Belastungen arbeitet.

I: Wie äußern sich psychische Belastungen bei Kindern in der Klasse?

P: Nun ja, also ich gehe davon aus, dass ich als junge Lehrerin psychische Störungen nicht als Grund für unangepasstes Schülerverhalten gesehen habe. Dass da häufig tatsächlich eine Störung zu Grunde liegt ist mir erst später immer mehr bewusst geworden, als die Anzahl auffälliger Schüler so im letzten Jahrzehnt zugenommen hat. Ich würde sagen, die meisten Probleme haben die Schüler mit aggressivem und unruhigem Verhalten bereitet. Da hatten sich manche, die haben sich kaum unter Kontrolle, die können ihre Aggressionen und ihre Unruhe nicht steuern. Das bedeutet für den Unterricht natürlich permanente Störung. Solche Fälle sind selten, aber grade zu Beginn der Schulzeit gibt es immer wieder Kinder, die sich sehr schwer damit tun, sich in einen Klassenverband einzufügen. Bei manchen Schülern verstärken sich diese Symptome aber auch, je nachdem, wie die Situation in ihrem Umfeld ist. Wenn zum Beispiel das Leben in ihrer Familie, ja also wenn das problematischer wird, dann zeigt sich das natürlich im Unterricht. Und wenn dann Mitschüler und Lehrer auf das unangepasste Verhalten nicht richtig reagieren, (-) wobei was richtig ist, ist ja auch in jedem Fall anders. Also, wenn die nicht richtig reagieren, wird auch hier das Verhalten verstärkt. Solche Schüler waren in jedem ersten Schuljahr meine erste Baustelle.

Einige Schüler sind aber auch sehr in sich zurückgezogen. Die melden sich dann gar nicht oder nehmen kaum aktiv am

Unterricht teil, und auch auf Ansprache reagieren sie nur sehr ängstlich. Das stört den Ablauf des Unterrichts nicht. Deshalb, glaube ich, habe ich bei solchen Kindern immer erst sehr spät reagiert.

Manche psychische Störungen haben sich auch in motorischer Ungeschicklichkeit geäußert. Etwas ausschneiden oder anmalen, was ja schon in den Kindergärten geübt wird, hat einigen Schülern echt große Probleme. Ich erinnere mich da an Schüler, die keine Treppen hoch und schon gar nicht runter laufen konnten. Das muss nicht immer nur aus dem überbehütenden Verhalten der Mütter resultieren.

Ich erinnere mich auch zum Beispiel an besonders kleinkindhaftes Verhalten. Ich bin erst so im Laufe der Jahre darauf gekommen, dass auch bei extrem kleinkindhaftem Verhalten eine psychische Störung zu Grunde liegen kann.

I: Welche Herausforderungen stellt der Umgang mit belasteten Kindern im Klassenverband an die Lehrenden?

P: Ich glaube eigentlich (-) also ich finde die Arbeit mit psychisch belasteten Kindern gehört bei einem Grundschullehrer wie selbstverständlich zum Berufsbild. Das ist nicht immer einfach, man nimmt diese Belastung ja auch mit nach Hause. Und dann lebt die Familie, also die lebt den Schulalltag oft mit. Meine Tochter hat mal zu mir gesagt: Mama, du redest nur über die Schule. Das ist dann eben häufig wegen diesen besonderen Kindern. Bei mir jedenfalls. Meine jungen Kolleginnen haben, ja also die haben sich glaube ich ganz bewusst darum bemüht, diese Schicksale nicht zu sehr persönlich an sich rankommen zu lassen.

Es gibt zum Beispiel auch häufig sprachliche Probleme, die uns Lehrern oft gar nicht bewusst werden. Auch Schüler, die relativ gut deutsch sprechen, verstehen dann Begriffe, die für uns vollkommen selbstverständlich sind nicht. Das muss man erkennen und auffangen.

Teacher 7

Ich würde auch sagen, die Arbeit mit den Eltern belasteter Kinder ist eine große, zeitaufwendige, aber absolut notwendige Zusatzaufgabe zum täglichen Unterrichtsgeschäft. Ohne die Zusammenarbeit mit den Eltern kann man ein Kind nicht auffangen. Da muss man echt einen vertrauensvollen Kontakt herstellen, Schwellenangst abbauen und dann gemeinsam mit den Eltern für das Kind Lösungen finden, das erfordert einfach viel Zeit und Fingerspitzengefühl. Da sind manchmal Hausbesuche notwendig, man lädt die Eltern in den Unterricht ein und muss auch ohne festgelegten Termin immer ein offenes Ohr haben.

Ich finde einfach, dass es wichtig ist, also für mich auf jeden Fall, dass sich die Kollegen einig sein müssen, wie man mit dem einzelnen Kind umgeht und arbeitet. Es darf nicht darum gehen, nur seine eigenen Kinder zu sehen, die Schüler aus der eigenen Klasse, Problem müssen objektiv angegangen werden. Das ist halt auch nicht immer einfach. Manchmal muss man sich dann auch eingestehen, dass einen ein Schüler mit all seinen Problemen überfordert, also dass man es eben nicht schafft. Ich habe das Gefühl, das ist besonders für junge Kollegen ein schmerzlicher Prozess.

I: Welchen Einfluss können diese Ausdrucksformen auf die Klasse und die anderen SchülerInnen haben?

P: Also es ist ja so, dass Kinder mit starken psychischen Belastungen oder mit Migrationshintergrund häufig zieldifferent zur übrigen Klasse so mit Tages- oder Wochenplänen unterrichtet werden (-) so, das bedeutet für den Klassenlehrer natürlich ein ständiges im Auge haben. Diese Schüler bündeln viel Aufmerksamkeit, die den übrigen, ja oft über 20 Schülern verloren geht.

Es wird eben immer viel Rücksichtnahme gefordert, ohne die aber auch ein gewinnbringender Unterrichtsverlauf auch nicht möglich wäre.

Mit tat es immer sehr leid, wenn die Stimmung in der Klasse schlecht war, obwohl nur ein einzelner Schüler sich nicht an Regeln gehalten hatte. In den meisten Fällen wird so ein Fehlverhalten zunächst mit der ganzen Klasse aufgearbeitet. Und besonders ängstliche Schüler leiden unter solchen Situationen.

I: Welche Möglichkeiten gibt es bereits, um für Lehrende den Unterricht mit psychisch belasteten Kindern zu erleichtern?

P: Ja, Hilfen gibt es genug, Praktikanten, Bufdis, I Helfer . . . Leider sind diese Helfer oft keine wirkliche Hilfe. Ich glaube, die Ausbildung dieser Hilfen ist in den meisten Fällen nicht ausreichend, um diese Aufgabe erfolgreich zu bewältigen. Man braucht ja viel Fingerspitzengefühl, um mit psychisch belasteten Kindern umzugehen.
Unsere ehemaligen Förderschulkollegen, die jetzt seit der Inklusion für leider nur wenige Stunden mit in den Klassen sind, die sind schon eine große Hilfe. Sie sehen diese Kinder noch mal aus einer anderen Sicht. Ihre Ausbildung ist spezieller auf Schüler mit psychischen Belastungen ausgerichtet.
Im Landkreis XY und die Stadt 1 gibt es das Projekt XXX. Das ist eine präventive und begleitende Förderung von Grundschülern im emotionalen und sozialen Bereich. XXX ist besonders in so wirklich schwierigen Fällen sehr hilfreich.
Die Zusammenarbeit mit den Behörden gestaltet sich oft schwer. Ich habe manchmal versucht, das Jugendamt einzuschalten, und dann wurde mir in manchen Fällen gesagt, sie seien nicht zuständig. In einem Fall habe ich die Schulpsychologin eingeschaltet. Ich hatte da irgendwie das Gefühl, sie arbeitet nach einem vorgegebenen Plan, der bei meinem Schüler nicht anzuwenden und wenig hilfreich war.
Viele Eltern suchen auch Hilfe bei Ergotherapeuten. Die versprechen den Eltern oft schnelle Hilfe, weil sie halt darauf angewiesen sind, dass die Eltern weiterhin zu ihnen kommen. Das Problem ist halt, wenn sich der Erfolg dann in

der Schule nicht einstellt, dann denken einigen Eltern, dann muss es wohl doch am Lehrer liegen.

I: Was fehlt noch für den erfolgreichen Umgang mit belasteten Kindern?

P: Also in meinen Augen wäre es eine echte Unterstützung, wenn sich die Sozialarbeiter an den Schulen um die Vernetzung mit dem Jugendamt, der Beratungsstelle oder dem Schulpsychologen kümmern würden. Wir müssen oft Telefonate in den Pausen führen, das macht den Schulalltag dann eben echt noch hektischer und anstrengender.
Und ich finde, statt der vielen unausgebildeten Helfer, sollte es in den Klassen (-) also eine Doppelbesetzung in den Klassen durch Lehrkräfte wäre ideal.
Man braucht einfach deutlich mehr Unterrichtszeit, um Probleme in Ruhe klären zu können, und um Präventionsprogramme selber durchführen zu können.
Professionelle Supervision zum Beispiel, die aber auch daran scheitert, dass man sowieso schon den ganzen Tag in der Schule verbringt.
Sprachunterricht für Kollegen. Ich glaube tatsächlich, dass junge Kollegen durchaus bereit wären, einen Basiskurs in Türkisch, Kurdisch etc. zu belegen.

I: Ich würde jetzt einmal auf interkulturelle Klassen zu sprechen kommen. Was entstehen denn für spezielle Herausforderungen in interkulturellen Klassen? Also in Klassen, in denen eben Kinder verschiedener Herkunft gemeinsam unterrichtet werden?

P: Andere Länder, andere Sitten und vor allem, andere Religionen, andere Sitten. Die Schüler und besonders deren Eltern aus anderen Ländern haben häufig Anpassungsschwierigkeiten. Sie erwarten dann, dass wir ihre Sitten oder religiösen Gepflogenheiten akzeptieren, lehnen aber unsere manchmal rigoros ab, zum Beispiel die

Ramadanzeit. Ich hab es oft erlebt, dass die Grundschüler, also die essen und trinken in dieser Zeit häufig tagsüber gar nicht, aber wenn ein kleines Kind den ganzen Tag über nichts isst und trinkt, kann es kaum gute Schulleistungen erbringen.

Wir hatten auch Flüchtlingskinder, die noch nie eine Schule besucht haben, vom Alter her aber schon in eine höhere Klasse müssen, aber (-) Sie können bei ihrer Größe auch nur schwer in einem ersten Schuljahr beschult werden.

Die Eltern ausländischer Kinder sind noch wieder eine besondere Herausforderung. Also, sie haben ihre eigenen Probleme damit, in Deutschland anzukommen. Aber sie wollen für ihr Kind natürlich auch nur das Beste. (-) dann kommt hier noch die Sprachbarrieren dazu.

Bei gebildeten Eltern von Flüchtlingskindern, die erwarten von ihren Kindern oft, dass sie das Gymnasium besuchen sollen, obwohl die Leistungen gar nicht Fall ausreichend sind. Sie wollen dann, für ihre Kinder eine gesicherte Zukunft, aber kennen die Anforderungen in unserem Schulsystem nicht richtig, ja.

I: Welche Herausforderungen entstehen für SchülerInnen in interkulturellen Klassen?

P: Also die Jungen der arabischen Länder werden ja häufig als Machos erzogen. Das ist dann so, Respekt müssen sie wohl vor ihrem Klassenlehrer oder der Schulleitung haben, aber Mitschülerinnen werden häufig nicht so wirklich geachtet. Ja, also das ist dann oft schwierig. Und die deutschen Mitschüler, die müssen dann so Schimpfwörter, wie „du Aleman", die müssen verkraftet werden.

I: Welche Ressourcen gibt es möglicherweise in diesen Klassen?

P: Naja, also ich sag mal neben ihren normalen Fähigkeiten, die jedes Kind mit in die Klasse bringt und der Klasse

dadurch gut tut, also in einer gut geführten Klasse, da müssen alle Kinder Rücksicht aufeinander nehmen. Es ist ja so, dass, es profitieren alle Mitschüler davon, dass sie lernen in einer Gruppe jeden so zu respektieren wie er ist.

Auch die Schwächsten und die ganz belasteten müssen von der Gemeinschaft akzeptiert werden. Aber ja, das kann man erreichen und hilft allen Schülern für ihr zukünftiges Leben.

Es ist ja so, dass viele Dinge, wie Gummibärchen, wegen der Gelatine, die verbietet die Religion muslimischer Kinder.

Aber hier lernen dann alle eben schon früh, dass solche Unterschiede in unseren Religionen und Kulturen eben auch kein Problem sein müssen. Ich habe es meistens so erlebt, dass die Kinder und auch die Eltern der anderen Kinder Rücksicht auf Besonderheiten nehmen, also so zum Beispiel, wenn sie an Geburtstagen Süßigkeiten mit in die Schule geben.

Also, bei einer engagierten Elternschaft nehmen alle Eltern zum Beispiel an der Vorbereitung von Klassen- oder Schulfesten teil. Das hilft sehr an der Basis dabei, dass wir ohne Vorbehalte miteinander umgehen.

…
