# Supplementary material for: Mental health and cultural and linguistic diversity as challenges in school? An interview study on the implications for students and teachers
Source: PLoS One. 2020 Jul 20;15(7):e0236160. doi: 10.1371/journal.pone.0236160 (PMC7371207; doi:10.1371/journal.pone.0236160)
Supplement: S3 Transcripts — (PDF) [file pone.0236160.s003.pdf]

S 3 Transcripts

Excerpts of the transcribed interviews with the teachers

Transkription des Interviews mit Student 1

Legende:

Interviewer=„I“; Proband=„P“

Pausen=(...)

unverständliches Wort=((unv.Wort))

Wort- und Satzabbrüche= (-)

- Anonymisierungen=[…]

I: OK, dann fangen wir mal an mit der ersten Frage. Gehst du denn gerne zur Schule?

P: Ja. Schule macht Spaß. Man lernt sehr viel und man macht da tolle Sachen, lustige Sachen. Ähm, manchmal gibt es aber auch so Sachen, welche, zum Beispiel im Sportunterricht, wenn man jetzt so ein Spiel spielt und die anderen wollen das nicht spielen. Dann gibt es da so, wie soll ich sagen, bisschen, keine Ahnung, Durcheinander. Weil 'n paar wollen das und die anderen wollen das und dann wollen man- sagen manche ja auch manchmal, wenn man jetzt auf einem Kindergeburtstag ist oder so, oder in der Schule was spielt, "ne das möchte ich nicht spielen. Ich spiel doch nicht mit, ich möchte mit jemand anderem spielen", oder so. Oder will was anderes spielen. Ist dann ja auch manchmal blöd, weil man streitet sich dann ja auch.

I: Ja. Hast du denn Freunde bei dir an der Schule?

P: Ja, glaub 10 neue Freundinnen habe ich kennengelernt.

I: OK, stimmt, du hast gerade die Schule gewechselt, ne?

P: Ja, 7 sind das glaub ich. Ich lad jetzt zu meinem Geburtstag 17 Kinder ein.

I: Oh hah (lacht). Das ist 'ne große Gruppe.

P: Ja, ich hab XXX Geburtstag […].

I: XXX, genau. Also noch XXX Tage, oh hah, das ist ja bald. Gibt es manchmal Momente, in denen du dich nicht so gut fühlst?

P: Ähm.

I: Traurig oder wütend bist oder so?

P: Hm ne, fällt mir jetzt grad irgendwie nichts ein.

I: Ja, muss auch jetzt nicht nur in der Schule sein, vielleicht zu Hause oder so.

P: Ja, wenn manchmal, ähm, wenn Kaninchen oder Hühner von mir gestorben sind, bin ich öfters mal traurig. Oder wenn mein Bruder mich manchmal ärgert. Oder irgendwas kaputt gegangen ist von mir. Oder was ich meiner Mutter irgendwie schenken wollte zum Geburtstag oder so und das ist kaputt gegangen, da bin ich dann auch traurig oder wütend oder so.

I: Und was machst du dann, wenn du traurig oder wütend bist?

P: Ähm, wenn ich wütend bin, dann geh ich in mein Zimmer und beruhig mich wieder, mal dann irgendwas. Oder wenn ich traurig bin, geh ich nach draußen, atme tief Luft ein und so und erhol mich erstmal wieder, damit ich das- das wieder weg geht und damit ich nicht traurig bleibe und ja-

I: OK und können and're Menschen vielleicht sehen, also könnte ich sehen, wenn es dir nicht gut geht? Würde ich das mitkriegen?

P: Hm, ja wenn man weint, dann weiß man ja einer ist traurig. Aber wenn der eine auch nur so ganz leise-, so ganz traurig guckt. Ich seh das manchmal beim Mitschüler. Da guckt er dann immer so traurig und ja. Dann sagt er immer, was hast du und dann sagt er immer: "Nichts". Da hab ich das Gefühl, die haben doch irgendwas.

I: Ja, und würden deine Mitschüler merken, wenn es dir nicht gut geht? Oder deine Freunde?

P: Hm, ja, manchmal schon, wenn ich so traurig gucke oder so. Und ich nichts davon erzählen möchte oder manchmal erzähl ich davon auch was, aber ich versuch dann auch wieder versuchen zu lachen. Und, aber, ja, manchmal fragt mich dann auch jemand und dann sag ich's halt oder ich lass es einfach, wenn's zu traurig ist.

I: OK, ein bisschen hast du es ja schon gesagt. Was hilft dir denn, damit es dir dann wieder besser geht?

P: Wenn man Spiele mit mir spielt oder so. Das mag ich ganz gerne, zum Beispiel, Monopoly, Mensch ärger dich nicht, ich muss mich dann immer totlachen, wenn der andere gewonnen hat und das ist dann immer so blöd. Oder wenn ich und meine Freundinnen uns Witze erzählen, oder, ähm, wenn wir draußen im Garten spielen, mit meinen Kaninchen kuscheln und so, dann fühl ich mich auch manchmal wieder besser.

I: Ja, das glaube ich. Die nächste Frage ist, wie deine Freund oder deine Klasse dir helfen können, wenn es dir mal nicht so gut geht.

P: Hm, trösten würde ich dazu sagen. Vielleicht mal kurz raus gehen, mit 'n Vertrauten, mit der Freundin oder 'nem Freund. Dann mit denen reden, und aber das bleibt dann unter denen. Und der sagt das dann dem Lehrer, wenn man sich jetzt nicht traut, zum Beispiel 'ne Vase umgeschmissen hat, aus der Schule, die da jetzt so stand und das gehörte der Schuldirektorin oder so. Und man hat das ausversehen kaputt gemacht und man ist traurig darüber. Und man traut sich jetzt nichts zu sagen. Dann redet man mit 'nem Freund draußen. Und der sagt das dann zum Beispiel zum Lehrer oder so.

I: Mh, ja, und könnten deine Lehrer oder deine Lehrerin dir auch irgendwie helfen, wenn es dir mal nicht gut geht?

P: Vielleicht auch mal so reden oder so. Das macht unser, machen unsere Lehrer auch. Da hatten wir letztens so 'n Kind in der Klasse und der hat dann auch geweint und da hat der Lehrer dann gefragt "was ist? Sollen wir mal kurz raus gehen und alleine reden?". Das machen die dann öfters. Oder wenn die so private Fragen haben oder so, schreien die das dann ja nicht laut durch die Klasse, sondern gehen dann raus oder so.

I: Ja. OK. Du hattest ja gerade schon gesagt, dass manchmal Mitschüler von dir auch traurig gucken. Wie siehst du denn bei deinen Freunden, wenn's denen vielleicht nicht gut geht? Erkennst du das irgendwie?

P: Meine Freundinnen, meine eine, da denk ich immer die weint, aber dabei kommen einfach nur so Tränen, weil sie immer so lachen muss oder so. Aber manchmal da werden die Augen rot und dann merke ich, "aha, jetzt fängt sie gleich an zu weinen. Geh mal schnell hin und geh trösten". Und bei den anderen, bei den neuen Mitschülerinnen und Schülern hab ich das noch nicht gesehen. Und, aber, bei meinem Bruder seh' ich das auch manchmal, dass er traurig ist, oder meine Mutter oder mein Vater, dass sie dann so ganz leicht traurig gucken. Ja, und dann frag ich immer, "was ist? Ist alles gut?" Und "soll ich dir irgendwie helfen? Soll ich irgendwas machen?" Oder so.

I: Ja, guck mal, jetzt hast du es auch schon so ein bisschen gesagt. Was machst du denn, wenn es, damit es deinen Freunden wieder besser geht, wenn du merkst, denen geht's nicht so gut?

P: Ich hab die Frage nicht genau verstanden.

I: Wenn es deinen Freunden nicht so gut geht, was du dann versuchst, damit es ihnen wieder besser geht.

P: Ich versuch sie zum Lachen zu bringen, durch zu kitzeln oder so, oder Spiele mit denen zu spiele, oder, über was anderes nachzudenken, oder zum Beispiel, "wir könnten jetzt Monopoly spielen. Hast du da drauf Lust zu?" Wenn er da auch keine Lust zu hat, dann, oder zu gar nichts, dann tröste ich sie und frag "Ist alles in Ordnung? Willst du mit mir da alleine drüber reden. Ich erzähl's auch keinem weiter" Und dann weiß man ja auch manchmal 'ne Antwort oder dazu, was man dann machen könnte. Dann geht's der, oder derjenigen denn ja wieder besser.

I: Ja, und deine Lehrerin und Lehrer, was machen die, wenn es jetzt einem Schüler in der Klasse jetzt gerade nicht gut geht.

P: Die gehen dann zu ihm hin und fragen "Was ist los?" oder und so, sollen wir mal kurz raus gehen und alleine reden. Dann gehen die manchmal raus oder so. Oder, ja. Mehr weiß ich da jetzt auch nicht so genau.

I: Alles gut. OK, und was passiert denn so in der Klasse, wenn es da jetzt einem Schüler nicht gut geht. Hat das vielleicht auch einen Einfluss auf die ganze Klasse oder den Unterricht?

P: Also, dann gehen manchmal paar Schüler, stehen die dann auf, gehen hin und tröste die und fragen denn. Ja, dann bricht der Lehrer ja auch kurz den Unterricht ab, weil ja- weil er muss dann mit den Jungen reden, damit er nicht so die ganze Zeit rum heult und so. Oder die gehen kurz raus und beruhigen sich dann wieder. Das hat unsere Lehrerin letztens auch gemacht. Die hat gesagt "willst du kurz alleine raus gehen, dann kannst du dich ja wieder beruhigen und deine Tränen aus den Augen wischen.

…

I: Nicht schlimm. OK, da haben wir es auch schon geschafft mit den ganzen Fragen. Gibt's noch irgendwas, was du gerne sagen möchtest, was dir jetzt noch eingefallen ist. Was ich vergessen hab, oder so?

P: Ähm, nö, fällt mir gar keine, keine so richtigen Fragen jetzt so ein. Ne, eigentlich

I: Ist auch nicht schlimm.

P: Aber manchmal finde ich das n bisschen blöd, wenn zum Beispiel jemand sauer ist, wenn wir jetzt zum Beispiel Klassensprecherin oder Klassensprecher wählen, ne? Das zum Beispiel der eine sagt "wenn du mich nicht wählst, bist du nicht mehr mein Freund oder meine Freundin". Oder das ist ja auch so gemein. Oder so, oder zum Beispiel jetzt hat derjenige jemand anderen gewählt und da hat er gesagt "hast du mich eigentlich auch gewählt? Ich will jetzt nicht mehr dein Freund sein, weil du mich nicht mal gewählt hast".

I: Ja, das ist schwierig. Und redest du dann da mit der Lehrerin drüber oder wie macht ihr das?

P: Also, unsere Lehrerin hat gesagt "ihr müsst jetzt nicht euren Freund oder eure Freundin wählen, sondern den- oder diejenige, die ihr meint, die das Zeug dazu hat, auf andere Kinder Rücksicht zu nehmen" oder so, wenn die zum Beispiel n Anfall kriegen oder, also n Wutanfall oder sich prügeln. Das hatten wir schon mal bei uns in der Klasse, aber nicht in der neuen sondern in der alten Grundschule.

I: Und was ist dann passiert, wenn sich da Kinder im Unterricht prügeln?

P: Dann geht der Klassensprecher dazwischen und sagt "halt stopp, jetzt setzt ihr euch wieder hin und geht euch

einfach in der Pause auch auf'm Weg, aus'm Weg." Und da, wenn die Lehrerin dann ja weg ist, dann sagt der Klassensprecher "ja, hier, die haben sich grad geprügelt, aber ist jetzt ist alles wieder geklärt". Weil die müssen das dann ja erst klären und dann sagt die Lehrerin "Ok, dann ist ja alles gut, dann brauch ich das ja auch nicht mehr klären".

I: OK, und das klappt dann auch, wenn der Klassensprecher dazwischen geht?

P: Ja, bei uns in der alten Grundschule jetzt grad nicht so, weil da war so ein Junge dabei. Der hat sich da jetzt nicht so richtig dran gehalten.

I: Und was war dann in der Klasse, hat man das, habt ihr das alle so gemerkt, dass der sich nicht so ganz an die Regeln gehalten hat?

P: Hm, ja, weil der öfters mal so Stinkefinger gezeigt hat, oder so. Ja, das ist auch ganz blöd. Oder, den anderen geprügelt hat.

I: Ach, tatsächlich? Auch im Unterricht dann?

P: Ähm, ja, im Unterricht öfters als draußen. Und dann hat der schon mal einen Jungen zu Boden getreten und gehauen. Und da sind wir noch knapp dazwischen gegangen. Weil der kriegt immer Wutausfälle und so. Und deswegen haut der dann immer ab und da ist der letztens auch nach Hause abgehauen.

I: Hm, oh je. Und, was ist dann so im Unterricht? Habt ihr das dann im Unterricht besprochen? Oder, ist dann -

P: Also, wir haben dann so einen Erzählkreis gemacht und, dann was uns am Herzen liegt, was wir gerne sagen möchten. Dann durften wir auch das sagen, was wir jetzt

ehrlich meinen. Zum Beispiel: "Ja, dieser Junge zeigt jeden Tag immer Stinkefinger. Und das nervt 'n bisschen und das stört auch richtig". Und im Unterricht hört, passt der auch nie auf und deswegen musste unsere Lehrerin auch immer fast schimpfen, weil der nie aufgepasst hat. Die hat zu ihm auch immer gesagt, manchmal "ja, wenn du jetzt nicht aufpassen möchtest, dann geh nach draußen und mal da irgendwas, oder so". Weil der war das egal, sie wollte ja jetzt nicht mit einem, mit dem jetzt diskutieren die ganze Stunde. Sondern einfach gewähren lassen, sag ich jetzt mal. Damit der Unterricht ja auch weiter gehen kann.

I: Muss ja auch, ne. Ist ja sonst auch schwierig. Und habt ihr dann als Klasse auch geschafft, dass es ihm ein bisschen- dass er ein bisschen ruhiger wurde? Oder war es bis zum Ende dann schwierig mit ihm?

P: Also es war bis zum Ende eigentlich bisschen noch schwierig, aber sonst hat es sich eigentlich bisschen wieder verbessert. Aber ein bisschen auch nicht und ja. So gemischt halt. Manchmal an einem Tag war gar kein Streit am anderen Tag war richtig doll Streit und an den anderen ein bisschen. Immer so unterschiedlich ging das.

I: Ja, hat man gemerkt, wann das ein guter Tag wird und wann nicht. Konnte man ihm das anmerken oder-

P: Also ja, manchmal hatte der so gute Laune, wenn er zum Beispiel, war er irgendwie sauer oder so, weil er hat irgendwie seinen Schulranzen so auf die Bank geschmissen, weil wir haben so eine Bank, manche Klassen. Und da stellen die immer die Schulranzen drauf und er hat dann immer seinen- also er hat mal seinen Schulranzen so hingeschmissen, da wussten wir, äh "oh, heute wird's n blöder Tag, aber hoffentlich vielleicht auch nicht". Weil zum Beispiel bei Ausflügen, da haben die sich auch manchmal gestritten oder so. Aber manchmal haben die es auch

gelassen, weil die Lehrer dann auch gesagt haben, derjenige der sich prügelt, zum Beispiel zum Schwimmen, hatten wir immer 'ne Regel: Der sich prügelt vorher oder schlägt oder Schimpfwörter sagt, muss in eine andere Klasse gehen und da bleiben.

I: Ah ja, und das hat gewirkt.

P: Ja. Also bei uns in der Klasse jetzt nicht so, weil eigentlich gilte das auch, wenn man keine Mütze hatte, weil das ist ja auch, wenn es jetzt so ist, ohne Mütze dann draußen, dann ja, und dann hatten die meisten ja 'ne Kapuze aber das hat ja auch nicht ganz ge- gerichen und so. Aber deswegen hatte der Lehrer sich jetzt auch nicht so richtig dran gehalten. Also eigentlich ist noch gar kein-, doch, n paar Mal ist dieser Junge, der dauernd den Stinkefinger zeigt, auch paar Mal sitzen geblieben. Aber die anderen sonst nicht. Ich hätte dann selber zugegeben "ich hab keine Mütze mit" und ich geb das dann ja immer selber zu. Weil ich möchte dann ja immer gerne ehrlich sein, auch wenn ich jetzt selber Ärger gemacht hab, mache ich auch immer, bin ich auch ehrlich. "Ja, ich hab dies das gemacht, ausversehen. Ja, ich hab ihn ausversehen getreten" und sag dann nicht "ich war das nicht". Weil das passiert öfters bei uns in der Klasse, dass derjenige das sagt "ich war das nicht, ich war das nicht", nein der da hat hat, der da hat angefangen, nicht der. Der hat angefangen. Ja, und dann geht das immer so weiter, bis zum heftigen Streit wird und dann muss der Lehrer auch mal schimpfen. Unsere Lehrerin musste jetzt öfters mal schimpfen, in der neuen, also in der neu- äh in der neuen Klasse und weil die Jungs dauernd dazwischen gequasselt haben und ein Lehrer musste auch schon mal richtig schimpfen. Der ist ganz rot geworden. Der hat gesagt: "Jetzt habt ihr meine gute Laune weg gemacht und jetzt ist die schlecht geworden." Da war er auch stinkesauer.

I: Ja, das glaube ich. Und wie fühltest du dich dann so, wenn da so ein Streit in der Klasse ist?

P: Also, ich erschreck mich dann erstmal, zum Beispiel wenn derjenige jetzt richtig laut, dann spring ich immer richtig so richtig hoch und erschreck mich dann immer, wenn ich das sehe, dann geh ich dahin und versuch dann, zu helfen, dass derjenige den anderen zum Beispiel jetzt nicht schlägt oder so. Stell mich dann davor und dann trennen wir uns so zum Beispiel in der Grundschule haben wir das mal so gemacht: Der Klassensprecher, der eine ging mit der dahin in die Ecke und der andere Klassensprecher ging mit den anderen nach draußen oder so. Dann haben die da so gesprochen und so, so was los ist und "warum habt ihr euch gestritten?" und dann sagen die das zu mir auch.

I: Ja, ja, das habt ihr dann ja schon echt gut gemacht, wenn ihr da schon so Methoden hatten, wie ihr damit umgehen könnt, und die auseinander bringt.

P: Ja, wir hatten solche Regeln, da steht dran "wir gehen freundlich miteinander um. Und wir vertragen uns, wenn ein Streit jetzt zum Beispiel zwischen uns läuft, oder, da gibt’s auch so andere Regeln, wie, wie wir frühstücken am Platz und nicht irgendwie so durch die Klasse rennen, denn dabei noch Brot esse. Da kann man sich ja auch schnell bei verschlucken. Und die Jacken werden ordentlich aufgehängt und nicht auf 'n Boden geschmissen. Und dann macht man, soll man ja auch nicht mit Absicht machen.
Und die haben sich dann ja auch gestr- um die Haken gestritten. Ja, wo die Jacken dran sollen, das habe ich auch nicht so richtig verstanden. Weil, es gab keine eigenen Haken und das ist doch eigentlich egal wo man hängt.
Also mir war das völlig egal. Die anderen wollten nicht neben einem Jungen hängen, die anderen da nicht bla bla bla.
Dann haben aber manche auch reagiert und dann gesagt: "hört ihr jetzt auf zu streiten. Es ist doch egal, welcher Haken

es ist." Und da ist der eine Junge, denn ja so frech geworden, ist nach draußen gegangen. Und dann hat er 'n Wutanfall gekriegt, so.

Ja, (unv Wort: 20:14 Min.) und dann hat er letzt-, denn schlägt er manchmal, wenn er 'n Wutanfall hat und der Lehrer jetzt grad nicht da ist, schlagt er richtig so auf den Tisch und da ist letztens, hat er so mit der Faust richtig gemacht und da ist der Tisch einmal so gebrochen fast. Da ist so 'ne Ritze gekommen, da ist der Tisch fast durchgebrochen.

I: Oh je. Und was machen du und deine Freunde dann, wenn so was im Unterricht passiert?

P: Dann sagen wir "beruhige dich" und, "was ist los, warum bist du s- warum hast du jetzt n Wutanfall gekriegt? Was ist passiert?" Oder wenn der Lehrer dabei gewesen ist, sagt er – hier, hat unser Lehrer gesagt "was ist passiert? Was war das?" Und da ist derjenige, also hat er keinen Ärger gekriegt, sondern der Lehrer hat denn vernünftig erst mit ihm gesprochen, nicht gleich so angemeckert, sondern er hat erst gefragt, was denn ist und "das sollst du aber nicht wieder machen" und dann wollte er vor's Lehrerzimmer, weil wenn er manchmal vor's Lehrerzimmer muss, muss er immer so n paar Wutanfälle kriegen, das ist nämlich auch n bisschen manchmal blöd. Dann kriegt er richtig Ärger und dann zum Beispiel rennt er durch, rennt er durch die Tür und dann wissen wir "aha haltet euch jetzt die Ohren zu", sagen wir dann manchmal. Und dann, weil das, der ballert immer die Tür richtig zu, das ist ja so 'ne Glastür. Wir dach- wir denken manchmal das Glas platzt. Und er hat letztens mal so die Tür zugeknallt. Und immer wenn er denn wutausbrüchlich irgendwo hingeht, in den Gruppenraum oder so, dann wissen wir "aha, jetzt schnell Ohren zuhalten, sonst ballert's richtig laut".

Ja, der hat immer die Tür so richtig zugeballert und das tat voll in den Ohren weh.

I: Ja, das glaub ich. Das ist ja auch nicht schön. Habt ihr denn dann überhaupt noch Unterricht richtig machen können, wenn so was war?

P: Also, dann hat unser Lehrer gesagt, dass die Klassensprecher kurz hinterher gehen sollen, oder ein vertrautes Mädchen macht das immer. Das war dann so n Mädchen, der Junge hat diesem Mädchen immer vertraut und so. Und deswegen musste dieses Mädchen dann immer in– mit dem Klassensprecherin oder Klassensprecher dorthin. Und, die haben das dann immer so geklärt. Und haben gesagt "was ist los?" Und haben ihn dann erstmal beruhigt und vernünftig befragt und haben gesagt, haben die gesagt "sollen wir denn jetzt langsam wieder in die Klasse gehen? Ich werde auch unser'm Lehrer sagen oder Lehrerin, dass du jetzt keinen Ärger kriegen sollst". Weil manche Kinder haben denn ja auch immer Angst, dass sie Ärger kriegen.

I: Ja, ja das glaube ich. OK, super. Fällt dir noch was ein, was du gerne sagen möchtest, zu dem Thema?

P: Hm.

I: Nee?

P: Nee, aber, wenn so ein Junge, fiel mir gerade noch ein, der Junge, der immer diese Wutausbrüche gekriegt hat. Ist er manchmal auch zu Frau XY gegangen, das ist eine vertrauliche Lehrerin bei uns an der Grundschule XX und, die sagt dann immer "was ist passiert?" und dann redet die immer so, oder. Wir hatten jetzt 'ne neue Regel gekriegt in der vierten Klasse. Da stand dann immer ne Tafel, zum Beispiel, wenn du angefasst wurdest, vom Jungen oder so. Das ist mir auch schon mal- einmal passiert in der Klasse. Auch schon mal einen von Erwachsenen. Und, ganz vielen

Mädchen aus unserer Klasse ist das schon passiert. Und dann sind wir dahin gegangen, haben das gesagt, und die haben sich 'n Zettel genommen, das alles aufgeschrieben. Da haben die immer so privat mit den Lehrer geredet, sind die nach draußen gegangen und so. Und zum Beispiel, ist letzt- hat eine Junge mit Absicht den Feueralarm gelöst und da dachten wir alle "oh mein Gott, jetzt brennt alles in Flammen", und unser Lehrer dachte das auch. Und wir sind so schnell wie es ging aus'm Gebäude alle raus gerannt.

I: Ja, das glaube ich.

P: Und da ist Frau XY auch gekommen, weil unsere ein-zwei Mädchen wussten, wie der Junge ausgesehen hatte und deswegen mussten die ihn jetzt aufmalen.
Mh, weil die brauchten 'n paar Beweise.

I: Das ist ja wie im Fernsehen, fast. Oh, Wahnsinn. Und das hilft, wenn Frau XY dann kommt und mit einem redet?

P: Ja, und dann sagt der Junge zum Beispiel auch "ich mach es nicht wieder. Das war keine Absicht. Ich bin da ausversehen mit der- mit dem Ellbogen oder so dran gekommen".

I: Hm, oh je, Gott Feueralarm, das ist ja nicht gut.

P: Einmal hatten wir so 'ne Übung gemacht und da kam dann so Qualm aus einem Raum. Und ich dachte erst "oh hoffentlich sterbe- jetzt, glaub ich". Dachte ich erst so.

I: Oh da hätte ich aber auch Angst.

P: Und da dachte ich, müssen wir muss jetzt aus dem Fenster springen, weil das da jetzt alles in dem Raum, nachher kommt da so 'ne große Flamme, weil da irgendjemand Öl hin gespritzt hat oder so. Und dann sterbe

ich. Und ich dachte schon, oh und dann ist die Feuerwehr gekommen und das war nur 'ne Übung. 'Ne Rauch- ähm 'ne Rauchmaschine war das.

I:Ah ja, ok, oh Gott, das ist aber auch gruselig.

P: Und ich bin dann auf dem Boden gekrabbelt, weil ich dachte das wär echtes und da bin ich aufgestanden, hab ich gerochen, da dachte ich "so riecht kein Qualm". Und deswegen bin ich dann schnell dahin gelaufen. Weil, das macht man ja eigentlich, wenn man im Gebäude eingeschlossen ist, damit man- weil unten ist weniger als oben, oben ist viel mehr.

I: Ja, da hast du aber gut reagiert, da muss man in so 'ner Situation auch dran denken. Nicht schlecht. Ich beende mal hier die Aufnahme.

Transkription des Interviews mit Student 2

<u>Legende:</u>

Interviewer=„I“; Proband=„P“

Pausen=(...)

unverständliches Wort=((unv.Wort))

Wort- und Satzabbrüche= (-)

- Anonymisierungen=[…]

I: So. Dann fangen wir doch erstmal an mit: Gehst du denn gerne zur Schule?

P: Ja! Sehr gerne.

I: Schön! Und hast du auch Freunde bei dir an der Schule?

P: Ja.

I: Ja?

P: Ja, ganz viele.

I: Oh. Sehr schön. Gibt es manchmal Momente, in denen du dich nicht so gut fühlst, wenn du wütend bist –

P: Ja -

I: oder traurig oder Angst hast oder so?

P: Ja, manchmal, wenn dann der Junge kommt und dann will der, hat der immer angedroht, dass er mich schlagen will und so.

I: Und das war ein Mitschüler von dir?

P: Mhm. (zustimmend)

I: Ja. Und das war in der Schule, dass der das gemacht hat?

P: In der Schule hat der mich in die Ecke und dann hat der mich, mitten auf dem Pausenhof, vor allen anderen Kindern hat der mich geschlagen.

I: Oh Gott. Und was hast du dann gemacht?

P: Ja, ganz schön geweint und bin zu meiner Freundin gelaufen und dann zum Lehrer.

I: Ja. Gibt es noch andere Momente?

P: (überlegt) Eigentlich nicht.
Ja, wenn meine Freundin mich manchmal in der Pause, - hat sie gesagt, ich mache jetzt was mit dir, damit ich dir helfen kann und dann hat sie mich einfach stehen gelassen.
Ja, dann ist sie zu ihren Anderen gegangen und dann hat sie mich immer ausgelacht und so. Vielleicht –

I: Warum hat sie dich ausgelacht?

P: Ja, vielleicht weil ich dann anscheinend nicht so bin wie sie und –
Ja und ich fand sie nicht toll. Ja.

I: Ja. Und was passiert dann, wenn es dir nicht gut geht? Was machst du dann?

P: Dann bin ich eigentlich traurig und dann schlucke ich es eigentlich runter. Also ich versuche es manchmal, aber manchmal geht das auch nicht. Dann fange ich auch ein bisschen an zu weinen.

I: Ja, das glaube ich! Könnte denn jemand anderes merken, dass es dir nicht gut geht? Würde ich dir das anmerken?

P: Ja. Eigentlich schon.

I: Ja? Wie würde ich das denn sehen, wenn es dir nicht gut geht?

P: Also dann rede ich eigentlich gar nicht mehr, obwohl ich so eine Quasseltante bin und dann möchte ich auch nichts mehr essen und dann merken die das andere.

I: Und deine Mitschüler merken das dann auch?

P: Ja, weil ich die immer voll quassel eigentlich. Manchmal sagen die „Sag mal, hörst du jetzt mal auf zu reden, weil du mich so voll quasselst.“ und ich so „Ja, ist in Ordnung.“ und wenn es mir nicht so gut geht, dann rede ich eigentlich gar nicht, das merken die dann auch. Dann fragen die auch „Warum redest du denn nicht mit mir?“, ich so „Ja, weil ich traurig bin und so.“

I: Okay. Ja. Und deine Lehrer merken die das auch, wenn es dir nicht so gut geht.

P: Nein. Denen fällt das gar nicht auf.

I: Ok. Das heißt im Unterricht bist du dann trotzdem –

P: Ja. Mache ich trotzdem mit.

I: Ok. Was hilft dir denn, damit es dir dann besser geht, wenn du mal traurig oder wütend bist oder mal Angst hast?

P: Also eigentlich meine beste Freundin und meine Eltern und meinen kleinen Kuschelhund.

I: Der hier unten liegt?

P: Ja! Und mein (unverst. Wort)

I: Und was, wie können deine Freunde dir helfen, damit es dir besser geht?

P: Also die gehen dann mit mir auch dahin, dann gehen sie mit mir zum Lehrer und dann unterstützen die mich und so.

I: Ja. Und hast du sie gefragt, ob sie dir helfen können oder merken sie das so?

P: Nee, die merken das eigentlich so.

I: Oh, das ist ja super! Schön, wenn man solche Freunde hat. Und deine Lehrerin oder dein Lehrer, wie können die dir helfen, wenn es dir nicht so gut geht?

Können sie dir überhaupt helfen oder sind das dann nicht die Richtigen?

P: Eigentlich nicht die Richtigen.

I: Nee?

P: Nee.

I: Ok. Hätten sie dir denn mit dem Jungen irgendwie helfen können?

P: Sie hätten! Und sie haben ihn auch manchmal nicht im Unterricht mitmachen lassen und dann musste ich immer die suchen gehen. Ja. Und immer musste ich immer die suchen gehen und immer den Jungen, der mich so komisch angemacht hat und den musste ich ausgerechnet suchen gehen.

I: Und dann?

P: Nur weil er mit mir immer mitgegangen ist, weil der voll in mich verknallt war!

I: Und trotzdem hat der dich so geärgert?

P: Ja. Der war so eifersüchtig, weil ich ja auch manchmal was mit den anderen Jungs was gemacht habe in der Pause, zum Beispiel Ticker gespielt, aber der war so eifersüchtig, der hat die alle verkloppt!

Ja, obwohl ich den gar nicht mochte. Der war voll blöd, weil er mich ja auch immer angepackt hat und geschlagen hat.

I: Oh, man! Und hast du darüber mit deinen Lehrerinnen und Lehrern geredet?

P: Ja, hab ich.

I: Ja? Und konnten die dir irgendwie helfen?

P: Nee, die konnten mir nicht so richtig -, nicht so-, ich bin dann immer zu Frau Name1 gegangen, die hat mir dann geholfen.

I: Aber die konnte dir helfen?

P: Ja.

I: Und was ist dann mit dem Jungen passiert?

P: Also, dem ist gar nichts passiert. Nichts! Der hat immer weiter einfach Kinder verhauen und so.

I: Echt?

P: Ja!

I: Oh je. Das ist ja furchtbar! Wie merkst du denn bei deinen Freunden, wenn es ihnen nicht so gut geht?

P: Also meistens stellen die sich in die Ecke und heulen. Ja. Dann weiß ich immer schon, dass was ist. Oder sie reden auch nicht mehr.

I: Ah ja. Ja. Und bei dem Jungen, wie habt ihr da gemerkt, dass es vielleicht heute ein schwieriger Tag ist oder –

P: Gar nicht. Er hat einen auf „happy“ gemacht und hat einen dann verprügelt.

I: Tatsächlich?

P: Ja.

I: Also konnte man gar nicht ahnen?

P: Nee. Der kommt einfach von hinten an und hat dich dann ko gehauen.

I: Ach Gott. Und was hat der sonst noch so im Unterricht gemacht?

P: Also, der hat immer mit spitzen Bleistiften in die Haut gepikst von den anderen. Dann hat er den Mädchen immer fast die Haare abgeschnitten. Hat der meinen Zopf festgehalten und hat dann immer schon die Schere so richtig dran gehalten, manchmal und dann hat der einen immer den Schulranzen auch immer durch die Gegend geworfen. Einmal hat er sogar einen Tisch geschrottet.

I: Wie das denn?

P: Ja, er hat mit der Faust draufgehauen, dann hat er einmal noch doller und dann war der Tisch fast kaputt. Da war ein richtiger Riss drinne.

I: Oh Je! Und hattet ihr noch andere Schüler, die so ein bisschen –

P: Also Name 2, da sagen immer alle das ist ein Chaot, dabei war der voll lieb zu mir. Name 3, ja der hat auch manchmal ganz schön zugeschlagen. Und Name 3, Name 2 und Name 4! Der hat auch immer alle verkloppt!

Ja, der war auch in mich verliebt und dann hat der die Jungs auch immer alle hintereinander verkloppt. Obwohl ich ihn auch nicht mochte! Und die das nie gemerkt haben!

I: Ja. Da haben sie aber auch eine schwierige Art gewählt, dir zu zeigen, dass sie dich mögen! Und was haben die so im Unterricht gemacht?

P: Immer mit Stiften rumgepikert so den anderen oder immer irgendwie immer ganz doll immer rumgeschrien, dass sie auf sich aufmerksam machen oder dann sind sie mal-, dann haben sie mal den Türgriff und dann haben sie die Tür ganz dolle zugehauen, das hat man auch in den anderem Gebäude gehört.

I: Ach Gott. Oh man, da hattet ihr ja eine ganze Menge Schwieriger.

P: Und dann sind sie immer abgehauen, weil sie aus dem Unterricht raus mussten, weil sie uns immer verprügelt haben, dann mussten sie aus dem Unterricht raus, dann sind sie aus dem Gebäude rausgegangen, haben sich das Fahrrad geschnappt, sind nach Hause gegangen, haben zu ihre Mutter gesagt „Ich habe eine Freistunde!“.

I: Ach! Und was passiert dann? Wurden die dann gesucht?

P: Ja, ich musste sie dann wieder suchen mit Mitschüler 1.

I: Während der Schulzeit?

P: Ja. Wir wurden aus dem Unterricht rausgeholt und sollten die suchen.

I: Ach! Und dann wurdet ihr alleine losgeschickt?

P: Ja. Und dann mussten wir ihn suchen. Aber wir haben ihn nie gefunden! Weil der sich immer versteckt haben. Wenn wir ihn dann gefunden haben, dann hat er uns verkloppt. Dann sind wir wieder reingekommen und bis wir dann manchmal geweint haben.

I: Ja, das glaube ich. Und was ist dann passiert? Hat euch da jemand geholfen?

P: Ja, der Lehrer ist dann einmal mitgekommen und hat gesagt „Komm mit!“ und hat er ihn dann mitgezogen und dann war das nicht mehr.

I: Ok und das war es dann?

P: Dann war das geregelt, haben die gesagt.

I: Ok. Das ist ja schwierig. Was passiert denn in so in der Klasse, wenn jemand anfängt zu schreien oder so –

P: Also dann kommt noch der andere Chaot dazu, dann stellen die sich in die Mitte und dann verprügeln die sich beide.

I: Während des Unterrichts?

P: Ja.

I: Ok.

P: Dann kam ja der Herr XY noch, der sollte Name 5 ja noch betreuen, aber das hat er ja nicht gemacht! Er hat eher auf mich aufgepasst! Und hat mich dann -, und dann hat er auch meine Freundinnen am Hintern gepackt und dann hat der auch manchmal mich richtig ausgeschimpft, obwohl ich gar nichts gemacht habe! Ich habe nämlich mal meiner Lehrerin ein Problem erzählt mit Name 5 und dann hat, dann ist er zu mir gekommen und dann hat er mich ausgeschimpft und zwar so lange -, das war eigentlich-, und dann hat er mich eine halbe Stunde lang nur ausgeschimpft, da hab ich zwei Stunden lang geweint und bin nicht mehr zum Unterricht gekommen, weil ich dann zu einer anderen Lehrerin gegangen bin.

I: Ja. Warum hat der dich denn ausgeschimpft?

P: Ja weil ich ja anscheinend der Lehrerin nicht die Wahrheit gesagt habe und aber hab ich ja. Ich habe der Lehrerin die Wahrheit gesagt.

I: Und dann hast du den Ärger gekriegt?

P: Ja, weil ich die Wahrheit gesagt habe und er gesagt hat, dass ich lüge und dann hat er mich vor den ganzen Lehrern dann als Lügnerin dargestellt.

I: Tatsächlich? Das ist ja furchtbar.

P: Mhm, ich habe mich auch immer geschämt!

I: Ja. Dabei ist es doch wichtig sowas zu sagen und darüber zu reden. Oh Gott. Und konntet ihr das irgendwie lösen? Was ist dann passiert?

P: Also dann ging das drei Jahre so – Ja. Und zwei Wochen vor unserer Abschlussfeier, bevor ich von der Schule gegangen bin, wurde er gefeuert. Das hätten die auch gerne eher machen können.

I: Ja. Und der war drei Jahre lang bei euch in der Klasse?

P: Ja, der hat nur Unfug gemacht.

I: Ja. Das klingt wirklich so.

P: Und dann sollte er mir auch manchmal helfen, weil ich in Mathe manchmal nicht so klar gekommen bin und da hat er mir das immer erklärt, wie ich das machen sollte und hat gesagt, dass ist alles so richtig und dann hab dachte ich, dass das so richtig wäre, hab das genauso zu Hause geübt und dann war das alles falsch. Der hat mir alles falsch erklärt!

I: Das ist ja komisch! Hattet ihr denn dann überhaupt richtigen Unterricht, wenn das so-, wenn ihr so viele schwierige Schüler hattet?

P: Eigentlich nicht, wir waren die schlechteste Klasse, wir haben alle nur fünfen geschrieben, weil wir immer so belastet worden sind. Ich bin immer noch gut mit einer vier oder mit drei weggekommen.

I: Echt. Oh. Ja, immerhin. Und was hat euer Lehrer dann so gemacht, wenn es im Unterricht so schwierig wurde?

P: Ja der hat gesagt „Setz dich hin, gibt einen Klassenbucheintrag“ ins Klassenbuch und dann „Entschuldige dich“ und meistens haben wir die dann gar nicht mehr angenommen, weil er es ja sowieso immer wieder gemacht hat! Und er dann so „Die nimmt aber meine Entschuldigung nicht an!“ „Nimm sie sofort an!“ „Warum?“ „Musst du!“ „Ja, gut!“. Dann haben wir die angenommen, dann war anscheinend ja wieder alles in Ordnung und wir sind mit blauen Augen nach Hause gekommen!

I: Und es gab aber keine - ?

P: Nee. Der hat sogar einem Mitschüler einen Arm gebrochen. Ja, der hat den umgedreht, den ganzen Arm und dann hat er gesagt „Stopp!“ und dann hat er weitergedreht und dann war der gebrochen.

I: Oh Gott! Im Unterricht oder auf dem Schulhof?

P: Nee auf dem Pausenhof hat der dann den Arm genommen, den umgedreht und dann war der gebrochen.

I: Oh Gott! Und ist dann da irgendwas passiert? Wurde der irgendwie bestraft?

P: Ja er durfte am nächsten Tag nicht zur Schule kommen und einen Klassenbucheintrag und mehr nicht und eine Entschuldigung!

I: Und wie geht es euch dann, dir und deinen Freunden, wenn ihr sowas mitbekommt?

P: Eigentlich haben wir dann selber Schiss, weil uns das ja auch passiert ist, dass wir dann so behandelt worden sind.

Wir wollten gar nicht mehr zur Schule kommen, weil wir so Schiss hatten, dass er uns verprügelt. Einmal da konnte

ich sogar gar nicht mehr richtig schlucken und hab keine Luft mehr bekommen, weil er mir mit der Faust in den Hals geschlagen hat. Und er so-, und ich so „Warum hast du das gemacht?“, habe ich ihn am nächsten Tag gefragt und er so „Weil es mir Spaß gemacht hat.“ „Das habe ich ausgerechnet übrigens noch mit Absicht gemacht. Eigentlich wollte ich nur so machen, ganz leicht, aber dann habe ich volle Kanne zugehauen.“, hat er gesagt, „weil ich ja gerade die Chance hatte!“

I: Oh Gott. Oh man. Das ist ja wirklich ganz schön viel, was bei euch los war. Wahnsinn. Konntest du deinen Freunden irgendwie ein bisschen helfen, wenn es denen dann auch nicht so gut ging, wenn die traurig waren oder Angst hatten?

P: Nee, wir wurden ja immer alle zusammen verprügelt!

I: Oh man. Das ist echt ja echt Wahnsinn. Kein Wunder, dass ihr Angst hattet zur Schule zu gehen! Aber jetzt ist es besser?

P: Jetzt ist es besser, auf der neuen Schule.

I: Ja? Okay. Gut. Und ihr seid auch immer zur Schule gegangen oder seid ihr auch manchmal –

P: Doch wir sind dann noch zur Schule gegangen. Aber nicht mehr so gerne, weil wir wollten-, wir haben immer sehr viel Spaß an der Schule gehabt, weil wir auch gerne die Lehrer mochten, aber dann gar nicht mehr. Weil wir nur noch geärgert wurden von dem und so und dann hat der uns immer Liebesbriefe-, die hab ich auch noch oben. Voll blöd! Der hat immer geschrieben „Ich liebe dich! Ich liebe dich! Ich will mit dir Sex.“ und so was. Hat er dann immer geschrieben. „Komm mit mir aufs Jungsklo!“.

I: Oh Gott! Ok. Und hast du die irgendeinem Lehrer gezeigt?

P: Ja, die hab ich dann mal mitgebracht, da bei Frau Name 1, ihr hab ich die dann mal gezeigt und sie so „Die musst du in den Müll schmeißen!“. Aber die konnte ich nicht-, die wollte ich nicht in den Müll schmeißen, weil wenn mal noch was ist, dass ich die dann nochmal zeigen kann.

I: Ok. Und hast du mit ihm mal gesprochen oder konnte man mit ihm gar nicht reden?

P: Doch mit ihm habe ich gesprochen! Er hat ja gesagt, er macht ja nichts. Und in der nächsten Pause hat er mich wieder verkloppt.

I: Ist das denn auch während des Unterrichts passiert oder –

P: Ja! Auch manchmal während des Unterrichts. Da hat er mich geärgert. Da stand er immer hinter mir und hat mir manchmal so in den Rücken geboxt und so.

I: Oh man. Da habt ihr echt ganz schön was mitgemacht.

P: Mhm.

I: Ja. Naja. Und jetzt in der Klasse ist aber –

P: Doch hab ich wieder so einen. Ja, der hat jetzt sogar mein, von meinem-, von meinem Freund, so der hat dann da einen auf die Mütze bekommen und dann hat er ihm fast das Genick gebrochen, weil er sich hier drauf gesetzt hat und hat er ihn auf den Bogen geprügelt. Und meine Freundin ist dazwischen gegangen und die hat auch eins auf die Mütze

bekommen. Dann ist der Freund von dem Freund wieder dazwischen, der wurde dann auch auf den Boden und ist mit einem blauen Auge dann noch davon gekommen. Der andere hatte fast einen Genickbruch.

I: Und was macht der noch so, der da bei euch in der Klasse ist?

P: Also der stört immer und dann schnarcht der immer so und macht den Kopf so " Schnarch" im Unterricht - Und dann sagt er „Der Unterricht ist langweilig.“ zu der Lehrerin und dass sie selber blöd sind. Ja.

I: Und was machen die Lehrer dann?

P: Ja, die schlucken das runter und mehr nicht.

I: Ja. Aber für euch ist dann sicherlich auch schwierig oder? Oh je. Das ist ja ganz schön viel, was da bei euch los ist. Also, dass ihr da trotzdem noch aufs Gymnasium gekommen seid, Hut ab! Wahnsinn. Gibt es sonst noch was, was du da-, was da passiert ist?

P: Mit Herrn XY, der hat meine Freundin immer an den Hintern gepackt!

I: Was?

P: Ja, der hat die voll begrabschelt und so.

I: Und was hat sie dann gemacht?

P: Ja, sie ist zu einem Lehrer gegangen, nur die haben uns manchmal nicht geglaubt!

I: Wirklich?

P: Ja, weil das ja der Gesprächspartner von denen war und der denen ja alles anvertraut hat und dann hat der was-, der Lehrer hat dann ja auch manchmal so erzählt, wie wir uns entwickeln und dann hat er immer um neun Uhr bei uns zu Hause angerufen, was ich alles so in der Schule gemacht habe, ob ich mir was in der Mensa geholt habe oder so.

I: Das hat der dann deinen Eltern erzählt?

P: Ja. Und Mama so „Achso, dann brauchst du ja neue 15ct!“, ich so „Ja!“, „Ja, dann gebe ich dir die jetzt.“. Ja dann „Warum ruft denn dieser Trottel wieder bei uns an? Der hat jeden Abend bei uns angerufen! Und immer aufs band gesprochen und so. Und irgendwann war das Band so lange, da hat der sogar zwei Stunden lang mit meiner Mama telefoniert. Wie toll ich bin und sowas. Und dass ich in der Mensa war und mir einen Kakao geholt habe und –

I: Und wie hast du dich dann dabei gefühlt, wenn du das gehört hast?

P: Nicht so gut, weil manchmal hab ich mich ja auch mit meinen Freunden getroffen in der Pause und dann hat der das auch erzählt.

I: Das geht ja gar nicht.

P: Nee.

I: Oh man! Aber könntest du denn mit deinen Eltern darüber reden?

P: Ja mit denen hab ich geredet.

I: Und mit deinen Freunden?

P: Mit meinen Freunden habe ich auch geredet, die hatte alle das gleiche Problem. Weil der hat immer-, bei mir hat der dann den Tag angerufen, zwei Stunden und den nächsten Tag dann nicht, da hat er dann bei einer anderen Freundin angerufen und hat den Eltern das erzählt. Dann wieder nicht, da hat er bei einer anderen Freundin und dann wieder bei mir und dann immer weiter!

I: Oh je. Und dann ging es euch-, dann habt ihr alle gedacht, dass ist komisch oder?

P: Ja.

I: Das glaube ich. Das ist dann der, der gefeuert wurde?

P: Ja. Der hat auch geraucht in der Schule!

I: Vor euren Augen?

P: Das ist den Lehrern nur nie aufgefallen, der ist immer hinter das Gebäude und dann hat er da so geraucht, aber er war noch auf dem Schulgelände, man darf ja nicht auf dem Schulgelände rauchen!

I: Nee, auf gar keinen Fall!

P: Hat der die dann hingeschmissen, da hinten.

I: Ach. Und drei Jahre war der bei euch in der Klasse?

P: Ja, drei Jahre! Hab ich gut ausgehalten.

I: Ja, aber wirklich!

P: Bis zum Schluss, bis ich die Schnauze voll hatte und dann hab ich den Lehrern mal alles erzählt und dann hat er immer so-, dann hat er einmal so durchs Fenster geguckt, ob ich da sitze.

I: Oh Gott. Und dann hat der Lehrer was gemacht, als du ihm das endlich erzählt hattest oder?

P: Nee das war-, ich hatte das meinem Lehrer nicht erzählt, weil -, ich hab das meiner, also Frau XY erzählt. Also die ist ja die Psychologin bei uns. Und meinem Lehrer den habe ich das dann auch erzählt und er so „Da müssen wir ja was machen“, ich so „Ja, müssen wir was machen“, „Was denn?“, ich so „Weiß ich doch nicht. Das müssen sie doch wissen“. Und dann ist aber auch nichts passiert, außer bei Frau XY, die hat dann mal die ganze (unverst. Wort) und dann hat sie den zwei Wochen, bevor ich von der Schule gegangen bin, hat sie ihn gefeuert. Und jetzt ist er wieder bei dem Jungen. Auf der anderen Schule.

I: Echt?

P: Ja. Und ausgerechnet die Mutter von dem Jungen hat den angeschafft. Die hat den angeschafft, den angerufen, dass er jetzt immer kommen soll. Und dann hat der mir auch immer seine Privatangelegenheiten erzählt.

I: Der Lehrer?

P: Nee, der Aufseher, da. Ja! Der hat immer erzählt so, wie es bei den anderen da zu Hause aussieht und wo er wohnt und –

I: Das sollte man ja eigentlich nicht weiter erzählen. Das ist ja wirklich ein bisschen komisch.

Student 2

P: Und ich dachte nur so „Sie haben doch Schweigepflicht“. Ja. Und er hat mir alles erzählt. Ob da Unterhosen lagen oder nicht.

I: Das ist ja echt komisch. Wahnsinn, was ihr mitgemacht habt!

P: Dann lief dieser Protz da immer so und hat mich immer so angeguckt „Ja, hallo.“ Und dann hat der immer voll nach Rauch gestunken!

I: Ja, glaube ich, wenn der da mitten auf dem Schulgelände geraucht hat.

P: Mhm und einmal da hat er mal zu meiner Freundin, da hat sie nur mit mir geredet und da hat er gesagt „Weißt du eigentlich, was das bedeutet, wenn man mit Steinen wirft?“ und sie so „Äh, was denn?“ und er so „Du musst mal googlen, weil du ja nichts weißt.“

I: Was?

P: „Du musst ja mal das googlen, was ich dir gerade gesagt habe, weil du ja nie weißt, was ich sage.“ und wir so „Äh...!“, sie hatte ein riesen Fragezeichen im Gesicht!

I: Ja, das kann man verstehen!

P: Ich auch, ja. Weil ich auch nicht wusste, was und er meinte uns beide, aber wir haben ja gar nichts gemacht! Wir waren ja nur am frühstücken.

…

Transkription des Interviews mit Student 3

<u>Legende:</u>

Interviewer=„I“; Proband=„P“

Pausen=(...)

unverständliches Wort=((unv.Wort))

Wort- und Satzabbrüche= (-)

- Anonymisierungen=[…]

I: Gehst du denn gerne zur Schule?

P: Hmm, ja. Also in der Grundschulzeit oder jetzt?

I: Das ist egal, kannst du ganz allgemein sagen.

P: Ja.

I: Und hast du auch Freunde bei dir an der Schule?

P: Hmm, einen nur, weil die anderen kenne ich ja noch nicht so gut in meiner Klasse. Und einen, den mag ich auch und der ist auch aus der Grundschulzeit.

I: Und der ist mit dir rüber gekommen?

P: Ja

I: Und in der Grundschule, da hattest du mehr Freunde?

P: Ja, da hatte ich mehr Freunde.

I: Kennst du das denn, dass es dir mal nicht so gut geht?

P: Ja.

I: Kannst du dich da an irgendwas erinnern, wann das vielleicht mal war?

P: In der Grundschulzeit, ja. So in der 3./4. Klasse.

I: Und was war da? Weißt du das noch?

P: Also, so ein Junge, der hat mich die ganze Zeit geärgert und so und der hat dann die ganze Zeit gesagt, dass ich keine Markenklamotten anhabe und hat die ganze

Zeit auf meine Schuhe geguckt und hat mich dann auch über WhatsApp beleidigt. Aber dann auf seiner Sprache. Der hatte so eine andere Sprache, ich weiß gar nicht wie die hieß. Und dann hat er die ganze Zeit so Schimpfwörter an mich geschrieben, aber dann aus seiner Sprache. Da mussten wir erstmal googlen was das bedeutet.

I: Und was machst du dann wenn es dir nicht so gut geht?

P: Ich sag das Mama und habe das dann zuletzt auch Lehrerin XY gesagt.

I: Das ist ja gut. Und was passiert dann? Konnten die dir helfen, deine Mama oder Lehrerin XY?

P: Ja, der Junge der hatte so fast ne Strafe gekriegt, wenn er das noch mal macht, dann – er war schon einmal in der vorherigen Klasse und da hat er das auch gemacht bei jedem und dann ist er in unsere Klasse gekommen. Und da hat er das auch bei jedem gemacht. Lehrerin XY und die, die haben ihn dann bestraft und haben gesagt, wenn er das noch mal macht, kommst du in eine andere Klasse. Zuletzt dann hat er sich immer benommen.

I: Und hast du dich dann auch mit ihm vertragen?

P: Also, ich habe gar nicht mehr mit ihm geredet. Ich fand ihn einfach nicht mehr so cool wie er vorher war.

I: Kennst du das denn von dir selber auch vielleicht, dass du mal, wenn es dir nicht gut geht, andere ärgerst?

P: Ja also wenn die jetzt zum Beispiel mich ärgern, dann sag ich das schon mal oder schlag zurück oder so, weil ich kann ja nicht immer weglaufen. Das ist ja blöd.

I: Könnte ich denn, wenn es dir mal nicht gut geht, also würde ich das merken? Zeigst du das?

P: Na ich wär dann traurig und wütend. Dass er mich die ganze Zeit schlägt, dass er mich dann beleidigt.

I: Und haben deine Mitschüler das auch gemerkt?

P: Also der Freund von ihm, der hat das auch gemerkt, dass der ihn beleidigt und dann habe ich ihm das erzählt, dass er... und da sagte er, genau das gleiche Problem habe ich auch. Und dann habe ich das meinen Mitschülern erzählt, aber nur die ich gut kenne und die haben dann gesagt, wenn er das noch mal macht, helfen sie mir dabei.

I: Und konnten sie dir helfen?

P: Ja, die haben dann gesagt, Name1 hör damit auf. Hör auf ihn zu beleidigen und so.

I: Und hat dir das geholfen?

P: Ja.

I: Ging es dir dann besser?

P: Ja.

I: Was hilft dir denn noch, wenn es dir mal nicht gut geht, damit es dir dann wieder besser geht?

P: Vielleicht mit meinen Freunden spielen oder mit meinen Nachbarn spielen.

I: Was spielt ihr dann so?

P: Wir fahren meistens Fahrrad oder wir spielen, aber das ist nur manchmal, PS4, dann spielen wir so ein Spiel zusammen.

I: Und dann geht's dir besser?

P: Mhm. Das lenkt mich auch ab.

I: Und gibt es irgendwas in der Schule, was du dann machen kannst, wenn es dir nicht so gut geht?

P: Also eigentlich kann ich nur zu meinem Freund gehen oder in den Freizeitbereich.

I: Sowas habt ihr auch?

P: Ja, wir haben so einen Bereich, da kann man dann Tischkicker oder Billard spielen, da ist auch ein Sofa. Da sind auch Sozialarbeiter, die können dir auch helfen.

I: Und wie können deine Freunde dir in der Schule helfen, wenn du sagst, du gehst dann zu deinen Freunden? Redest du dann mit denen oder spielt ihr was zusammen?

P: Wir spielen was zusammen, meistens Billard und reden auch.

I: Und können deine Lehrer dir auch helfen wenn es dir nicht so gut geht?

P: Also jetzt kenne ich ja nicht so gut alle Lehrer. Also im Moment noch nicht.

I: Und wie war das an der Grundschule? Konnten die dir helfen?

P: Ja, also außer Lehrerin XY, die anderen konnten mir nicht helfen. Lehrerin XY konnte mir immer helfen. Die anderen Lehrer, die mochte ich alle nicht.

I: Und was hat Lehrerin XY dann gemacht, um dir zu helfen?

P: Sie hat mich so ein bisschen beschützt und hat das denen auch gesagt, wenn sie mich noch mal ärgern, dann soll ich zu ihr kommen.

I: Und das tat gut?

P: Also ich war dann, ich hatte dann ein gutes Gefühl, dass sie mich dann auch so ein bisschen beschützt.

I: Kennst du das vielleicht auch von deinen Freunden, dass es denen mal nicht so gut geht?

P: Also ich hatte, wie ich schon erzählt habe, dieser eine, der auch das Problem hatte, bei dem habe ich auch gemerkt, manchmal wo er dann aufgehört hat, da hat er dann ja mit dem nächsten angefangen, den zu mobben und dann habe ich auch gemerkt, wie beleidigt die dann in die Schule gekommen sind.

I: Wie merkt man das denn? Wie hast du dann gemerkt, dass es denen nicht gut geht?

P: Die waren einfach traurig und haben dann m Unterricht fast gar nichts mehr gesagt.

I: Und wie merkst du das z.B. bei den anderen Mitschülern, wenn die irgendwas haben, zum Beispiel jetzt bei dem, der dich geärgert hat? Merkt man da irgendwie heute ist ein guter Tag, heute nicht?

P: Also der ist immer zu spät gekommen. Der war fast immer frech. Der war nie traurig, der war immer wütend.

I: Und wie hat der das so gezeigt?

P: Der hat immer jedem gedroht einem ins Gesicht zu hauen oder wenn er nicht darunter springt, dann gibt's irgendwie... muss der irgendwie was machen oder so...

I: Hat man das auch im Unterricht gemerkt? Hat der das da auch gezeigt?

P: Ja, der hat die Lehrer geärgert.

I: Und wie haben die dann reagiert?

P: Die Lehrer? Die eine, die hat nie was gemacht, die hat sich immer weiter ärgern lassen. Und irgendwann haben die sich auch mit mir angefreundet und da musste ich mitmachen. Das war komisch.

I: Da musstest du mitmachen bei...

P: Ja, da wo dann die Lehrer geärgert wurden.

I: Und was habt ihr da so gemacht?

P: Also, wir saßen immer unterm Tisch und immer beim Unterricht haben wir uns hinter den Gardinen versteckt. Oder immer wenn sie sich umgedreht hat, haben wir gequatscht oder so.

I: Und wie ging's dir dabei, wenn du das gemacht hast?

P: Also die hat nie was dazu gesagt und darum haben wir auch immer weitergemacht.

I: Wurdet ihr irgendwann dafür bestraft?

P: Nein, sie hat nur gesagt, wir sollen damit aufhören und dann haben wir das auch gemacht.

I: Hat das denn Spaß gemacht oder wolltest du das eigentlich gar nicht machen?

P: Ich wollte das nicht, aber die sagten ja, ich soll da mit machen. Und mein Freund wurde dann auch dazu gezwungen. Und der hat das dann auch gemacht. Das war ein bisschen blöd.

I: Also eigentlich wolltet ihr das gar nicht machen?

P: Nee, aber wir mussten dann ja mitmachen.

I: Und hat man das in der Klasse gemerkt, wenn einer sich so blöd benimmt?

P: Ja, also ich saß ja fast neben, gegenüber von dem. Der hat dann auch im Unterricht gegessen. Der hat meistens auch zu den Mitschülern, hat denen die Zunge gezeigt oder so.

I: Während des Unterrichts?

P: Ja, wenn die sich umgedreht hat und hat was an die Tafel oder das Smartboard geschrieben, dann hat der Quatsch gemacht und dann wurden wir da mit reingezogen. Und zuletzt hat das fast die ganze Klasse gemacht, weil er jeden dazu gezwungen hat.

Student 3

I: Und konntet ihr dann überhaupt noch richtig Unterricht machen und was lernen?

P: Also das ist ja nur in Sachkunde gewesen und da konnten wir eigentlich gar nicht mehr lernen. Er hat uns nur die ganze Zeit angestupst und hat gesagt, mach dies jetzt eben und ich mach mit...

I: Und dann haben da alle mitgemacht?

P: Ja, Zuletzt ja.

I: Wie habt ihr dann damit aufgehört?

P: Die Lehrerin hat gesagt, wenn ihr da nicht mit aufhört dann gibt's Ärger, dann haben wir gesagt, dann hören wir damit auf. Und die ganze Klasse hat auch gesagt, dass wir damit aufhören sollen und dann haben wir das auch gemacht.

I: Und dann hat das auch geklappt?

P: Ja.

I: Waren die anderen aus der Klasse eher böse auf euch oder fanden die das witzig?

P: Ja also die meisten fanden das witzig, die einen waren auch böse. Also ich habe da nur ganz kurz mitgemacht. Dann habe ich auch gesagt, jetzt habe ich keine Lust mehr dazu und dann habe ich mich mit denen, die lieb sind, angefreundet und mit denen, die wütend auf die waren. Und dann habe ich immer mehr Freunden gesagt, hört damit auf und dann haben die das zuletzt auch gemacht.

Student 3

I: Wenn du merkst, dass es Freunden von dir nicht so gut geht, dass sie traurig oder wütend sind, versuchst du dann denen zu helfen?

P: Ja den meisten wohl. Ich musste das ja sozusagen machen, da ich Klassensprecher war. Dann habe ich mit denen Spiele gespielt und dann waren die schon wieder munter.

I: Das heißt, wenn es jemandem nicht so gut geht, spielst du mit dem. Machst du noch was anderes?

P: Ich helfe denen dabei, dass sie das irgendwie verkraften können.

…

Transkription des Interviews mit Student 4

Legende:

Interviewer=„I“; Proband=„P“

Pausen=(...)

unverständliches Wort=((unv.Wort))

Wort- und Satzabbrüche= (-)

- Anonymisierungen=[…]

I: Dann fangen wir erst mal an, in welche Klasse gehst du jetzt?

P: Erste.

I: Gehst du denn gerne zur Schule?

P: Ja.

I: Ja? Schön. Seit wann bist du jetzt in der Schule, seit August dann?

P: Meinst du auch, seit ich in der Vorschule bin?

I: Ach so, du warst auch schon in der Vorschule? O.k.

P: Ja.

I: Das war auch hier an der Schule? O.k.

P: Also, zwei Jahre. Also, ein Jahr war ich hier und ich weiß gar nicht mehr, wann die Einschulung war.

I: Ah ja, gut, aber dann kennst du dich hier ja auch schon ein bisschen aus. Und hast du hier auch Freunde an der Schule?

P: Ja, eigentlich nur zwei, mh drei. Ich habe nur drei, Schüler 1, Schüler 2 und Schüler 3.

I: Das ist doch super. Das ist ja schön. Ich habe ja grade schon gesagt, dass es mich interessiert, was du oder andere machen, wenn es ihnen nicht so gut geht. Gibt es denn manchmal Momente, in denen du dich nicht so gut fühlst, wo du vielleicht traurig bist oder wütend?

P: Ne.

I: Ne? Nie traurig? Ne?

P: Nur wenn, ich bin heute ein bisschen traurig, wenn beim Pausenhof gibt es Fahrzeuge, also Fahrzeugpausen. Und da gibt es halt so Liegefahrzeuge und so Fahrzeuge, wo man lange drauf fahren kann. Und da bin ich neulich, gestern erst, da waren zwei neue, die aufpassen und da habe ich die ganze Zeit das eine Fahrzeug gefahren, obwohl die anderen immer sagen, du darfst nicht immer das fahren. Und ich wollte immer halt so ein tolles, großes. Und bei den anderen kriege ich das auch, weil die immer sagen, ich darf nicht so lange immer auf einem Fahrzeug bleiben.

I: Ja, und da warst du ein bisschen traurig?

P: Weil dann die aktive Pause zu Ende war. Es ist zwar zweimal Fahrzeugwechsel, aber das habe ich nicht gehört. Und gar keiner hat auch gewechselt. Fast.

I: Ja, das ist natürlich schade. Und was machst du dann, wenn du so traurig bist?

P: Naja, das weiß ich nicht.

I: Redest du dann mit jemandem? Oder wirst du dann vielleicht auch wütend?

P: Also, wenn es richtig doll ist, dann bin ich wütend, aber (-)

I: Und was machst du, wenn du wütend wirst? Würde ich das sehen?

P: Dann sage ich das die Lehrerin, also Lehrerin 1 oder Lehrerin 2.

I: Und streitest du dich dann auch manchmal mit deinen Freunden und Mitschülern? Oder das nicht so?

P: Ja, weil Schüler 1 möchte immer so bossig sein.

I: Und dann streitet ihr euch manchmal?

P: Ja

I: Na gut, passiert ja manchmal. Würde ich denn merken, wenn es dir nicht gut geht? Würde ich das sehen, oder versteckst du das eher?

P: Weiß ich nicht.

I: Nicht so? Merkt denn Lehrerin 1, wenn es dir nicht so gut geht?

P: Weiß ich nicht.

I: Und deine Freunde?

P: Ja.

I: Ja? Und woran merken die das?

P: Naja, manchmal sage ich es auch. Aber sie wissen halt, wann es mir gut geht halt und wenn es mir schlecht geht.

I: Redest du mit denen dann darüber?

P: Ja, mit Schüler 1 mehr, weil, der ist in meiner Klasse.

I: Ach so, naja, dann ist das natürlich leichter. Und was macht Schüler 1 dann, wenn es dir nicht so gut geht? Wie kann der dir helfen?

P: Dann sagt der halt, dann sagt der das der, sagt der das der Lehrerin.

I: Ah, o.k. Und kann die dir dann helfen? Ist das gut, wenn die das weiß?

P: Ja.

I: Ja? Was macht denn die Lehrerin dann, weißt du das?

P: Ne, doch aber, sie redet mit denen.

I: Ah ja, o.k. Und das ist dann gut? Dann geht es dir besser? Gibt es denn etwas, was du gerne möchtest, wenn du traurig bist oder wütend? Wie man dir da helfen soll?

P: Ne, außer, dass er nicht mehr so, außer, dass er nicht mehr so doof sein soll wie vorher.

I: Ja, o.k. Was ist denn, wenn es jetzt zum Beispiel Schüler 1 oder deinen anderen Freunden nicht so gut geht? Siehst du das?

P: Ja, das merke ich.

I: Und woran merkst du das?

P: Dass er dann ja auch ein bisschen traurig ist halt und so was.

I: Sieht man das an seinem Gesicht, oder wie siehst du das, dass er traurig ist?

P: Das sehe ich an seinem Gesicht auch ein bisschen.

I: Und was machst du dann, wenn du merkst, dass es Schüler 1 nicht gut geht?

P: Versuche, ihn aufzumuntern, also zu muntern.

I: Wie machst du das denn?

P: Zum Beispiel sagen, dass er jetzt mal bestimmen darf, welches Spiel spielen wir.

I: Ja, da ist doch gut. Und bei deinen anderen Freunden, merkst du das da auch, wenn die traurig sind?

P: Nein, also bei Schüler 3 merke ich es nicht, nur bei Schüler 2 und Schüler 1.

I: Und ist das dann gut für die, wenn du mit denen redest und mit denen spielst? Hast du das Gefühlt, dann geht es ihnen wieder besser?

P: Ja, habe ich.

I: O.k. Und eure Lehrerin, Lehrerin 1, was macht die dann? Versucht die auch zu helfen?

P: Ja, versucht sie auch.

I: Was macht die denn dann?

P: Fragt, was los ist, oder so.

I: O.k. Ihr habt ja auch in der Klasse einen Schüler, der manchmal ein bisschen schwierig ist, nicht?

P: Schüler 4.

I: Was passiert denn da? Was macht der denn so?

P: Naja, wenn er (-). Er möchte meistens immer den Kindern helfen, und dann haut er den anderen Kindern und so.

I: Weißt du, warum er dann haut?

P: Nein, weiß ich nicht. Und dann kriegt ja er den Ärger, obwohl der nur helfen möchte.

I: Was meinst du denn, warum klappt das denn nicht, dass der hilft?

P: Weiß ich nicht.

I: Was macht der denn falsch?

P: In Land 1, das ist ja seine Heimat, da gab es halt gar keine (-). Da prügeln sich halt die Menschen. Deswegen muss Schüler 4 das lernen, dass man hier nicht mehr prügelt.

I: Und könnt ihr ihm dabei helfen, dass er das lernt?

P: Ja

I: Wie macht ihr das denn? Spielt ihr auch mit dem, oder (-)?

P: Ja, wir machen manchmal so Verabredungspausen.

I: Ah ja, das ist doch schön. Was macht er denn noch so im Unterricht?

P: Also, im Unterricht schreit er manchmal und ist laut. Und mehr weiß ich eigentlich nicht.

I: Hast du eine Idee, warum er das macht mit dem Schreien?

P: Weiß ich nicht, das weiß ich auch nicht.

I: Meinst du denn, dass es ihm vielleicht auch manchmal nicht so gut geht? Oder ist das vielleicht was anderes?

P: Weiß ich auch nicht.

I: Und wie ist das für euch, für die anderen Schüler in der Klasse, wenn schreit oder haut? Wie fühlst du dich dann?

P: Naja, mich stört das halt immer, wenn Schüler 4 schreit, weil, wir arbeiten ja.

I: Und dann könnt ihr nicht mehr so richtig weiterarbeiten?

P: Konzentrieren können wir uns dann nicht so doll.

I: Ja, das glaube ich. Passiert das denn oft?

P: Nein.

I: O.k. Also jetzt nicht jeden Tag.

P: Naja, also mittel.

I: O.k., jeden Tag oder nicht jeden Tag?

P: Nein, nicht jeden Tag. Aber muss halt nicht so (-), mittelmäßig einfach.

I: Und dann ist es schwierig, dass ihr was lernt, wenn es so laut ist. Ja. Und was machen die anderen Schüler, machen die dann mit zum Beispiel, wenn er laut ist?

P: Nein

I: O.k., das heißt, das ist dann nur er und alle anderen sind eher, wollen eher weiterarbeiten.

P: Ja.

I: Und in der Pause? Merkt man das da auch, dass er manchmal ein bisschen schwierig ist, oder ist das nur im Unterricht?

P: Nein, nur im Unterricht.

I: Das heißt, in der Pause merkt ihr das gar nicht so?

P: Nö.

I: O.k. Wirst du dann auch manchmal wütend, wenn er so was macht im Unterricht?

P: Nein.

…

Transkription des Interviews mit Student 5

<u>Legende:</u>
Interviewer=„I“; Proband=„P“
Pausen=(...)
unverständliches Wort=((unv.Wort))
Wort- und Satzabbrüche= (-)
- Anonymisierungen=[…]

I: In welche Klasse gehst du denn?

P: In die 1d. Wir sind die Giraffenklasse. Ich war die erste, die einmal die Giraffe mitnehmen durfte übers Wochenende.

I: Ihr habt sogar eine Giraffe? Super, so eine Stoffgiraffe?

P: Ja, das ist unser Klassentier.

I: Oh, das ist ja toll. Das ist ja richtig super. So was hatten wir früher nicht. Und gehst du denn gerne zur Schule?

P: Ja.

I: Ja? Schön. Und hast du bei dir auch Freunde in der Klasse?

P: Ja. Name 1, die kenne ich vom Ballett, weil, da haben wir, also wir sind da, ja wir haben da, da hatte ich einmal nicht, da sollten wir ein Partnerspiel machen und da hatte ich nicht, also niemanden der, weil der noch frei war. Da ist Name 1 gekommen und dann waren wir einfach ruckzuck dicke Freunde geworden. Und dann hat sie mich zu ihrem sechsten oder fünften Geburtstag eingeladen und dann war da halt noch eine und die heißt Name 2. Mit der bin ich jetzt auch dick befreundet. Wir hatten Partnerlook mit den Strickjacken.

I: Aus Versehen, zufällig?

P: Ne, ja zufällig, aber nicht aus Versehen.

I: Ach, das ist ja super. Und das sind jetzt deine Freunde?

P: Ja.

I: Und die sind auch bei dir in der Klasse.

P: Ja, und ich habe eigentlich noch mehr, noch vielmehr.

I: Echt, oh das ist ja toll.

P: Also, nicht nur in der Klasse.

I: Echt, auch noch in den anderen Klassen?

P: Ja.

I: Das ist ja toll. Das heißt, du fühlst dich hier auch ganz wohl?

P: Ja.

I: Ja, das ist ja schön, super. Gibt es denn trotzdem auch manchmal Momente, in denen du dich vielleicht nicht so gut fühlst.

P: Ja, wenn jemand mich ärgert oder so. Weil, wenn man mich haut, tut es halt weh. Und das mag man auch nicht.

I: Ne, ne, das glaube ich. Passiert das denn manchmal, dass dich jemand haut oder dass dich jemand ärgert?

P: Ja, aber ganz selten.

I: O.k., zum Glück. Und wie fühlst du dich dann? Was ist dann?

P: Also, ich bin traurig, weil (-). Eigentlich, einmal hat das mal eine Freundin gemacht. Da war ich richtig traurig. Weil, ich habe irgendwie, naja, mein Bruder auch (-). Ich habe irgendwie bei einem Spiel, was es eigentlich gar nicht gibt, habe ich irgendwas falsch gemacht, und dann hat er gesagt „Geh weg“. Und hat mich gehauen oder so was.

I: Dein Bruder?

P: Ja, der ist XX Jahre alt und der ist in der XX Klasse.

I: Und was hast du dann gemacht?

P: Ich bin einfach weggegangen und hab es Mama gesagt.

I: Und, hat sie dir dann geholfen?

P: Ja.

I: Was hat sie denn dann gemacht?

P: Keine Ahnung, weiß ich nicht mehr.

I: O.k., gibt es noch manchmal Momente, wenn du vielleicht wütend bist oder (-)

P: Ja, also früher, da war ich immer auch traurig, wenn ich mich gestritten habe, weil, wenn man, wenn es dann der beste Freund ist, das ist ganz schön doof, weil dann hat man keine anderen Freunde mehr, wenn das Kind jetzt sauer ist, dann spielt es mit einem anderen, wenn es noch jemanden hat. Und das ist dann halt blöd.

I: Was machst du denn dann, wenn du nun so traurig bist?

P: Dann beruhige ich mich erst mal und frage, ob wir uns wieder vertragen.

I: Ja? Super. Und das klappt dann auch, dass ihr euch vertragt? Ja? Das ist ja schön.

P: Mit der Name 3 ist das mal passiert, das ist eine Freundin von mir. Da habe ich auch irgendetwas falsch gemacht. Und dann hatte sie mich gehauen und ist weggerannt.

I: Und was ist dann passiert?

P: Also, da habe ich mich auch beruhigt und hab sie gefragt. Und jetzt ist sie halt auf einer anderen Schule, und ich vermisse sie.

I: Das glaube ich. Das ist ja aber auch schade. Dann auch hier in Stadt 1? Oder ist sie ganz weit weggezogen?

P: Die wohnt hier in Stadt 1.

I: O.k., also könnt ihr euch noch manchmal sehen?

P: Naja selten, weil ich habe, montags hat sie nicht Zeit, da können wir uns nur am Wochenende treffen. Und da ist halt immer unseres zum Übernachten. Nur montags, da hat sie manchmal Zeit, wenn sie irgendwie krank ist. Aber dann kann man sich ja auch nicht verabreden.

I: Ne, das ist dann ja auch schwierig, wenn sie so richtig krank ist. Wenn es dir mal nicht so gut geht, würde ich dir das anmerken? Zeigst du das irgendwie?

P: Wenn es mir nicht so gut geht?

I: Wenn du vielleicht traurig oder wütend bist.

P: Ja, also wütend, da werde ich eigentlich also meistens rot. Wenn ich traurig bin, bin ich eigentlich immer ganz still. Weil, das ist halt (-).

I: Dann redest du nicht mehr, oder?

P: Ja.

I: Ja, o.k. Und merken deine Freunde das auch, oder, deine Mitschüler?

P: Ja.

I: Ja? Können die dir dann irgendwie helfen, wenn du traurig bist? Deine Freunde, ja?

P: Also, meine beste Freundin aus der Klasse, die heißt Name 4, die ist auch sechs. Ich spiele mit der ganz oft Pferd. Ich kann die nämlich noch auf Huckepack nehmen.

I: Echt?

P: Ja. Und eigentlich reden die mit mir einfach darüber.

I: Ah ja, und dann erzählst du denen, warum du so traurig bist? Ja? Und dann geht es dir schon besser, oder machen die dann irgendwas?

P. Ja und Name 4, die ist halt echt eine gute Freundin. Weil, die fragt auch, was los ist. Und sie sagt, soll ich das niemandem weitererzählen oder so.

I: O.k., das ist ja super. Ja, und das hilft dann schon?

P: Ja.

I: Das ist ja toll.

P: Hier hört man den Gong ja noch lauter als in unserem Klassenzimmer.

I: Das ist sehr laut.

P: Wir haben die Glocke mal verpasst, weil die so leise war.

I: Also, hier verpasst man sie nicht. Hier ist sie wirklich sehr laut.

P: Die hört man ja auch fast sogar im Lehrerzimmer.

I: Ja, wirklich. Wie ist das denn mit Lehrerin 1? Kann die dir auch helfen, wenn es dir mal nicht so gut geht?

P: Ja.

I: Ja? Was macht die denn dann zum Beispiel?

P: Meistens das gleiche, was meine Freundinnen machen. Aber (-)

I: Das heißt?

P: Also, eigentlich redet sie mit mir auch. Und da habe ich halt, bin ich eigentlich immer ganz fröhlich. Und sie fragt mich, was ich machen will. Ich habe so ein besonderes Heft da, das hilft eigentlich mir ganz doll. Wenn ich traurig bin, das ist mein XXX. Ich bin immer so schnell. Und dann habe ich halt noch was, wenn die Zeit noch nicht um ist.

I: Und was ist das für ein Heft, was machst Du da drin?

P: Also, da sind Knobelaufgaben drin.

I: Ah, o.k., das ist ja super. Und das hilft dir auch, wenn du mal nicht so gut drauf bist, dass du das machst?

P: Ja, da bin ich erstmal ein bisschen abgelenkt.

I: Ja, das ist gut.

P: Das ist ganz schön schwer irgendwie zu lesen.

I: Ach ja, das stimmt. Das sind dann so Textaufgaben, richtig? O.k., das ist natürlich schwierig.

Wie ist es denn, wenn Name 4 oder deine anderen Freundinnen mal traurig sind? Siehst du denen das an? Merkst du das?

P: Ja.

I: Woran merkst du das?

P: Also, wenn ich mal irgendwie, wenn ich mal sauer bin, gehen sie weg, wenn sie traurig sind. Und dann fragen sie halt eine andere Freundin oder was.

I: Wollen sie dann alleine sein? Oder wollen sie dann (-). Was meinst du, wollen die dann?

P: Meistens alleine, weil, eigentlich zum Beruhigen.

I: Kennst du das auch, dass du dann alleine sein möchtest, oder (-)

P: Ja.

I: Ja? Und versuchst du dann, deinen Freundinnen zu helfen, wenn es ihnen nicht so gut geht?

P: Ja.

I: Ja? Was machst du denn dann zum Beispiel?

P: Also, ich, eigentlich mache ich das gleiche, was sie bei mir machen.

I: Also, zuhören. Ja, super. Und was macht Lehrerin 1 bei denen, wenn es denen nicht so gut geht? Haben die auch so ein Heft? Oder machen die was anderes?

P: Ne, die sind nicht so schnell. Und dann (-). Die haben eigentlich ein anderes. Weil, die haben ein Heft, da kann man, das sind keine Knobelaufgaben, sondern leichte. Aber es ist so dick, so ungefähr oder so. Weil, da kann man irgendwas ausmalen. Und das geht halt schnell. Und dann kann man sich auch beruhigen. Ich hatte das früher auch.

I: Ja? Aber jetzt machst du lieber diese Knobelaufgaben?

P: Ja.

I: Das verstehe ich.

P: Ist nicht so langweilig.

I: Ihr habt ja bei euch in der Klasse auch einen Mitschüler, der ab und zu mal ein bisschen schwierig ist, nicht? Was macht der denn so?

P: Der, also, eigentlich haben wir mehrere. Name 5, das ist der Freund von Name 6, der grade bei dir war. Also, der hat mal, als Lehrerin 1 vorgelesen hat, hat der in seinen Apfel abgeknabbert und hat seinen Finger genommen und hat so huiiiiii. Ist da einfach so langgegangen und hat überhaupt nicht mitge (-)

I: Hat überhaupt nicht zugehört, oder?

P: Ne, der hat das gar nicht mitbekommen.

I: Und was macht ihr dann, wenn da jemand in der Klasse so Quatsch macht? Stört das, oder ist euch das egal?

P: Also, meistens ist es uns egal. Aber, wir sagen halt Lehrerin 1 mal Bescheid. Weil, es ist auch nicht so gut. Weil, sonst machte man das später auch, wenn man in der vierten oder so was ist. Und dann nervt das halt. Da ist man auch schon größer. Und wenn dann Erstklässler, wie bei wir, mal dabei sind, ist das manchmal ganz schön peinlich, wenn man das gar nicht mitbekommt.

I: Das stimmt.

P: Da waren wir mal bei jemandem in der 4b, und da war jemand genauso, und da haben wir voll so gemacht: "Häh, was macht der da?"

I: Und wie findest du das, wenn jemand so was im Unterricht macht?

P: Ich finde das witzig, weil, das sieht auch witzig aus. Aber eigentlich finde ich das gar nicht so gut.

I: Warum denn nicht?

P: […]. Dann ist das halt ganz schön naja.

I: Und das stört dann auch ein bisschen.

P: Ja.

I: Und was machen die anderen Schüler, die bei euch in der Klasse so ein bisschen schwierig sind?

P: Naja, Name 7, die sitzt an meinem Tisch, Name 5 auch. Die hat mal, also Name 7 hat, die macht nicht sowas wie Name 5, aber die hat Schwierigkeiten bei so Leseaufgaben. Weil, zum Beispiel, die braucht schon jetzt

noch Hilfe bei der Schreibtabellen. Die weiß nicht mal wie das „H“ aussieht oder sowas. Das ist echt (-)

I: Ja, und was ist dann mit der, wenn (-). Also, ist sie dann traurig oder was macht sie? Oder stört sie dann den Unterricht?

P: Ne meistens, sie ist traurig, weil, sie sieht halt, dass wir das schon, dass wir schon andere Hefte haben. Und dann ist sie ja traurig, dass sie das noch nicht kann.

I: Und könnt ihr ihr da irgendwie helfen, wenn sie dann so traurig ist?

P: Meistens nicht, aber ich versuche, ihr irgendwie zu helfen.

I: Und, gibt es noch andere Schüler bei euch in der Klasse, die manchmal schwierig sind?

P. Ja, Name 8, der sitzt neben mir. Also, der hat so Schwierigkeiten. Der kann das „B“ und das „D“ nicht aussprechen.

I: O.k., das ist blöd.

P: Der sagt immer „bei“ statt „drei“. „Zwei“ irgendwie so. Und das ist halt echt (-)

I: Und seid ihr dann aber in der Klasse, was passiert dann, wenn er so was laut in der Klasse sagt? Lachen dann die anderen Schüler, oder (-)

P: Ja, schon weil (-). Aber ich mach das nicht. Ich frag Name 8 erstens, und meistens mache ich das auch gar nicht. Weil, das ist halt, wenn man ihn noch nicht mal fragt, ist es halt auch (-). Da hat man Angst eigentlich, oder man

ist nervös. Weil, das ist halt, wenn man das gar nicht will, das macht einen traurig, denke ich.

I: Was macht der denn dann, wenn er ausgelacht wird?

P: Naja, Lehrerin 1 oder Lehrerin 2, die sehen das erstens und hören. Aber, wenn sie es mal nicht mitbekommen, sagen wir es ihnen auch, wenn sie wieder da sind.

I: Ja, das ist ja gut. Was ist denn, wenn bei euch jetzt in der Klasse mal jemand ganz laut ist oder den Unterricht stört. Wie ist das für euch anderen?

P: Also, es ist nervig, wenn Lehrerin 1 grade mal was erklärt und dann wird sie halt schneller wütend und erklärt das nicht nochmal. Ist bei mir sogar schon mal passiert, dass ich gar nicht wusste, was ich machen soll. Die hat mir irgendeinen Zettel hingelegt, da waren wir noch beim ersten oder zweiten Schultag. Da hat sie mir einen Zettel hingelegt. Da hat Name 9, der sitzt neben mir, der ist aus Land 1, der hat rumgeschrien, das glaubt man gar nicht. Und dann habe ich gar nicht gehört. Lehrerin 1 ist wütend geworden und hat gesagt (haut auf den Tisch). Da wusste ich gar nicht, was ich machen soll. Dann hat sie irgendwas gesagt. Ich habe nur ein paar Buchstaben verstanden. Da habe ich irgendwie einfach ein bisschen geschrieben.

I: O.k., ja. Und dann erklärt sie nicht mehr so viel, wenn sie wütend ist? Und das ist für euch anderen dann wahrscheinlich schwierig, nicht?

P: Ja.

I: Ja, das glaube ich. Und wie fühlt ihr euch, wenn da der Name 9 so schreit? Wie fühlst du dich dann?

P: Also, da habe ich manchmal Ohrenschmerzen, weil, das ist ja ganz schön laut, wenn man neben einem sitzt.

I: Ja, das glaube ich. Kommt es denn oft vor?

P: Ne, zum Glück nicht. Irgendwie, wenn wir was Witziges machen oder so was.

I: Dann kommt das eher vor?

P: Ja, aber wir machen nicht so oft was Witziges.

I: Und, wird dann der Unterricht lange gestört, oder ist das kurz?

P: Das ist eigentlich, wenn man einmal schreit, oder so was. Eigentlich nicht so lange, aber (-)

I: Das heißt, danach könnt ihr dann wieder normal weiter machen? O.k., gut.

P: Aber es ist halt auch manchmal, ganz selten zum Glück, aber dann ist es schon ganz schön lang.

I: Ja? Und was passiert dann, wenn das so lange ist? Was macht ihr dann?

P: Also, das ist halt blöd für uns, weil, dann können wir, können wir es nicht so richtig wissen, was da ist, so.

I: Ja. Hast du denn eine Idee, warum der Name 9 manchmal so schreit, oder warum der sich so verhält?

P: Also, aus Land 1, da darf (-). Da gibt es ja eigentlich gar keine Polizei. Und da, (-). Hat er gesagt, der hat uns mal gesagt, dass man da hauen darf. Wahrscheinlich hat er das

davon. Da haben die Schüler auch immer rumgeschrien. Wahrscheinlich hat er das bei denen abgeguckt.

I: Ja, wahrscheinlich. Aber, könnt ihr ihm denn so ein bisschen helfen, dass er sich hier wohler fühlt in der Klasse?

P: Naja schon, aber wir haben es ihm schon mal gesagt, aber der vergisst es halt immer.

I: Das heißt, da ändert sich auch nicht so viel?

P: Ja. Und wir haben es mal versucht. Aber dann ist er wieder ausgerastet. Einmal ist er so schrecklich ausgerastet, der ist aus der Tür gerannt und hat, hat es als Pause empfunden.

I: Und da ist er einfach gegangen?

P: Ja.

I: Ach, und was habt ihr da gemacht?

P: Wir haben weitergemacht.

I: Und Lehrerin 1?

P: Die war im Gruppenraum mit einer anderen Frau. Die hat das gar nicht mitbekommen, weil, der ist weggeschlichen wie ein Eisbär.

I: Und, ist er dann auch wiedergekommen?

P: Als die Pause geklingelt hat, da mussten wir ihn suchen. Aber er war nicht vom Schulgelände weg, aber er hat sich irgendwie versteckt, oder so was. Der hat uns fast mal angegriffen, als wir ihn holen wollten. Da mussten wir Lehrerin 2 losschicken.

I: Der hat euch angegriffen?

P: Ja, fast.

I: O.k., aber die Lehrerin konnte euch dann helfen?

P: Ja.

I: O.k., gut.

P: Wir haben es mal mit der ganzen Klasse versucht. Wir sind hingegangen und Lehrerin 2 hat leise zugeschaut, und dann musste sie uns sogar helfen. Wir sind 19 Kinder, eigentlich sind wir 20, mit Name 9.

I: Ja, und ihr habe den aber nicht alleine beruhigen können?

P: Ne, das ist echt schwer, ihn bändigen zu können.

I: Was habt ihr denn versucht, habt ihr gemacht?

P: Also, wir haben gesagt, „Name 9, komm mal mit, wir wollen jetzt weitermachen." Hat er uns so, der hat uns so angeschrien, wir haben alle Ohrenschmerzen fast bekommen.

I: Und nicht aufgehört?

P: Ne.

…

Transkription des Interviews mit Student 6

<u>Legende:</u>

Interviewer=„I“; Proband=„P“

Pausen=(...)

unverständliches Wort=((unv.Wort))

Wort- und Satzabbrüche= (-)

- Anonymisierungen=[…]

I: Zunächst einmal, in welche Klasse gehst du denn?

P: In die erste Klasse.

I: Warst du hier auch schon in der Vorschule?

P: Ja, ich war in der Werte.

I: Das heißt, du kennst dich hier an der Schule schon so ein bisschen aus. Und gehst du denn gerne zur Schule?

P: Ja.

I: Ja? Also, du bist gerne hier?

P: Ja. Nur wenn es grade gestern, oder grade heut einen Streit in der (-), bin ich mir nicht so sicher, ob ich morgen wieder komme.

I: Hattest du heute einen Streit?

P: Nein, nur manchmal, wenn es Streit gibt.

I: Ja, das kann ich verstehen. Passiert das denn öfter mal, dass du dich mit jemandem streitest?

P: Also, nicht jeden Tag. Also, ich glaube, gestern war ein Streit, ein kleiner Streit, so ein Missverständnis.

I: Was ist denn da passiert? Magst du mal erzählen?

P: Also, da wollten Name 1 und ich, die war ja auch grade mit dir, und da wollten wir beide spielen. Und dann kam Name 2. Und dann hat sie gesagt „Ich spiele mit.“ Und dann wollten Name 1 und ich eigentlich alleine zusammen spielen. Die spielt ja jeden Tag fast mit mir. Dann möchte ich auch

mal alleine spielen. Eigentlich möchte ich gar nicht, dass ich jetzt mit Name 2 befreundet bin. Aber die sagt das so.

I: Du willst gar nicht mit ihr befreundet sein?

P: Ne, aber die „(-) wir sind beste Freundinnen.“ Kann ich auch mal reden? Aber die lässt mich gar nicht reden, weil die so blablabla.

I: O.k., und was habt ihr dann gemacht?

P: Und dann haben Name 1 und ich eigentlich gesagt, „Name 2, stop mal.“ Und dann hat sie gesagt "ich hol aber gleich die Pausenaufsicht". Und dann wir beide so „Name 2, hör uns mal zu.“ Und die so "nahnahnahnahnah". Und dann hat sie gesagt, „ich gebt euch jetzt nur noch eine Chance.“ Und dann wir beide so „O.k.“. Und heute noch, dann kommt die auch immer, sagt die ja immer, sie sagt, sie denkt sich schon vorher immer so Sprüche aus, die sie zu uns sagt.

I: Was sagt sie denn für Sprüche zu euch?

P: Also, sie sagt zum Beispiel, „Wenn ihr noch einmal das und das macht, dann gehe ich zur Pausenaufsicht“.

I: O.k., und was macht ihr dann, wenn sie so was sagt?

P: Dann sagen wir „Name 2, stop mal, ich kann dir das gleich erklären“. Egal, wie gut wir das erklären, sie sagt „Nein, das macht ihr nicht. Ihr wollt das jetzt nur nicht so sagen“.

I: O.k. Und wie habt ihr das dann gestern gelöst? Was habt ihr dann gemacht?

P: Also, dann haben wir gemacht, dass Name 2 dann doch noch mit uns spielt.

I: Und dann habt ihr zu dritt gespielt? Und war das dann o.k. oder weißt du es nicht?

P: Also, das war ein bisschen o.k. Aber, dann hat man es auch ganz schnell wieder vergessen.

I: Das ist ja ganz gut. Gibt es denn noch andere Momente, Momente, in denen du dich nicht so gut fühlst? Wenn du traurig bist oder wütend.

P: Eigentlich, nur manchmal, wenn ich meine Hausaufgaben vergesse. So, wie es mir heute, gestern passiert ist. Da haben die gesagt „Tu bitte dann (-)“. Also, dann habe ich gefragt, wo soll man (-). Dann habe ich meine Klassenkameraden gefragt, wo soll man denn das Heft hinlegen? Dann habe die gesagt „Ins Geheimfach“. Dann habe ich es ins Geheimfach gelegt. Und dann bin ich gegangen. Aber, das Buch lag noch im Geheimfach. Dachte ich so, o.k., das soll jetzt ja so sein. Dachte ich, die haben mir bestimmt schon andere Hausaufgaben gegeben. Hatten die gar nicht.

I: Ach so, und dann waren die Hausaufgaben eigentlich in dem Heft?

P: Ja, und dann habe ich eigentlich, dann habe ich heute schnell das Heft genommen. Und dann mache ich das zu Hause noch einfach mal nach. Das geht ja auch.

I: Was machst du denn, wenn du merkst, oh ich bin jetzt irgendwie traurig, oder ich habe mich erschrocken oder so.

P: Dann sage ich, "och Mama, wieso hast du nicht mit aufgepasst?" Dann habe ich auch, dann haben Name 3, hat die gesagt „Wollen wir was malen?“. Dann haben wir was gemalt. Also, so ein bisschen gespielt, Playmo gespielt,

ganzes Zimmer voll mit Playmo. Da habe ich mir schon sehr oft wehgetan, weil das ganze Playmo auf dem Boden liegt.

I: Oh und wenn man dann da drauftritt. Ja, das kenne ich auch. Wenn du dann mit deiner Schwester was malst, geht es dir besser?

P: Ja, also, da war gestern noch Name 4, meine Freundin aus der zweiten, und dann haben wir da gespielt.

I: O.k. Und, würde ich denn sehen, wenn es dir nicht so gut geht? Zeigst du das anderen?

P: Also, dann zeige ich nicht das sehr deutlich, sondern dann denke ich nur im Gefühl. Also ich sehe das ja gar nicht, weil ich dann nicht zum Spiegel schau. Also, innendrinne, wenn man in mich rein schaut, aber das geht ja gar nicht, dann sieht man es schon. Aber ich glaube, wenn man so auf den Schulhof geht, ein bisschen an meinem Gesicht.

I: Guckst du dann ein bisschen traurig?

P: Ja, dann ich so (-).

I: Aber, du zeigst das nicht doll?

P: Ne.

I: Aber, redest du dann mit jemandem drüber, wenn es dir nicht so gut geht?

P: Ne, dann eigentlich nicht.

I: Nein? Aber, wie könnten dir deine Freundinnen zum Beispiel helfen, wenn es dir nicht gut geht? Können die da irgendwas machen?

Student 6

P: Ich habe da noch nicht mal nachgedacht.

I: Bist du dann lieber alleine?

P: Ja, dann denke ich so, ich möchte auch mal alleine spielen. Aber zum Beispiel da, als ihr grade gesprochen habt, da wollte ich in der Pause drinbleiben. Und dann hat Name 2 gesagt „Ich bleib auch drin“. Kann ich nicht alleine gehen, weil die dann immer „Ich bleibe auch drin“. Egal, wo ich hin gehe. Nur, wenn ich zu Name 4 und Name 5 gehe, das sind meine Freunde, die sind schon in der vierten, dann sagt Name 2, hat sie so mitbekommen, dass die Freunde irgendwie gesprochen haben. „Also Name 2 soll nicht mitspielen, weil wir die noch nicht so kennen". Und dann, immer, wenn ich dahingehe, dann kommt die nicht mit.

I: Und jetzt wolltest du eben auch alleine spielen, weil du ein bisschen traurig bist?

P: Ja, dann will ich eigentlich alleine spielen. Dann kommt schon Name 2 und sagt „Was wollen wir spielen? Wollen wir Pferd spielen? Und dann holt die schon das Springseil so zum Pferd.

I: Könntet dir denn eine andere Freundin helfen, wenn es dir nicht so gut geht?

P: Eigentlich Name 6, eigentlich Name 6 und ja das war's, nur Name 6. Aber Name 6 spielt ganz oft mit jemand anderes, mit Name 7. Name 7 find ich nicht grad so toll und dann kann ich nicht so gut viel mit der spielen.

I: Das ist schade, nicht? Und was könnte Name 6 machen, damit es dir besser geht?

P: Also, dann können wir zusammenspielen. Name 1, die ruckelt auch so doll an mich ran. Das tut mir immer ganz doll

weh. Ich sag auch schon öfter, stop, stop, stop. Die hört nicht auf.

I: Und was machst du dann?

P: Dann sage ich "Oh Name 1 stop“ und dann hört die auch von alleine auf. Dann kann ich nur hoffen, dass die gleich aufhört.

I: Ja, und wirst du dann auch traurig oder böse?

P: Ja, das tut mir dann weh eigentlich.

I: Redest du dann mit Lehrerin 1 darüber?

P: Also. eigentlich nicht, dann fragt sie nur manchmal so „Wie war die Pause?“ Dann zeige ich zum Beispiel manchmal so oder so. Manchmal sage ich auch „gut“, weil die Pause dann gut ist.

I: Und wenn du grade so ein bisschen traurig bist, was wünscht du dir denn dann?

P: Dann wünsch ich mir eigentlich, dass ich dann einen Moment alleine habe. Dann kommen schon so viele, dann kommt Name 4 und dann kommt Name 2 und kommt an "sollen wir spielen…".

I: Und was würdest du machen, wenn du dann alleine bist?

P: Also, dann würde ich mich eigentlich auf die Bank setzen und erst einmal in Ruhe ausatmen. Weil, ich kann das ja nicht, weil da Name 2 immer kommt.

I: Und eigentlich willst du nur ein bisschen deine Ruhe haben.

P: Ja.

I: Wie ist denn das bei deinen Freundinnen, wenn die mal traurig sind? Siehst du denen das an?

P: Also, dann sehe ich bei Name 2, dass sie dann so ein bisschen langsamer geht. Und man sieht das auch so ein bisschen an den Augen. Etwa sind die so: ___

I: Ja, das sieht man dann sicher.

P: Aber die anderen da kenne ich gar nicht so gut, wie die traurig sind.

I: Dann siehst du das nicht so? Und wenn die vielleicht wütend sind, siehst du das?

P: Also bei Name 8, das sehe ich dann, weil dann wird der so ganz schnell wütend, nur wenn ihn andere ärgern. Das war auch einmal, da hat Name 2 und Name 1, Name 2 oder Name 1, ich weiß es nicht mehr, gesagt „Name 8, Dicker, Dicker, Dicker. Alle sagen Dicker“.

I: Dicker?

P: Ja, Dicker. Dann habe ich gesagt, dann habe ich gar nichts gemacht, und dann hat er mich auch nicht geärgert. Aber wenn die anderen ihn ärgern, wenn er dann das Gefühl hat, dann dreht er auch auf.

I: Ja, ja, was macht der denn dann?

P: Also dann macht er da, eigentlich, dann wird er ganz wütend und dann macht er auch schnell, ärgert er andere Kinder.

I: Was macht er dann, wenn er die ärgert?

P: Dann, zum Beispiel, dann stellt er ihnen ein Bein oder tut Sachen, die die anderen Kinder gar nicht mögen.

I: Und was machst du dann, wenn so was passiert?

P: Denn stehe ich immer ganz still und dann schaue ich erst einmal, was dann die anderen Freunde machen, damit ich nachher die anderen erzählen kann, was die dann (-). Und dann, zum Beispiel, Name 8 läuft ganz schnell, dann jagt Name 8 zum Beispiel Name 2. Das mag sie nicht so gerne. Der sucht sich dann so Sachen aus, die die Kinder gar nicht gerne mögen.

I: Ah, o.k. und dann ärgert er die? Macht er das im Unterricht oder auf dem Schulhof?

P: Also, der hat das letztes Mal auf dem Schulhof gemacht. Aber manchmal macht er es auch im Klassenraum. So, öfter im Klassenraum.

I: Und wie fühlst du dich dann, wenn so was passiert?

P: Also, dann fühle ich mich eigentlich nicht so gut, weil dann denke ich so, was könnte dann passieren? Oder könnte ja passieren, dass sich dann einer sehr doll weh tut. Dann denke ich so hmm. Dann bin ich auch schon, kann ich Name 8 schon verstehen. Aber Name 2 und Name 1 eigentlich auch. Dann bin ich mir nicht so sicher „Hä, soll ich jetzt Name 8 helfen oder soll ich dem helfen? Weiß ich gar nicht genau".

I: Aber Name 8 kannst du auch verstehen?

P: Ja.

Student 6

I: Was glaubst du denn, warum der das so macht?

P: Also, der ist eigentlich ein ganz lieber Mensch, aber wenn ihn andere ärgern, dann findet er das nicht so toll, und dann will er die anderen auch ärgern. So, dass das für ihn gerecht ist. Weil, er kommt ja auch vom anderen Land, da ist auch Krieg. Hat er so ein bisschen von denen abgeschaut.

I: Und glaubst du, man könnte ihm irgendwie helfen?

P: Ja, wenn man ihn, wenn zum Beispiel Name 2, Name 1 nicht ärgern, dann ist auch besser.

I: Wenn die ihn nicht ärgern?

P: Ja, aber manchmal macht er auch so, dass er zu den Lehrerinnen, zu den neuen (-) hat er mal zu Lehrerin 1 gesagt, du bist blöd. Ich möchte nicht in diese Schule.

I: Und was macht die dann?

P: Dann sagt die „Name 8, das reicht“. Und wir haben so Klammern und dann setzt man die so runter. Wenn man auf „Blitz“ ist, dann werden die Eltern benachrichtigt. Also, ich bin da eigentlich nie (-) Auf Regenwolken startet man und dann geht man entweder hoch oder geht man zurück. Wenn man auf Sonne ist, dann hat man ein Steinchen. Wenn das voll ist, dann macht man einen Ausflug. Und Name 8 hat schon mal Bein gestellt oder geschlagen, durfte er beim Ausflug nicht mitkommen.

I: Das ist ja blöd. Und findet er das auch schade, oder (-).

P: Ja, der sagte dann „schade“. Aber meine Mutter sagt dann immer, der weint dann zu Hause.

I: Deine Mutter sagt, er weint zu Hause?

P: Ja, da weint der ganz doll. Dann in einer anderen Klasse. Dann musste der da aber auch arbeiten.

I: Das heißt, dann geht es ihm auch nicht gut.

P: Ne, dem geht es nicht so gut.

I: Und wenn er dann auf dem Blitz ist, findet er das auch schade oder ist ihm das egal?

P: Also, das ist ihm manchmal egal. Manchmal, ganz oft ist es ihm dann egal. Dann sagt er auch manchmal, wenn er grade auf Halbsonne ist, sagt er, "ich möchte auf Blitz wandern". Sagt er auch manchmal.

I: Und was ist so bei euch in der Klasse, wenn Name 8 jemanden ärgert. Was passiert dann?

P: Also, dann, alle mögen Name 8 nicht so gerne. Und Name 9, ich kenn die ja nicht so gut, weil die aus einem anderen Land kommen. Ich finde die eigentlich beide ganz sehr nett. Weil, Name 9 ist ja auch mein Nachbar, der neben mir sitzt. Ich finde eigentlich den ganz schön: Die anderen Kinder sehen ihn nur, wenn er grade kämpft.

I: Das heißt, sonst kriegen die gar nicht so viel von denen mit und dann nur das.

P: Die sehen nur eigentlich nur die schlechten Seiten. Aber ich sehe auch sehr oft die guten.

I: Das heißt, du verstehst dich ganz gut mit den beiden? Und kannst du denen dann auch manchmal so ein bisschen helfen, wenn es ihnen nicht so gut geht.

P: Ja, ich sehe die nicht so oft, weil, Name 9 spielt Fußball und Name 8, der spielt, glaube ich, mit seinem Freund, spielt aber auch manchmal Fußball. Ich treffe die nur manchmal und dann braucht er ja grade keine Hilfe. Dann geht er nur ganz normal vorbei und dann denke ich so, o.k., der braucht grad keine Hilfe.

I: Sieht man das denn schon, wenn vielleicht ein guter Tag oder ein schlechter Tag ist bei denen?

P: Nein, das sieht man eigentlich nicht. Das kommt dann so ganz überraschend.

I: O.k. Und stört das dann manchmal auch den Unterricht, oder ist das eher in den Pausen?

P: Eigentlich stört das auch manchmal im Unterricht. Manchmal kommt er dann an, also nicht manchmal, sehr oft, kommt er dann an und dann kommen andere an und sagen „Name 8 hat zu mir das und das gemacht und das und das und das und das. Obwohl das Name 8 eigentlich gar nicht extra gemacht hat. Dann sagt Name 8 „Aber, zum Beispiel Name 2 hat auch zu mir gesagt Dicker, Dicker, Dicker“.

Dann sagt Name 2 „Nee, nee“. Dann sieht man das nicht so gut bei Name 2, weil „Nee, nee“. Also, (-)

I: Und dann kriegt er die Schuld?

P: Ja, dann bekommt er die Schuld die ganze Zeit.

I: Und wie ist das für ihn?

P: Für ihn ist das meist, sehr oft, also ich fühle das so ein bisschen, dass er dann, „Man oh, jetzt bekomme ich die Schuld, obwohl die eigentlich Schuld ist“.

I: Dann ist er auch traurig wahrscheinlich.

Student 6

P: Dann ist er traurig auch, aber man sieht es nicht so.

I: Glaubst du denn, man könnte auch ihm da irgendwie dann helfen?

P: Also, ich glaube, man könnte ihn nicht ärgern und dann könnte man auch fragen „Name 8, möchtest du mit mir spielen?“ und so. Aber der läuft ja ganz oft, ich sehe ihn eigentlich nur in der Pause, wenn er alleine rumläuft. Dann läuft der da eigentlich ganz zufrieden rum. Aber ich sehe ihn nur alleine.

…
