# Supplementary material for: Mental health and cultural and linguistic diversity as challenges in school? An interview study on the implications for students and teachers
Source: PLoS One. 2020 Jul 20;15(7):e0236160. doi: 10.1371/journal.pone.0236160 (PMC7371207; doi:10.1371/journal.pone.0236160)
Supplement: Attachment — Submitted filename: Response to Reviewers.docx [file pone.0236160.s004.docx]

**Response to Reviewers**

**Editor**

Dear Mr. Stavropoulos,

thank you for allowing us to revise our manuscript and submit it again to PLOS ONE. The following table replies to the points you raised in your E-Mail directed at us.

| If you would like to make changes to your financial disclosure, please include your updated statement in your cover letter. | We have edited the financial disclosure and included it into the cover letter:  The author was supported by the Hans-Böckler-Foundation through a doctoral scholarship.  https://www.boeckler.de/index.htm. There was no additional funding for this project. |
| --- | --- |
| To enhance the reproducibility of your results, we recommend that if applicable you deposit your laboratory protocols in protocols.io, where a protocol can be assigned its own identifier (DOI) such that it can be cited independently in the future. | Since this is a qualitative study, we do not have a laboratory protocol. If you would like us to upload the interview guidelines, we would be happy to do that. |
| Please note while forming your response, if your article is accepted, you may have the opportunity to make the peer review history publicly available. The record will include editor decision letters (with reviews) and your responses to reviewer comments. If eligible, we will contact you to opt in or out. | If the article is accepted, we would be happy to make the review process publicly available. |
| 1. Please ensure that your manuscript meets PLOS ONE's style requirements, including those for file naming. | We have checked the PLOS ONE formatting requirements for the manuscript and for file naming. |
| 2. We note that you have indicated that data from this study are available upon request. PLOS only allows data to be available upon request if there are legal or ethical restrictions on sharing data publicly. | We will upload the parts of the anonymised transcripts that are relevant for this study as Supporting Informaton files. However, the transcripts are all in German. |

**Reviewer #1:**

Dear Reviewer #1,

thank you very much for thoroughly reviewing our manuscript and your interesting and kind comments. In the following table we reply to the points you have raised about the manuscript. We hope that the responses address your concerns appropriately. We would be grateful if you would let us know which parts of the manuscript need further revision.

| The paper overall was a good read and informative. It contained a valuable perspective and tried to investigate a new aspect of the connection between mental health problems and cultural & linguistic diversity and the school environment. The Method and Results section were particularly well written and laid out. The tables with the codes and examples were useful to evaluate the qualitative information and understand what aspects of the categories were being explored. The citations provided an insider account of what the operational and practical explanations of those categories looked like. | Thank you very much for this comment. |
| --- | --- |
| The introduction and rationale building up to the research could be more specific. Since cultural diversity is an important aspect of the study, it would be interesting to know what the country of origin is for the research quoted in the literature review. | We have specified the text according to your hints and included the country of origin for most studies in the literature review. |
| I would also be interested in understanding the magnitude of effect for some of the research quoted (line 65: what is “negatively impacts”? | We have included the magnitude of the effects and a description of what “negatively impacts” means in the specific research:  Line: 65 ff  “Some research shows that including a disruptive pupil into the school-cohort lowers the academic achievements of their classmates [26-28]. In a US sample emotionally disabled children decreased reading and maths grade of their peers by over 10 percent of a standard deviation [27]. |
| line 72: “disruptive students can affect the entire classroom”- what does "affect" mean? Where was this research done? What age group is that specific to? Is disruptive in the original study in the context of mental health problems?). | We have expanded the information on this study to include the country of origin, the age group of the students and the context of interest. We explain the effect as it was done in the original study:  Line: 74 ff (formaly line 72)  “A qualitative study with teachers from Australia investigated the teachers’ perspective on externalising and internalising problems in high school students. The teachers identified children with externalising symptoms as being more disruptive which can impact the entire classroom [34] and cause teachers’ stress [34, 35].” |
| I felt that overall the introduction lacked specific information and overlooked some important aspects of the literature that may contribute to a more meaningful discussion. | Du to your remarks, we have added specific information in the introduction. For example, we included the concept of intercultural competence. Furthermore, we have expanded the evidence on challenges teachers face in diverse classrooms. |
| I would like to know the gender for the experts and the cultural background for the teachers in the classroom. | We have included the gender for the experts (line 241) and the country of birth for the teachers (line 237) in the results section. |
| Did the teachers generally identify with the majority culture in the classroom? Does the relationship across variables and aspects like patience and empathy vary when the teacher is of a minority cultural background? | This is a remarkably interesting aspect. Unfortunately, we did not systematically assess the culture that the teachers identify with. Therefore, we cannot evaluate the influence of their cultural background on variables like empathy or patience. As this is an important variable to consider, we included this aspect in the discussion:  Line: 556 ff  “The cultural background of the interviewed teachers was not assessed in this study. However, the culture of a teacher can influence the relationship with the students in various ways. Studies from the United States show for example that teachers rate children of a different ethnicity as more negative [88, 89] and have lower expectations for their educational success [90]. Therefore, assessing cultural background and cultural attitudes of teachers might be an appropriate way to control possible confounders in similar studies.” |
| In the discussion section, it might be important to just mention the typical set up of a classroom since burden and time management skills are of importance when discussing teacher perspective. I would have thought primary classes usually have an assistant teacher? Is that not true for German primary schools? That may have some implications on just the reader understanding the larger picture a little better. | We have included a paragraph on a typical German primary school classroom in the discussion:  Line: 441 ff  “The interviews focused on German primary schools. Primary school classes in the here included cities have between 17 – 26 students [74]. The distribution of teacher assistants and pedagogues depends on the amount of special needs students and of students in total in a school and classroom [75]. In most cases, teachers are only supported by an assistant for a few hours a week [76].” |
| The conclusion and outlook section are very well written and succinct. It would be valuable to elaborate on what intercultural competence (line 513) entails and if there is any reference to relevant studies that can be brought up in the introduction to introduce this idea. | We have included an abstract on intercultural competence in the introduction to illustrate this concept and set it in the context of schools.  Line: 110 ff  Besides, we included a reference to this research in the discussion:  Line: 566 ff |
| Overall, it is an interesting paper that is fun to read and neatly ties multiple variables discussed in the paper. | Thank you again for your positive and constructive feedback. |

**Reviewer #2**

Dear Reviewer #2,

thank you very much for reviewing my manuscript and for your valuable and interesting comments. In the following table we address each of your concerns. We hope our revision improves the manuscript in the way you had intended. We would be happy if you would let us know what aspects of the manuscript need further revision.

| In general the research was a qualitative examination of the diverse effects of mental health issues and cultural and linguistic diversity on students, classmates and teachers yet these two topics were not examined together in any way to identify if they overlapped. | We did not discuss the overlap of these two subjects, because it was not the primary focus of our research. The aim of the manuscript was to identify the challenges and resources that exist in schools for teachers and students due to mental health issues and cultural and linguistic diversity.  The topic that you raise is also an important and interesting question that should be addressed in a study focusing on this aspect alone. |
| --- | --- |
| Recruitment: Although the researchers have indicated purposive sampling was used to select participants/schools its unclear what judgments were used limiting the ability for the results to be tested/repeated. | We have included the following in the description on teachers’ recruitment:  Line: 163 ff  “The schools were contacted via mail or personal contact. Teachers that might be interested in participating in the interview were recommended by their colleagues or the head of school.”  The following was included in the sampling description for students:  Line: 167  “Students were supposed to have experience with mental health issues in classmates and diverse classrooms. Besides, they should variate in gender and age.” |
| It is unclear how many culturally and linguistically diverse students were at each school and how much experience each teacher had (number of years etc.). | We have included the following information for the three schools:  Line: 156 ff  “Schools were chosen from three different locations: One is located in a small rural town with around 40 % of students that have a migration background. The other two are in a large city. One is situated in a high-income suburb where only 5 % of students have a migration background. The other one is in a low-income suburb where over 70 % of students have a migration background.”  The average and range of the teachers’ experience is included in the results section (*M* = 17.57 (range = 2 – 42) years of teaching experience, line: 236). To ensure data privacy we cannot include the number of years for each teacher individually.  We did not systematically assess other variables for describing the teachers’ experience. |
| Data collection: one-on-one interviews were used except for one interview in which a teacher and external were questioned simultaneously. This inconsistency is a confound which could have been avoided and the reason for it is not explained. | We included the following explanation for this confound.  Line: 182 ff  “Only one interview was conducted simultaneously with an external and a teacher because the external had invited the teacher without advanced notice to the interviewer.”  Even though this setting is different to the one-on-one interviews we feel like it did not change the teachers and externals answers. In fact, in some cases it incited the memory of the interview partners enabling a rich outcome. |
| Sample: Although the research was qualitative in its approach the sample size was very small and not representative in the case of primary school teachers. | The sample size is indeed not large. We tried to maximise the variation of primary school teachers. Yet, we do not assume the results are representative for all primary school teachers. We have deleted any comments that suggest representativity and have included the following statement in the discussion:  Line: 530 ff  “The sample size of this study is relatively small. However, despite the great diversity of interviewed teachers and externals there was extensive overlap between their answers so that more interviews would most likely not have produced a lot of new information. Besides, there is evidence that six interviews are sufficient to identify the main themes of a topic [86].” |
| No details were provided on the gender of students selected nor how they were selected. This limited the inferences which the researchers later made in their discussion. | We expanded the information on the selection process for students.  Line: 167 ff  “Students were recruited through contacts made with the interviewed teachers. Students were supposed to have experience with mental health issues in classmates and diverse classrooms. Besides, they should variate in gender and age. No student refused to participate in the interview.”  Regarding the gender aspect we included the following in the results section:  Line: 231 f  “In each grade two girls and one boy were interviewed.” |
| Furthermore, despite the topic of interest relating to children with mental health issues and cultural and linguistic diversity, none of the children interviewed fell into these categories. | We absolutely agree that this is a limitation of this study. It would have been of great value to interview children with mental health issues and of cultural and linguistic diversity. We have included an explanation for this limitation in the discussion.  Unfortunately, access to children with mental health issues requires special precautions that we could not meet in this study. The same goes for children with cultural and linguistic diversity. We were not able to receive parental permission to interview children from these groups due to linguistic challenges or data privacy.  We have included the difficulty of receiving parental permission in the discussion:  Line: 523 f  “Despite multiple efforts, it was not possible to receive parental permission for interviews with students of different cultural and linguistic backgrounds.” |
| Discussion: The authors appear to imply that the answers provided by the primary school teachers "are still representative" of general population of primary school teachers despite the lack of representation of male teachers sample and despite the extremely low number of teachers interviewed (n=7). This is an inference contrary to common research/statistical knowledge. | We have deleted the comment on representativity of the data and adjusted the section of the discussion.  Line: 447 ff  “Though the majority of primary school teachers in Germany are female (89.5 %) [77] the experiences of male teachers with challenges at school might differ from the ones of female teachers.” |
| Conclusion and outlook: The authors state that, "It would be advisable to include classes on mental health and intercultural competence into the teacher formation to prepare them for dealing with these challenges," without citing any research (their own findings or otherwise) to support this statement. | We have included research to support this statement.  Line: 562 ff  “Mental health issues in children inflict a variety of challenges on teachers and students. Like studies on mental health literacy in teachers [91, 92] and on the teachers’ role in dealing with mental health issues in school [30, 34] the results of this study suggest that it would be advisable to include a mental health training into the teacher formation to increase their confidence and ability in dealing with mental health problems in students. Besides, the effects of cultural and linguistic diversity identified here lead to the assumption that teachers could benefit from an intercultural competence training enabling them to encounter the cultural differences of their students positively and act interculturally appropriate [63, 64].” |
| The reviewer could not locate the data underlying the findings | We will upload the transcripts of the interviews as Supplement Information. |
